# Disruption of normal stem cell function and transmission of myelodysplastic syndrome by self-renewal of committed myeloid lineage cells

**DOI:** 10.1016/j.stemcr.2025.102571

**Published:** 2025-07-03

**Authors:** Yang Jo Chung, Ryan Bertoli, Dengchao Cao, Robert L. Walker, Yuelin Jack Zhu, Paul Meltzer, Peter D. Aplan

**Affiliations:** 1 Genetics Branch, Center for Cancer Research, National Cancer Institute, National Institutes of Health, Bethesda, MD, USA; 2 Myeloid Malignancies Program, National Institutes of Health, Bethesda, MD, USA

**Keywords:** myelodysplastic syndrome, MDS-initiating cell, stem cells, NHD13, kit, hematopoiesis

## Abstract

The ineffective hematopoiesis of myelodysplastic syndrome (MDS) suggests that hematopoietic stem and progenitor cells (HSPCs) are defective. Here, we demonstrate that *NUP98::HOXD13* (*NHD13*) MDS mice have significantly decreased functional HSPCs. Moreover, in contrast to wild-type (*WT*) bone marrow (BM), lineage-positive (Lin^+^) BM cells from *NHD13* mice have self-renewal potential. Specific subsets of *NHD13* Lin^+^ cells that express B220 and Kit antigens were able to self-renew and generate MDS in *WT* recipients. Although this unique B220^+^Kit^+^ phenotype could be found in *WT* as well as *NHD13* BM, the population was markedly increased in *NHD13* BM. Further characterization using Mac1 and Gr1 markers revealed that both Mac1^+^Gr1^+^B220^+^Kit^+^ and Mac1^−^Gr1^−^ B220^+^Kit^+^ populations showed self-renewal and led to an MDS phenotype in *WT* recipients. Taken together, these findings demonstrate that as normal hematopoiesis derived from typical HSPCs decreases in *NHD13* mice, committed hematopoietic progenitor cells proliferate, self-renew, and initiate MDS.

## Highlights

• MDS can be transmitted to wild-type mice by transplantation of NHD13 bone marrow
• The MDS-initiating cell (MIC) is in the lineage-positive (Lin^+^) population
• The most efficient MICs are B220^+^Kit^+^
• Transformation of MDS to AML in this model is linked to acquired somatic mutations

## Graphical abstract

**Figure:**
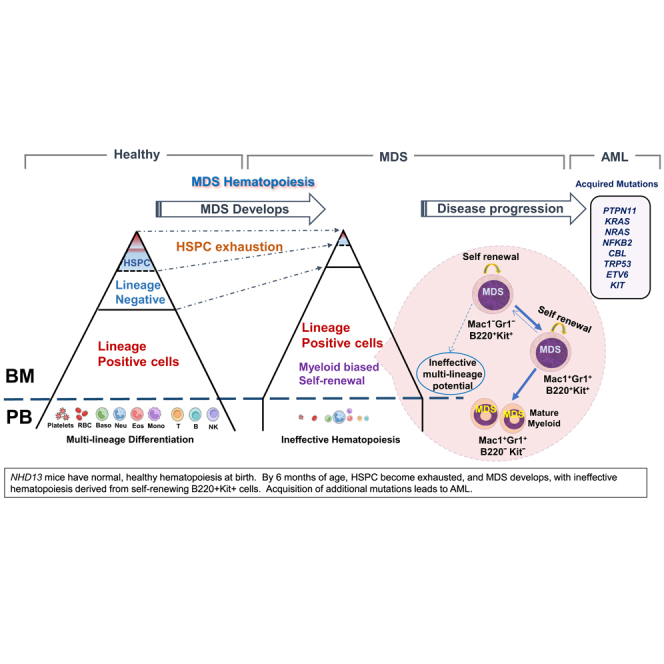

## Teaser

It is generally thought that lineage-committed or terminally differentiated hematopoietic cells have lost the ability to self-renew. Surprisingly, the most efficient MDS-initiating cell in a murine model for MDS is a lineage-committed cell that retains an indefinite capacity to self-renew.

## Introduction

The myelodysplastic syndromes (MDSs) represent a heterogeneous group of clonal hematopoietic stem cell (HSC) disorders and are a significant cause of morbidity and mortality. In general, the incidence of MDS increases with age, and the median age at diagnosis is 70 years old (Maynadie et al., 1996). Although some forms of MDS can be considered a pre-leukemic condition, MDS represents a disease entity that is distinct from acute myeloid leukemia (AML) (Nimer, 2008). Using xenograft mouse models, AML-initiating cells have been thoroughly characterized (Bonnet and Dick, 1997; Hope et al., 2004; Lapidot et al., 1994; Shlush et al., 2014). However, isolation and characterization of MDS-initiating or stem cells using xenotransplantation of human cells from patients with MDS has been challenging due to relatively low-level engraftment of MDS cells, even in severely immunodeficient mice. Moreover, the engrafted cells do not consistently generate the characteristic features of MDS in mice (Benito et al., 2003; Kerbauy et al., 2004; Nilsson et al., 2002; Thanopoulou et al., 2004), possibly because the murine hematopoietic system does not produce an environment (including cytokines, chemokines, and cell-cell interaction) that will support the entire spectrum of human hematopoietic differentiation. Therefore, to avoid issues caused by immunodeficiency and cross-species barriers, we used murine MDS cells within the context of a murine host to identify MDS stem cells.

We employed a mouse model of MDS based on the expression of a *NUP98::HOXD13* (*NHD13*) fusion gene, which was initially cloned from a pediatric patient with MDS (Raza-Egilmez et al., 1998; Slape et al., 2008). This model faithfully recapitulates all of the key features of MDS, including peripheral blood (PB) cytopenias, maturation arrest, bone marrow (BM) dysplasia, and transformation to acute leukemia in approximately 60% of mice (Lin et al., 2005). Using this model, prior studies have demonstrated that MDS can be transferred to healthy recipients via HSC transplantation (HSCT), suggesting the existence of an MDS stem cell (Chung et al., 2008).

In this study, we demonstrate that normal hematopoiesis rapidly diminishes as *NHD13* mice age and can be replaced by hematopoiesis derived by extended self-renewal of committed, lineage-positive (Lin^+^) cells. We further demonstrate that hematopoiesis derived from these Lin^+^ cells can be transplanted to wild-type (WT) recipients, leading to MDS and extensive self-renewal of these committed cells, culminating in AML transformation after an extensive incubation period (up to 27 months) and acquisition of mutations in collaborating genes *in vivo*.

## Results

### *NHD13* mice show progressive pancytopenia and loss of hematopoietic stem and progenitor cells with age

**Figure 1:**
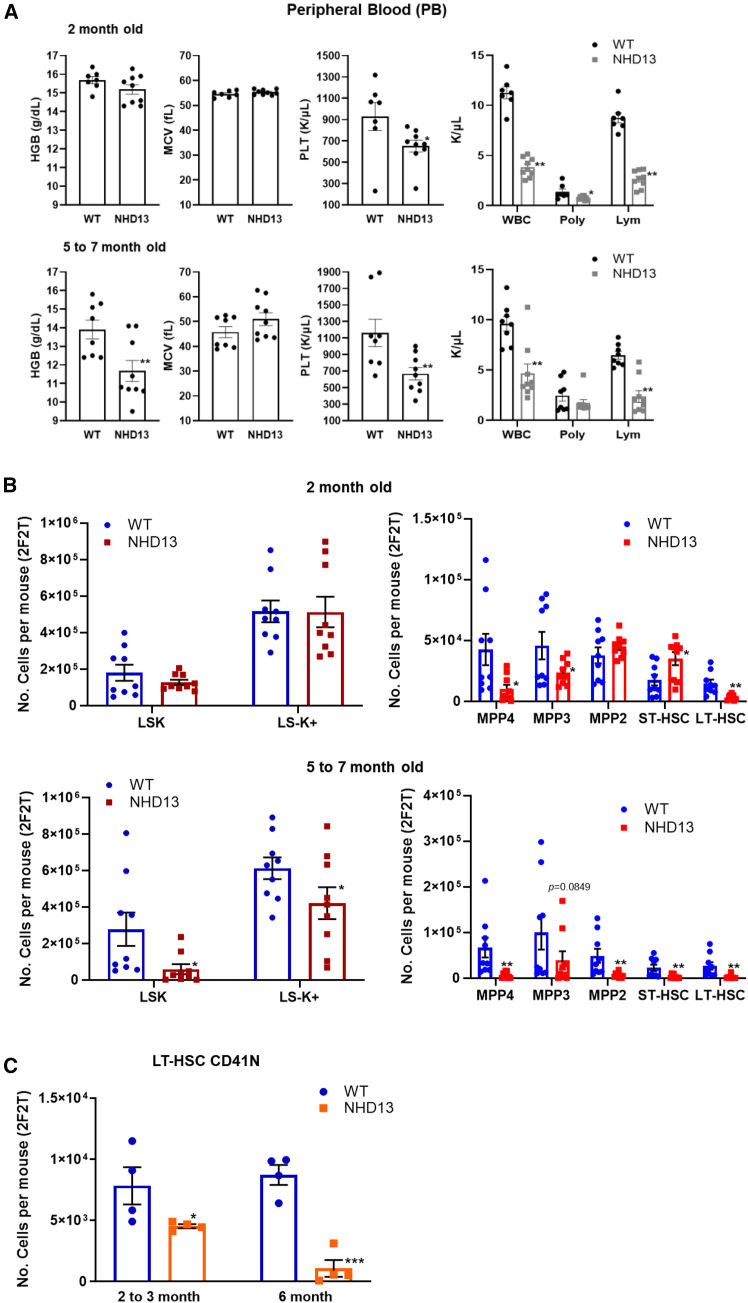
Hematopoietic abnormalities in *NHD13* mice progress with age (A) Complete blood count (CBC) from age-matched *WT* (*n* = 9) and *NHD13* (*n* = 9) mice: ^∗^< *p* = 0.05; ^∗∗^< *p* = 0.01. (B) HSPC analysis from age-matched *WT* (*n* = 9) and *NHD13* (*n* = 9) mice: ^∗^< *p* = 0.05; ^∗∗^< *p* = 0.01. (C) Numbers of CD41 N (negative) LT-HSCs at 2 and 6 months of age: *WT n* = 4, *NHD13 n* = 4; LN, lineage negative; MPP, multi-potential progenitor; 2F2T, two femora and two tibiae per mouse; ^∗^< *p* = 0.05; ^∗∗∗^< *p* = 0.001.

We and others have previously shown that *NHD13* mice invariably develop MDS, with most mice showing anemia and leukopenia by 5–6 months of age (Balderman et al., 2016; Gough et al., 2012; Lin et al., 2005; Nimer, 2008). However, young (2 months old) *NHD13* mice show minimal PB abnormalities, principally, lymphopenia and mild thrombocytopenia and neutropenia (Figure 1A). Anemia (Hgb 11.6 ± 0.5 vs. 13.9 ± 0.5) is the earliest sign of MDS in this model and typically becomes evident in adult (>5 months) *NHD13* mice (Figure 1A). To determine if these age-dependent changes in PB (Gough et al., 2012) indices reflected progressive changes in the hematopoietic stem and progenitor cell (HSPC) compartment with age, we assessed HSPC number in young and adult *NHD13* mice. Two-month-old *NHD13* mice have normal numbers of lineage-negative (Lin^−^), Sca1^+^, Kit^+^ (LSK) and LS^−^K^+^ cells, but further fractionation shows a 4.7-fold decrease in long-term (LT) HSCs and a compensatory increase in short-term (ST) HSCs in the *NHD13* BM. However, consistent with anemia and thrombocytopenia, older (5–7 months) *NHD13* mice show a >30% decrease in LS^−^K^+^ cells, a 4-fold decrease in LSK cells, and a marked decrease in LT- and ST-HSCs compared to age-matched *WT* mice (Figures 1B and S1A). The decreased number of LT-HSC in 2-month-old *NHD13* mice indicates that the defect in early hematopoietic differentiation precedes the most marked PB abnormalities. To assess whether increased proliferation of LSK subsets could compensate for decreased numbers of LSK subsets, we injected mice with bromodeoxyuridine (BrdU) 48 h prior to harvesting BM. There was no consistent difference in the percent of cycling LSK subsets (as determined by BrdU incorporation), except a modest increase in *NHD13* ST-HSCs (Figures S1B and S1C).

CD41 expression on the surface of adult LT-HSCs is dependent on the age and functional potential of HSPCs. CD41^−^ LT-HSCs are thought to reside at the apex of the hematopoietic hierarchy and to be more quiescent and less frequently dividing than CD41^**+**^ LT-HSCs (Bernitz et al., 2016; Gekas and Graf, 2013; Yamamoto et al., 2013). Young *NHD13* mice showed a modest decrease in the absolute number of quiescent CD41^−^ LT-HSCs, and this reduction became more severe in adult *NHD13* mice (Figures 1C and S1D). In contrast, *WT* mice maintained similar numbers of CD41^−^ LT-HSCs in young and adult mice (Figure 1C). Taken together, these results suggest that the MDS phenotype in *NHD13* mice is associated with loss of the most primitive self-renewing HSPCs in the BM.

### HSPCs from *NHD13* mice show decreased self-renewal *in vitro* and *in vivo*

We used functional *in vitro* and *in vivo* assays to identify a potential MDS stem cell within the LSK population. An *in vitro* colony-forming cell (CFC) assay (Ito et al., 2003) was used to assess colony formation of flow-sorted LSK subsets from *NHD13* mice. MPP2 and MPP3 cells produced a decreased number of colonies compared to *WT*, while MPP4 cells, ST-HSCs, and LT-HSCs from *NHD13* mice produced no colonies (Figure S2A). The proportions of burst forming unit erythroid (BFU-E), colony forming unit erythroid (CFU-E), colony forming unit granulocyte macrophage (CFU-GM), colony forming unit megakaryocyte (CFU-Mk), and colony forming unit granulocyte erythroid macrophage megakaryocyte (CFU-GEMM) generated by *NHD13* MPP2 and MPP3 were similar to those of *WT* mice (Figure S2B).

**Figure 2:**
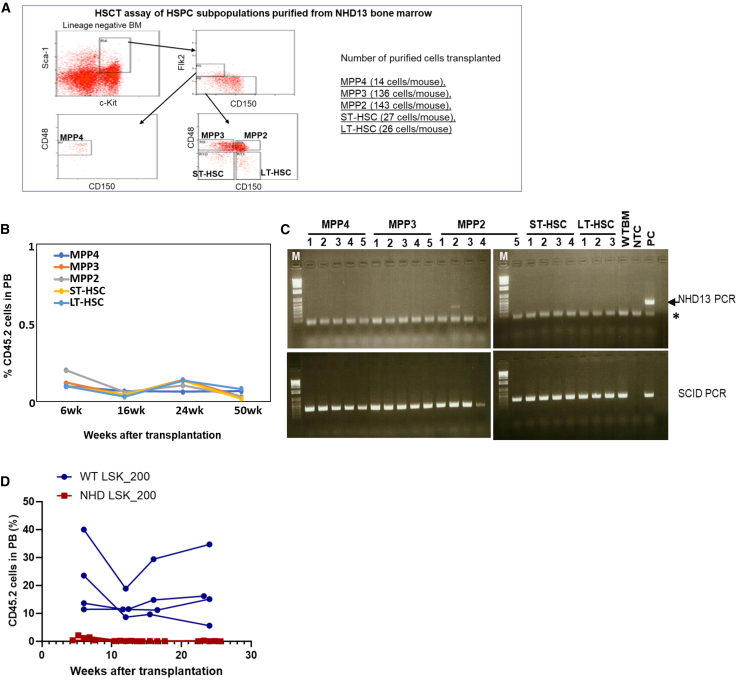
Engraftment of purified HSPC subpopulations from *NHD13* bone marrow (A) Diagram of the experiment and cell sorting. (B) Engraftment of CD45.2^+^*NHD13* cells at indicated time post transplant: MPP4 *n* = 5, MPP3 *n* = 5, MPP2 *n* = 5, ST-HSCs *n* = 4, LT-HSCs *n* = 3. (C) Donor cell engraftment assessed by PCR. Genomic DNA prepared from BM cell aspirated from femur of recipient mice at post-transplantation week 17; PC, positive control for *NHD13* transgene; NTC, no template control; ^∗^ indicates non-specific band, likely primer dimers. The results shown represent one of two independent experiments. (D) Two independent experiments, HSCT of 200 LSK cells from either *WT* or *NHD13* BM: WT LSK_200 *n* = 4, NHD LSK_200 *n* = 5.

We previously reported that MDS was transplantable as a disease entity to healthy recipient mice via HSCT (Chung et al., 2008). Additional experiments by us and others (Balderman et al., 2016; Cheng et al., 2017) have shown that mice transplanted with non-fractionated *NHD13* BM will invariably engraft and that *NHD13* cells will eventually outcompete the *WT* cells (Balderman et al., 2016; Cheng et al., 2017; Chung et al., 2008). In an effort to identify an MDS-initiating cell (MIC), five HSPC subpopulations (MPP2/3/4, ST-HSCs, and LT-HSCs) from young *NHD13* BM (3–4 months old) were purified using flow cytometry and transplanted into lethally irradiated (900 cGy) mice (3–5 recipients each subpopulation) along with 5 × 10^5^ competitor cells (Figure 2A). The *NHD13* cells expressed the CD45.2 isoform, while the competitor cells expressed CD45.1. Serial PB engraftment assays were followed for 1 year (Figures 2B and S2C). Given that one of the hallmarks of MDS is ineffective hematopoiesis, we also searched for *NHD13* cells in the BM of recipient mice at 17 weeks after transplant. With one exception (a faint band in MPP#2), there was no evidence of engraftment of any HSPC subpopulation in the recipients (Figure 2C). A limitation of this experiment is the low number (14–143 cells) of cells transplanted per mouse. However, the results obtained by transplantation of *NHD13* LSK subsets were consistent with those obtained by transplantation of unfractionated *WT* and *NHD13* LSK cells. All (4/4) mice transplanted with 200 *WT* LSK cells engrafted, whereas 0/5 mice transplanted with 200 *NHD13* LSK cells engrafted (Figure 2D). Taken together, these results indicate that *NHD13* HSPC subsets lack effective self-renewal potential and do not successfully engraft recipient mice.

### Committed progenitor cells of *NHD13* MDS mice have self-renewing potential

**Figure 3:**
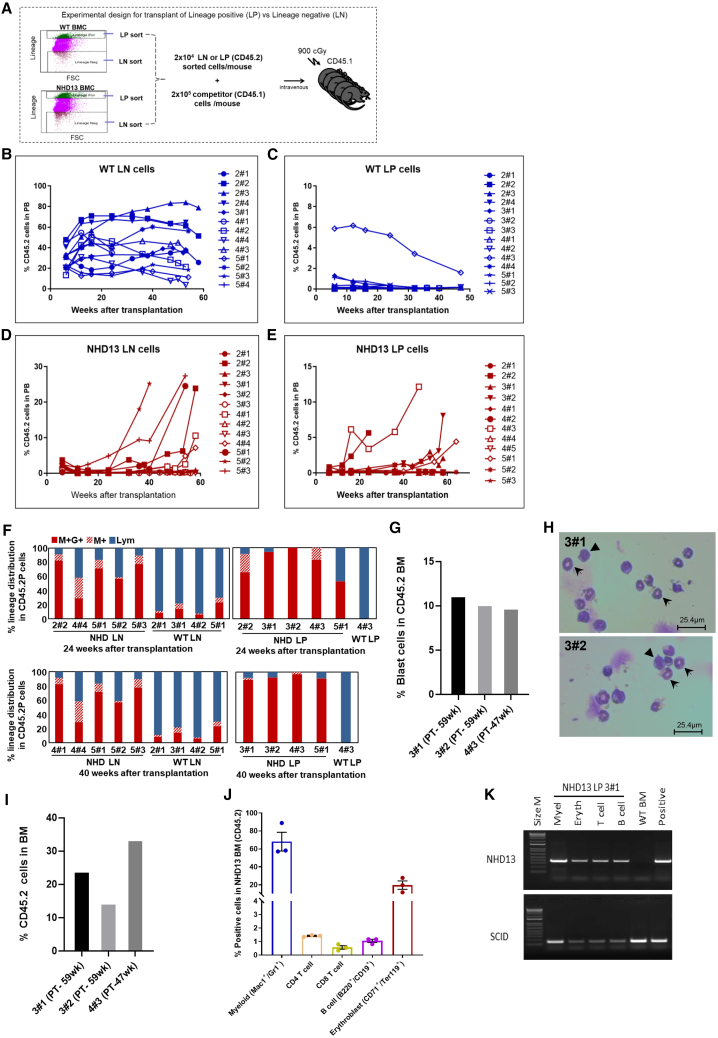
Engraftment and multi-lineage potential of *NHD13* Lin^+^ BM cells (A) Schematic outline of the experiment. Four- to 6-month-old *NHD13* or *WT* mice served as donors. (B–E) Peripheral blood engraftment of Lin^−^ (LN) or Lin^**+**^ (LP) cells from *WT* or *NHD13* donors. Results represent individual mice from four independent experiments (designated by experiment number and mouse number); blue lines indicate engraftment from *WT* LN or LP donor cells. Dark red lines indicate engraftment from *NHD13* LN or LP donor cells. (F) Percentage of myeloid and lymphoid cells at 24 and 40 weeks post transplant (PT): M+, Mac1 single positive; M+ G+, Mac1 and Gr1 double-positive; Lym, lymphoid. (G) Percent blast cells in *NHD13* Lin^**+**^ BM cells at time of euthanasia. (H) Morphology of CD45.2-purified BM cells from *NHD13* LP BM recipients. BM cells were purified using magnetic cell sorting (MACS) with a CD45.2 antibody and stained with May-Giemsa (MG) stain (400X); arrowhead, blast cell; arrow, ring neutrophil. (I) Percent chimerism of *NHD13* cells in *NHD13* LP BM recipients. (J) Summary of differentiated progeny of the *NHD13* Lin^**+**^ BMCs in recipient mice (*n* = 3). (K) Detection of the *NHD13* transgene in flow-sorted populations. Amplification of the *Scid* locus is used as a DNA quality control; decreased intensity of the Scid PCR product is expected for erythroid, T, and B cells as fewer cells were recovered after sorting.

Although it has been well documented that murine WT committed, Lin^+^ BM cells will not self-renew (Spangrude et al., 1988) and that HSPCs lie within the LSK compartment, the lack of engraftment by *NHD13* LSK subpopulations suggested the intriguing possibility that the population responsible for MDS transmission may not lie within the LSK population. Our initial experiments assessed whether the cell type responsible for engraftment and self-renewal might be found in a “committed” Lin^+^ cell population. To limit cross-contamination of cell types, we employed widely separated flow cytometry sorting gates for Lin^+^ and Lin^−^ cells from 3- to 6-month-old *WT* or 4- to 6-month-old *NHD13* mice (Figure 3A; Table S1). As in prior experiments, the *NHD13* cells expressed CD45.2, while the *WT* recipient and competitor cells expressed CD45.1. As expected, *WT* Lin^−^ cells engrafted all recipient mice in all experiments (Figure 3B), while only one mouse engrafted any *WT* Lin^+^ cells (Figure 3C, mouse 4#3). Further investigation demonstrated that this mouse had engrafted only mature CD19^+^B220^+^ B cells, which gradually diminished in number.

6 of 13 mice transplanted with Lin^−^ *NHD13* cells showed evidence of engraftment, typically beginning >20 weeks post transplant (Figure 3D). However, in contrast to the lack of engraftment of *WT* Lin^+^ cells, 5 of 12 mice transplanted with *NHD13* Lin^+^ cells showed LT engraftment (Figure 3E; Table S2), at 20–60 weeks of age, indicating that a self-renewing, Lin^+^ cell had been transplanted. In several cases (3#1, 3#2, 5#1; Figure 3E), there is only low-level engraftment (<1.0%) until 40 weeks post transplant. Transplant of either Lin^−^ or Lin^+^ *NHD13* cells showed a marked skewing toward myeloid cells, which progressed with age (Figure 3F). Comparison of a publicly available gene expression profile of *NHD13* BM and *WT* BM (Novak et al., 2012) using gene set enrichment analysis (GSEA) revealed enrichment of stem cell module gene sets in *NHD13* BM. The GSEA leading-edge analysis showed enhanced expression of *Hoxa5*, *7*, *9*, and *10*; this was confirmed by quantitative reverse-transcription PCR (RT-qPCR) comparison of RNA isolated from *NHD13* Lin^+^ BM cells to RNA isolated from *WT* Lin^+^ BM cells (Figure S3).

To rule out the possibility that the engraftment of *NHD13* Lin^+^ cells represented engraftment of fully transformed AML cells, we euthanized three engrafted mice from this experiment and assessed the percentage of CD45.2 engraftment, and blast counts. Mice euthanized 47–59 weeks post transplant had 24.1% ± 9.6% CD45.2 cells in the BM, less than half of which displayed blast morphology (Figures 3G–3I). The CD45.2 cells were underrepresented in the PB (7.4% ± 5.1%) compared to the BM (24.1% ± 9.6%), consistent with the ineffective hematopoiesis previously reported in the *NHD13* MDS model (Chung et al., 2008). Flow cytometry using lineage commitment markers revealed multilineage hematopoiesis, characterized by the presence of myeloid, erythroid, and lymphoid cells derived from the *NHD13* Lin^+^ cells in the BM (Figures 3J and S4). Cells of *NHD13* origin were identified initially by CD45.2 staining (Figures 3J and S4) and confirmed by PCR amplification of the *NHD13* transgene (Figure 3K).

In addition, despite a median follow-up of 58 weeks (range 24–78) post transplant, none of the recipients showed evidence of transformation to frank AML (Table S3). These results suggest that the impaired engraftment potential of the *NHD13* Lin^−^ cells is compensated by acquisition of engraftment and self-renewal by *NHD13* Lin^+^ cells, supporting the hypothesis that hematopoiesis in older *NHD13* mice is at least partially derived from “committed” Lin^+^ cells.

### Unique immunophenotypic and functional characteristics of *NHD13* LP cells

**Figure 4:**
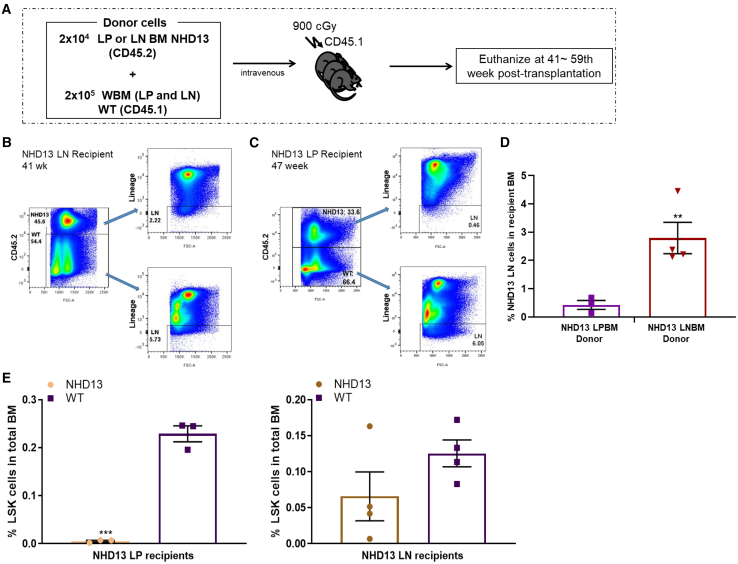
Distinct repopulation patterns from *NHD13* Lin^+^ or Lin^−^ BM cells (A) Schematic of the experiment. (B) Representative flow cytometry profiles from *NHD13* LN recipient. (C) Representative flow cytometry profiles from *NHD13* LP recipient. (D) Percent LN BM cells in LN (*n* = 4) vs. LP (*n* = 3) recipients: ^∗∗^< *p* = 0.01. (E) HSPC repopulation in each transplant group: NHD13 *n* = 3, WT *n* = 3 in *NHD13* LP recipients; NHD13 *n* = 4, WT *n* = 4 in *NHD13* LN recipients; ^∗∗∗^< *p* = 0.001.

We used both immunophenotypic and functional assays to characterize the Lin^−^ and Lin^+^ cells that had demonstrated LT engraftment of *WT* recipients (Figure 4A). We first determined the fraction of Lin^−^ and Lin^+^ cells in recipients that had been transplanted with *NHD13* Lin^−^ (CD45.2^+^) and *WT* BM competitor cells (CD45.1^+^). As shown in Figure 4B, these mice were chimeric, with approximately 50% CD45.2^+^ (*NHD13* derived) and 50% CD45.2^−^ (*WT* derived). Both the CD45.2^+^ (*NHD13*) and CD45.2^−^ (*WT*) cells contained similar percentages of Lin^−^ and Lin^+^ cells. However, although BM from *NHD13* Lin^+^ recipients also showed similar percentages of CD45.2^+^ and CD45.2^−^ cells (Figure 4C), BM derived from *NHD13* Lin^+^ cells (CD45.2^+^) showed a marked decrease in Lin^−^ cells compared to those derived from *WT* BM competitor cells (CD45.2^−^) (Figure 4C, right panel). As shown in Figure 4D, recipients of Lin^−^ *NHD13* cells had 2.98% ± 1.1% Lin^−^ cells, whereas recipients of LP *NHD13* cells had only 0.43% ± 0.3% Lin^−^ cells, a 6.5-fold difference (*p* = 0.00981). These results indicate that the extent of Lin^−^ cell repopulation in *NHD13* BM cells is dependent on the donor cell type, either Lin^−^ or Lin^+^ BM cells.

BM repopulated from *NHD13* Lin^+^ donors showed a markedly diminished ability to generate LSK cells compared with *WT* BM donors (Figure 4E, left panel). In contrast, BM repopulated by *NHD13* Lin^−^ BM showed similar numbers of LSK cells compared to *WT* BM (Figure 4E, right panel). The proportions of *WT* LSK in the recipients of *NHD13* Lin^−^ or Lin^+^ BM were similar, suggesting that there is not a major influence of the *NHD13* cells (Lin^−^ or Lin^+^) on the engraftment and expansion of *WT* BM (Figure 4E). Taken together, these results suggest that *NHD13* Lin^+^ BM has the ability to self-renew and produce Lin^+^ cells directly, as opposed to de-differentiating to *NHD13* Lin^−^ cells, followed by forward differentiation to Lin^+^ cells.

**Figure 5:**
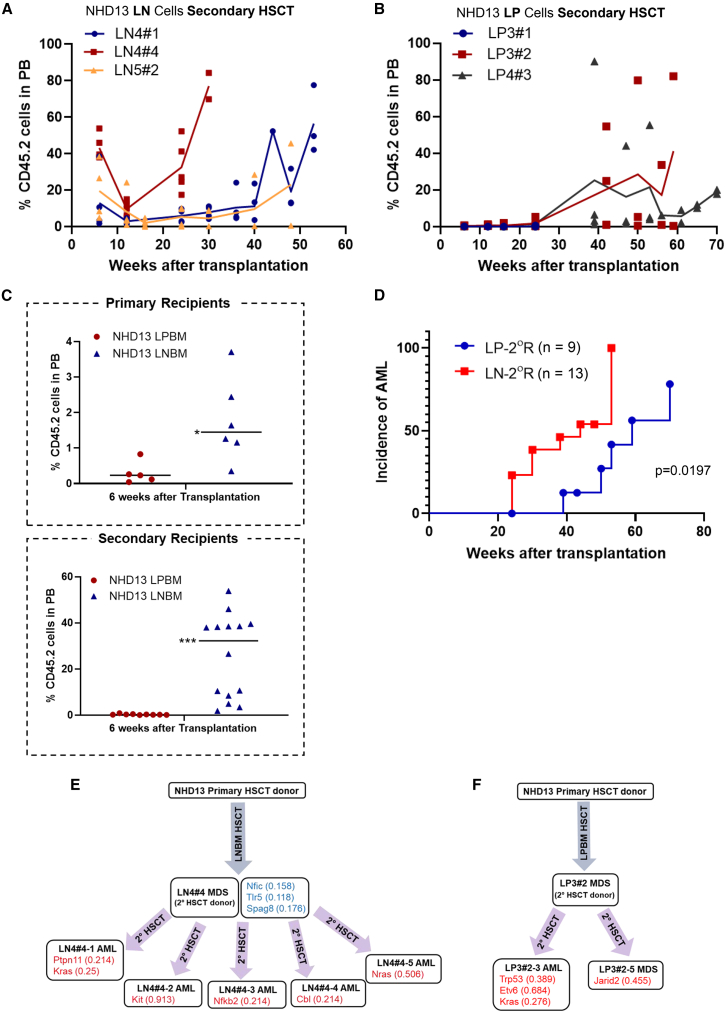
Distinct long-term repopulation features of *NHD13* Lin^+^ vs. Lin^−^ BM cells (A) Secondary recipients of *NHD13* LN BM cells: LN4#1 *n* = 5, LN4#4 *n* = 5, LN5#2 *n* = 4. (B) Secondary recipients of *NHD13* LP BM cells: LP3#1 *n* = 3, LP3#2 *n* = 5, LP4#3 *n* = 4. (C) Short-term (6 weeks) engraftment of primary (upper: LP BM *n* = 5, LN BM *n* = 6) or secondary (lower: LP BM *n* = 9, LN BM *n* = 14) recipients of *NHD13* LN or LP BM cells: ^∗^< *p* = 0.05; ^∗∗∗^< *p* = 0.001. (D) AML incidence in secondary recipients following transplantation. Results from three independent experiments. LP or LN-2° indicates secondary recipients of *NHD13* LP or LN BM, respectively. (E and F) Summary of whole-exome sequencing (WES) results. The digits in the brackets indicate variant allele frequencies.

To determine the *in vivo* functionality of cells derived from *NHD13* Lin^−^ or Lin^+^ cells, we assessed the ability of these cells to engraft recipient mice in a secondary transplant assay. Secondary HSCT recipients received 1 × 10^6^ unfractionated BM cells from primary recipients (Figures 3D and 3E). Since the primary recipients were chimeric, the total number of *NHD13* cells transplanted varied between 1.4 and 5.2 × 10^5^ cells (Table S3). The secondary HSCT demonstrated that the *NHD13* Lin^−^ BM cells successfully engrafted most recipients, with the majority of engrafted recipients eventually transforming to AML (Figure 5A; Table S4). Similar results were seen with the secondary HSCT from the *NHD13* Lin^+^ donors (Figure 5B; Table S4). Of note, secondary recipients of *NHD13* Lin^−^ BM showed an engraftment pattern that was dependent on the primary donor. For instance, recipients of 4#4 BM (red squares) showed a high engraftment at 6 weeks post transplant (43.1% ± 3.1%), followed by a nadir of 9.66% ± 1.8% engraftment at 12 weeks, culminating with a terminal increase to 32.5%–77.0% at 24–30 weeks post transplant (Figure 5A). Recipients of 4#1 and 5#2 BM (blue circles, yellow diamonds) showed modest initial engraftment (from week 6–30), with a terminal increase in weeks 40–52. In contrast, secondary recipients of *NHD13* Lin^+^ BM all showed a pattern that was independent of the primary donor, with minimal initial engraftment (<2%) in all recipients up to 12 weeks, followed by a highly variable terminal engraftment at weeks 30–70 (Figure 5B).

Primary recipients of *NHD13* Lin^−^ BM showed increased engraftment compared to Lin^+^ BM recipients (Figure 5C, upper panel). However, the secondary recipients of Lin^−^ BM showed a more dramatic difference in the rapidly engrafting population (Figure 5C, lower panel). Although most mice in both the Lin^−^ and Lin^+^ secondary HSCT groups developed AML, the Lin^−^ recipients developed AML more rapidly (median 44 vs. 59 weeks; Figure 5D; Table S4). Taken together, these secondary transplant results suggest that a pre-leukemic, but not fully transformed, self-renewing population exists within the Lin^+^ compartment.

We used whole-exome sequencing (WES) to identify acquired mutations in MDS and AML samples from primary and secondary transplants. Comparison of AML or MDS tissue to normal tissue from the same individual was of limited utility in identifying acquired mutations, since the leukemias were derived from donor mice (NIH C57BL/6 *NHD13* CD45.2 breeding colony) and the normal tissues were from recipient mice (Charles River C57BL/6 CD45.1). Therefore, we compared the AML/MDS samples to pooled tail DNA (*n* = 3) from the *NHD13* breeding colony, using Mutect or Dragen software and recommended criteria (BaitRegion = TRUE, FILTER = PASS, Impact = MODERATE or HIGH, variant allele frequency [VAF] > 0.2). We eliminated any identical single-nucleotide variants (SNVs) that were present in mice from different experiments (using different donors), as previous studies have shown that these are most likely rare germline SNVs that were present in the breeding colony as opposed to acquired oncogenic SNVs (Goldberg et al., 2017). We next eliminated any SNVs that were also identified in *WT* tissue from recipient mice as these were due to contaminating *WT* tissue in the tumor sample. We were left with 5–12 acquired tier I SNVs per sample (Table S5).

The LN4#4 sample (used as donor for the secondary transplant) had acquired a prominent, but not dominant, clone characterized by tier I variants in *Tlr5, Nfic*, and *Spag8*, with a VAF of 0.12–0.18. These variants, as well as a variant in the *Tigit* gene, are present in all seven samples from the recipient mice with a mean VAF of 0.48 ± 0.08, suggesting that this clone had a fitness advantage *in vivo*. All samples had acquired at least one additional mutation in signaling pathway genes that are well known to be involved in AML, including *Ptpn11*, *Kras*, *Nras*, *Kit*, *Cbl*, and *Nfkb2* (Figure 5E). Of note, none of the acquired mutations were the same between different recipients, indicating significant diversity in collaborating events that occurred as the disease evolved from MDS to AML. However, acquired oncogenic mutations in the flow-sorted subsets (Figure S4B) from LN4#4-1 and LN4#4-5 were identical (Table S5), leading us to suspect that the difference in antigen expression (Mac1/Gr1 vs. Kit) in those mice was due to epigenetic, as opposed to genetic, events. In contrast to the findings with LN4#4 recipients, the two recipients of BM from donor LP3#2 had clearly distinct clones, one with oncogenic mutations involving *Trp53*, *Etv6*, and *Kras* and the other with a *Jarid2* frameshift mutation (Table S5). Of note, the recipient with a signaling mutation (*Kras*) evolved to AML, whereas the recipient with an epigenetic mutation (*Jarid2*) died of MDS without transformation to AML (Figure 5F). Taken together, these results demonstrate that *NHD13* LP cells can persist and self-renew for an extended period of time (27 ± 3 months; Table S6) before ultimately transforming to AML, following acquiring mutations in genes well known to be relevant for human MDS/AML (Makishima et al., 2017; Sperling et al., 2017).

### MICs are enriched in a B220^+^/Kit^+^ population

**Figure 6:**
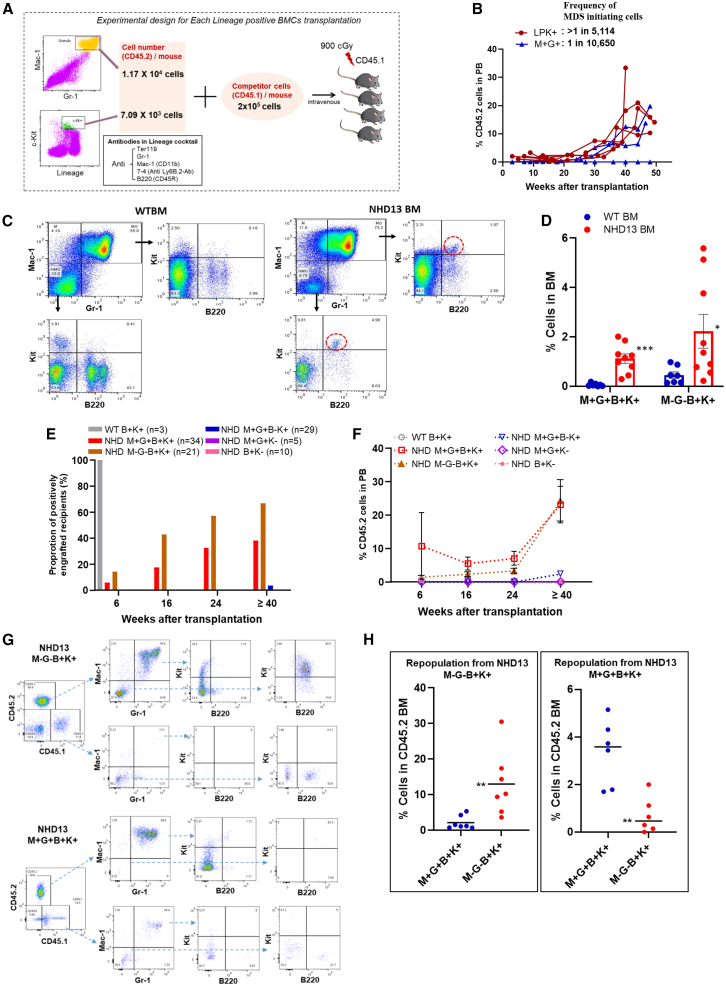
MDS-initiating cell populations in *NHD13* Lin^+^ BM (A) Outline of the experiment. (B) Engraftment kinetics and estimated frequency of MIC in each sorted cell population. LPK+, positive for lineage makers and Kit (*n* = 4); M+ G+, positive for both Mac1 and Gr1 (*n* = 3). (C) Comparison of Mac1, Gr1, B220, and Kit staining of *WT* and *NHD13* BM cells. (D) Proportion of M+G+B+K+ (Mac1^+^Gr1^+^B220^+^Kit^+^) and M−G−B+K+ (Mac1^−^Gr1^−^B220^+^Kit^+^) cells in *NHD13* (*n* = 9) and *WT* BM (*n* = 7): ^∗^< *p* = 0.05; ^∗∗∗^< *p* = 0.001. (E) Each bar represents the proportion of recipient mice engrafted with CD45.2 cells (≥0.5%) in PB at the indicated time. Results analysis from seven independent experiments. (F) Mean percent engraftment of engrafted recipients at the indicated time. Each number of recipients is the same as indicated in the graph (E) legend. Results from seven independent experiments. (G) Representative flow cytometry profiles for recipients of *NHD13* M−G−B+K and M+G+B+K+ cells. (H) Summary of repopulation from *NHD13*: M+G+B+K+ *n* = 7, M−G−B+K+ *n* = 7 in the left; M+G+B+K+ *n* = 6, M−G−B+K+ *n* = 6 in the right; ^∗∗^< *p* = 0.01.

Given that the Lin^+^ cells utilized in the aforementioned experiments consist of a heterogeneous population of cells, we fractionated Lin^+^ cells using cell surface markers that we thought might contain a self-renewing MIC. Because the self-renewing MICs were myeloid biased, we tested Mac1^+^Gr1^+^ cells, and because Kit expression is often associated with stem cell self-renewal (Rojas-Sutterlin et al., 2014), we tested Kit^+^ cells. Flow-sorted Mac1^+^Gr1^+^ and Lin^+^Kit^+^ cells isolated from *NHD13* BM were transplanted along with *WT* competitor cells, as outlined in Figure 6A (Table S1). Both the Mac1^+^Gr1^+^ and Lin^+^Kit^+^ populations were able to self-renew and engraft; however, all (4/4) mice transplanted with Lin^+^Kit^+^ cells engrafted, whereas 2 of 3 mice transplanted with the Mac1^+^Gr1^+^ population engrafted (Figure 6B; Table 1). The pattern of engraftment (Figure 6B) was similar to that seen with *NHD13* Lin^+^ cells shown in Figure 3E, with low level engraftment until week 30, followed by a brisk expansion, terminating in a lethal MDS or AML by week 50.

**Table 1:** Frequency of an MDS-initiating cell among lineage-positive BM cells

*NHD13* BM	Exp.	Cell dose for testing	No. of responders	No. of test	Estimated transplantable cell frequency	95% confidence interval
M+G+	LP	1.17 × 10^4^	2	3	1/10,650	1/45,706–1/2,481
LPK+	LP	7.09 × 10^3^	4	4	>1/5,114	1/17,400–1/1,503
M+G+B+K+	A	1,000	4	5	1/1,110	1/1,942–1/634
	B	1,000	1	4		
	C	597	2	5		
		199	3	5		
	D	200	0	10		
	E	1,000	3	5		
M−G−B+K+	A	3,500	2	2	1/899	1/1,607–1/502
	B	1,000	0	5		
	D	1,500	4	4		
		500	3	5		
	E	1,000	5	5		

M+, Mac1 positive; G+, Gr1 positive; M−, Mac1 negative; G−, Gr1 negative; B+, B220 positive; K+, Kit positive; LP, lineage positive. Exp. indicates the designation for independent experiments.

B220^+^Kit^+^ cells have previously been reported to function as progenitors with lymphoid and myeloid differentiation potential in *WT* mice (Balciunaite et al., 2005; Kawaguchi, 2005; Ogawa et al., 2000). Given that self-renewal potential had been identified in Mac1^+^Gr1^+^ cells as well as Lin^+^Kit^+^ cells, we assessed the presence of B220^+^Kit^+^ cells within both the Mac1^+^Gr1^+^ and Mac1^−^ Gr1^−^ populations. Markedly increased numbers of B220^+^Kit^+^ cells were identified in *NHD13* BM compared to *WT* BM, among both the Mac1^+^Gr1^+^ and Mac1^−^ Gr1^−^ populations (Figures 6C and 6D). In order to assess the function of these *NHD13* populations (Mac1^−^ Gr1^−^ B220^+^Kit^+^ and Mac1^+^Gr1^+^ B220^+^Kit^+^) from 5-month-old mice (median age; Table S1), we tested the ability of these populations to engraft *WT* mice. Mac1^+^Gr1^+^ B220^−^ Kit^+^, Mac1^+^Gr1^+^ Kit^−^, and B220^+^Kit^−^ cells from *NHD13* BM and B220^+^Kit^+^ cells from *WT* BM were used as additional controls (Figures 6E, 6F, and S5). Pooled engraftment results from seven independent experiments are summarized in Table S7. *WT* B220^+^Kit^+^ cells showed positive engraftment (≥0.5% CD45.2 cells in PB) at post-transplant week 6 but no LT repopulating potential (Figures 6E, 6F, and S5). However, *NHD13* B220^+^Kit^+^ BM cells showed repopulation ability in both the Mac1^+^Gr1^+^ or Mac1^−^ Gr1^−^ recipient groups (Figures 6E, 6F, and S5), summarized in Table S7. Additionally, the *NHD13* Mac1^−^ Gr1^−^ B220^+^Kit^+^ cells repopulate and produce both Mac1^+^Gr1^+^ B220^+^Kit^+^ and Mac1^−^ Gr1^−^ B220^+^Kit^+^ cells in recipient mice along with multilineage potential, whereas Mac1^+^Gr1^+^ B220^+^Kit^+^ cells repopulate primarily with Mac1^+^Gr1^+^ B220^+^Kit^+^ cells and very few Mac1^−^ Gr1^−^ B220^+^Kit^+^ cells with lack of multilineage potential, suggesting that Mac1^−^ Gr1^−^ B220^+^Kit^+^ cells may be more immature than Mac1^+^Gr1^+^ B220^+^Kit^+^ cells (Figures 6G, 6H, S6A, and S6B). Taken together, these results reveal that an MIC is present in *NHD13* B220^+^Kit^+^ cells and that Mac1^+^Gr1^+^ B220^+^Kit^+^ cells from *NHD13* mice can self-renew for at least 56 weeks (Table S8).

We used WES to identify acquired mutations in MDS or AML that developed in mice transplanted with flow-sorted subpopulations (Table S9). Although minor clones within the donor population evolved into major clones in the recipients of sorted cells from 5205 to 5213 (marked by *Btbd18*/*Rapgef1* and *Ptk7*/*Tmem266* mutations, respectively), there were no common mutations in the recipients from donor 5141 or 5166.

Remarkably, 4 of 5 recipients of 5141 BM and 2 of 6 recipients of 5213 BM had acquired at least one independent truncation mutation of *Mn1* (Table S9), suggesting *in vivo* selection of clones that had acquired *Mn1* truncation mutations. All *Mn1* mutations encoded truncated versions of *Mn1*; there were no missense mutations. Moreover, 4 of the 7 independent *Mn1* mutations were clustered within a 6 bp “hotspot” (Table S9; Figure S6C). We identified two additional Mn1 truncation mutations that were associated with murine AML in the literature (Nofrini et al., 2011; Slape et al., 2007), both near amino acid (aa) 1240 of MN1. Including these two mutations, all nine of the MN1-truncating mutations retain the first 838 aa but the variations occur in the rest of 459 aa region (Figure S6C), suggesting an oncogenic effect of the truncated MN1. All these mutation analyses suggest that MICs may need to have additional relevant mutations for developing AML.

## Discussion

The incidence of MDS has long been known to increase with age, showing an exponential increase starting at age 60 (Maynadie et al., 1996). In this study, we show that this age-dependent disease can be recapitulated using the *NHD13* mouse model for MDS. Although young *NHD13* mice appear healthy, with normal PB counts and indices at 2 months of age, these mice invariably develop pancytopenia by 7 months of age. This decrease in hematopoietic output is reflected in the HSPC compartment; at 2 months of age, there was no difference in LSK number between *WT* and *NHD13* mice, while at 7 months of age, there was a 4.5-fold decrease in LSK cells in *NHD13* mice. Furthermore, *NHD13* BM showed a marked decrease in CD41^−^ LT-HSCs, a population which is thought to reside at the apex of the HSC hierarchy (Bernitz et al., 2016; Gekas and Graf, 2013; Yamamoto et al., 2013).

Despite the clear loss of LSK and HSPC numbers, *NHD13* mice maintain PB complete blood counts (CBCs) that are only modestly decreased, and mice are generally asymptomatic until 8+ months of age. We considered the possibility that increased function of the *NHD13* HSPCs could theoretically serve to maintain PB counts. However, CFC and HSCT assays indicated that *NHD13* HSPCs had decreased, as opposed to increased, functional output.

We finally considered the possibility that hematopoiesis in *NHD13* mice may be driven by Lin^**+**^, as opposed to the expected Lin^−^ cell population. In support of this hypothesis, *NHD13* Lin^+^ cells express a stem cell module, including genes well known to be overexpressed in stem and progenitor cells, such as Hoxa7, Hoxa9, and Meis1. An *in vivo* HSCT assay supported this possibility, as approximately half of the mice transplanted with *NHD13* Lin^+^ cells showed LT multilineage engraftment, whereas none of the mice transplanted with *WT* Lin^+^ cells showed LT multilineage engraftment. In this context, it is important to note that chromatin immunoprecipitation experiments have shown that the NHD13 protein is bound to important upregulated target genes, such as *Hoxa*, *Hoxb*, and *Meis1* (Xu et al., 2016). This leads us to speculate that enforced expression of an NHD13 protein, driven by *Vav1* regulatory elements, enforces a self-renewal program in NHD13-committed progenitor cells.

The surprising finding that MDS could be transplanted by a Lin^+^ cell led us to further refine the nature of an MIC. Recipients of Lin^+^ BM cells produced very few LSK cells compared to recipients of Lin^−^ BM, indicating that engraftment of the Lin^+^ cells was not due to transplantation of contaminating Lin^−^ cells, nor was it due to de-differentiation of Lin^+^ cells to a Lin^−^ population. In addition, secondary transplant experiments indicated that MDS could be transmitted by both Lin^−^ and Lin^+^ cells but that the pattern of engraftment differed between these two populations. Whereas engraftment of Lin^−^ cells showed a rapid burst of engraftment, followed by a rapid decline and a subsequent terminal rise, engraftment of Lin^+^ cells showed no rapid engraftment phase but instead an extended period (>26 weeks) of quiescence, followed by an eventual terminal rise in engraftment.

An MIC could be further refined based on the expression of the B220 and Kit cell surface markers. Over half of mice transplanted with B220^+^Kit^+^ cells showed LT engraftment, whereas <5% of mice transplanted with B220^−^Kit^+^ or B220^+^Kit^−^ cells showed LT engraftment. Of note, the frequency of B220^+^Kit^+^ cells was markedly increased in the BM of *NHD13* mice, which inevitably develop MDS (Lin et al., 2005; Slape et al., 2008), compared to *WT* mice. The B220^+^Kit^+^ cells were further fractionated using Mac1 and Gr1 surface markers using a limiting dilution assay. These experiments demonstrated that the estimated transplantable cell frequency of both Mac1^+^Gr1^+^ B220^+^Kit^+^ and Mac1^−^ Gr1^−^ B220^+^Kit^+^ cell populations was ∼1/1,000, demonstrating that Mac1^+^Gr1^+^ cells, which are typically regarded as terminally differentiated, can self-renew and transmit MDS. These results are not entirely unexpected, as a prior study demonstrated that Mac1^+^B220^+^ BM cells transduced with a PICALM::AF10 expression vector could self-renew as AML cells (Deshpande et al., 2006). These results, based on the *NHD13* mouse model, support recent findings that heavily mutated HSPCs from human patients with MDS retain the ability to both self-renew and differentiate into a spectrum of mature hematopoietic cell types (Rao, 2023; Schnegg-Kaufmann et al., 2023). In that study, the maturation of mutant HSPCs was more prominent following treatment with a DNA methyltransferase inhibitor, in at least a subset of patients (Schnegg-Kaufmann et al., 2023).

WES of AML or MDS cells from primary or secondary transplant experiments revealed several patterns of acquired mutations. In one example, a low VAF founder clone present in the primary transplant recipient became the predominant clone in AML that evolved in the secondary recipients. Of note, the secondary recipients all had unique mutations in genes encoding signaling proteins that are well known to be mutated in human AML (*Ptpn11*, *Nras*, *Kras*, *Cbl*, and *Kit*). These results, which show acquisition of mutations in signaling proteins as the disease evolves from MDS to AML, are similar to findings in human patients (Makishima et al., 2017; Sperling et al., 2017). An additional feature seen in a separate experiment was the acquisition of six different Mn1 mutations in recipients from two independent donors. All *Mn1* mutations encoded truncated versions of Mn1, suggesting that there was a strong *in vivo* selection for Mn1 mutations in the context of MDS caused by an *NHD13* transgene.

In summary, this study demonstrates that MDS progression in the *NHD13* mouse model is associated with a marked decrease in primitive HSPCs. This vacuum of normal hematopoiesis is then partially replaced by abnormal hematopoiesis originating from a multipotential B220^+^ Kit^+^ MIC. These data provide new insight into the pathogenesis and evolution of MDS and provide a foundation for the development of future therapeutic strategies.

## Methods

### Mice

All mice for this study were generated and maintained on a C57BL/6 background. The *NHD13* transgenic mice were bred and maintained in NIH animal facilities. Two- or 7-month-old *NHD13* mice (CD45.2) were used for MDS hematopoiesis analysis. Eight- to 10-week-old C57BL/6 (CD45.1) recipient mice that were used for HSCT were purchased from either Charles River or Jackson Laboratory and maintained under micro-isolation conditions. All mouse handling and procedures were approved by the NCI Bethesda Animal Care and Use Committee (ACUC).

### Flow cytometry

Antibodies for flow cytometry were obtained from either BD Pharmingen (BD), Thermo Fisher Scientific (TFS), BioLegend (BL), or SouthernBiotech (SB). Staining was performed in Hank’s balanced salt solution (Ca^2+^, Mg^2+^ free, Invitrogen, CA) containing 2% fetal bovine serum (FBS) for 30–60 min at 4°C. Combinations of antibodies and instruments used were as follows. For HSPC analysis, Lineage Biotin Antibody Cocktail (Miltenyi Biotec), Streptavidin-PerCP-Cy5.5 (TFS), CD117 (cKit)-APC-eFluor 780 (TFS), Sca-1-PE-Cy7 (TFS), CD150-APC (BL), CD135 (Flk2)-BV421 (BD), CD48-FITC (TFS), and DAPI (BD) were analyzed with a LSRFortessa instrument (BD Biosciences). For the evaluation of engraftment, Mac-1-PE (TFS), Gr-1-FITC (TFS), CD45.2-APC (BL), B220-PerCP-eFlour 710 (TFS), CD4-PerCP-Cy5.5 (BL), CD8-APC-Cy5.5 (SB), CD16/32 (clone 2.4G2, BD), and 7AAD were analyzed with a Northern Lights instrument (Cytek Biosciences). For committed lineage cell analysis, Mac-1-PE (TFS), Gr-1-FITC (TFS), CD71-PE/Dazzle594 (BL), B220-PerCP-eFlour 710 (TFS), CD19-Alexa Flour 700 (BL), CD4-PerCP-Cy5.5 (BL), CD8-APC-Cy5.5 (SB), Ter119-APC (BL), CD117 (cKit)-APC-eFluor 780 (TFS), and Sca-1-PE-Cy7 (TFS) were analyzed. CD45.1-Alexa Fluor 532 (TFS), CD45.2-PerCP (BL), and 7AAD were analyzed with a Northern Lights instrument (Cytek Biosciences).

### Cell proliferation assay with BrdU

For cell proliferation assessment, mice were injected with 1 mg of BrdU intraperitoneally 48 h prior to euthanasia and BM harvest. BrdU and HSPC staining were performed using the manufacturer’s suggested protocol and reagents (BrdU Flow Kit; BD Pharmingen).

### HSCT procedure and engraftment evaluation

HSCT and engraftment assays were performed with minor modification of previously published procedures (Chung et al., 2018). In brief, BM cells (BMCs) from femora and tibiae of mice were stained with the antibody combinations as outlined in the text, and then collected using a MoFlo Astrios EQ (Beckman Coulter, IN) cell sorter. 1 × 10^3^ to 2 × 10^4^ sorted *NHD13* BMCs (CD45.2) were injected into lethally irradiated (900 cGy) WT recipient mice (CD45.1) via tail vein along with 2 × 10^5^ BMCs from a non-irradiated healthy WT donor (CD45.1, used as competitor cells). For secondary transplantation, 1 × 10^6^ of primary recipient BMCs per mouse were transplanted to lethally irradiated recipients.

Engraftment assays were carried out with PB obtained from the tail vein using di-potassium EDTA (ethylenediaminetetraacetic acid) salt as an anticoagulant. Each PB sample was separated into samples for flow cytometry and CBCs. CBCs were determined using a HEMAVET Multispecies Hematology Analyzer (CDC Technologies, Oxford, CT). To evaluate engraftment level in tissues other than PB (typically BM or spleen), mice were euthanized, and tissues were harvested and placed into a single-cell suspension in Hank’s balanced salt solution supplemented with 2% FBS (HF2) buffer.

Morphology of PB and BM was evaluated using May-Grünwald Giemsa staining of air-dried PB smear slides or BMC cytospin slides.

### DNA extraction and PCR

Genomic DNA was extracted from PB or BMCs using the DNeasy Blood & Tissue kit (QIAGEN) and the manufacturer’s suggested protocol. The *NHD13* transgene was amplified using primers 5′-TGGAGGGCCTCTTGGTACAGG-3′ (NUP98001) and 5′- GGCTTCTAAGCTGTCTGTGGCC-3′ (HOXD13-L1), and the program consisted of incubation at 95°C for 3 min, followed by 35 cycles of 95°C for 30 s, 62°C for 30 s, and 72°C for 30 s. Scid gene, as DNA quality control, was amplified using primers 5′-GGAAGAGTTTTGAGCAGACAATG-3′ (SCID A) and 5′- CATCACAAGTTATAACAGCTGGG-3′ (SCID B), and the program consisted of incubation at 95°C for 3 min, followed by 35 cycles of 95°C for 30 s, 56°C for 30 s, and 72°C for 30 s.

### GSEA and RT-qPCR

GSEA was performed with previously reported gene expression data (Novak et al., 2012) using the GSEA software (Subramanian et al., 2005). For RT-qPCR verification, complementary DNAs were synthesized using Superscript III Reverse Transcriptase (Invitrogen) with RNAs isolated by Trizol (Invitrogen) and the manufacture’s protocol. RT-qPCR analysis was performed on a 7500 Fast RT-PCR system (Applied Biosystems, CA, USA) using the default thermal cycling conditions. 18S ribosomal RNA was used as an internal control. TaqMan primer and probe sets (Applied Biosystems) were used for Hoxa5 (Mm00439362_m1), Hoxa7 (Mm00657963_m1), Hoxa9 (Mm00439364), and Hoxa10 (Mm00433966).

### CFC

CFCs were plated with 50–100 cells onto 35-mm Petri dishes in Methocult M3434 methylcellulose medium (STEMCELL Technologies, Canada) supplemented with cytokines (50 ng/mL recombinant mouse stem cell factor, 10 ng/mL rmIL-3, 10 ng/mL rhIL-6, and 3 U/mL rhEpo) and were incubated at 37°C in a 5% CO_2_ incubator. The number of colonies was counted at day 10 after plating the cells.

### WES capture and mutation analysis

Mouse Illumina DNA libraries were prepared and captured using the Agilent SureSelectXT Mouse All Exon Kit according to the manufacturer’s instructions. In brief, DNA was fragmented on a Covaris S1 sonicator followed by end repair and phosphorylation. Blunt fragments were adenylated, ligated to Illumina Y-adapters, and PCR amplified. Bait hybridization proceeded for 48 h, followed by recovery of captured exome fragments by PCR. Captured exomes were sequenced on an Illumina HiSeq 2000.

Data processing and variant calling procedure mainly followed the Best Practices workflow recommended by the Broad Institute (http://www.broadinstitute.org/gatk/guide/best-practices). Briefly, the raw sequencing reads were mapped to mouse genome build 10 (mm10) by the Burrows-Wheeler Aligner (Li and Durbin, 2009), followed by local realignment using the GATK suite from Broad Institute, and duplicated reads were marked by Picard tools (http://picard.sourceforge.net).

Somatic variant calling was performed on data of tumor/normal paired samples by Mutect2 in GATK suit (https://gatk.broadinstitute.org/hc/en-us/articles/360037593851-Mutect2), and germline variant calling was done with the UnifiedGenotyper from the Broad Institute (https://www.broadinstitute.org/gatk/). SnpEff (Cingolani et al., 2012) and dbSNP 137 (NCBI) were used to annotate and predict effects of the variants.

The following filtering criteria were used for germline variation calls: (1) minimum read depth is 5; (2) minimum altered read number is 3; (3) minimum fraction of altered reads is 0.01; and (4) impact is “high” or “moderate.”

The following filtering criteria were used for somatic variation calls: (1) minimum read depth is 5; (2) maximum altered read number in reference samples (“normal”) is 2, or maximum fraction of altered reads in reference samples (normal) is 0.01; (3) minimum altered read number in samples with the fusion genes is 3 or minimum fraction of altered reads in tumor samples is 0.1; and (4) impact effect is high or moderate.

### Statistics

Data are expressed as the mean ± standard errors of the mean or standard deviation where applicable. Differences between groups were analyzed by Student’s t test.

## Resource availability

### Lead contact

All other information and request for resources and reagents should be directed to the lead contact, Peter D. Aplan (aplanp@mail.nih.gov).

### Materials availability

All materials used in this study are available from commercial vendors or from the authors.

### Data and code availability

WES data are available in the sequence read archive under accession number, SRA BioProject ID: PRJNA1252163 (http://www.ncbi.nlm.nih.gov/bioproject/1252163) and PRJNA1256132 (http://www.ncbi.nlm.nih.gov/bioproject/1256132).

## Acknowledgments

The authors thank current and former members of the Aplan lab. We thank the NCI Sequencing Minicore for Sanger sequencing, the NCI Transgenic Core for generation of transgenic mice, the NCI Genomics Core for Next Generation Sequencing, the NCI Flow cytometry core for cell sorting, the NCI Pathology/Histotechnology Lab (PHL) for immunohistochemistry, and Maria Jorge for excellent animal husbandry. This work was supported by the 10.13039/100030692Intramural Research Program of the 10.13039/100000054National Cancer Institute, National Institutes of Health (grant nos. ZIA SC 010378 and BC 010983).

## Author contributions

Y.J.C. designed and performed research, analyzed the data, and wrote the first draft of the manuscript; R.B. performed the experiments and analyzed the data; D.C. participated in the design of the study and data analysis; R.L.W. performed data curation, formal analysis, and investigation; Y.J.Z. performed data curation, formal analysis, validation, and visualization; P.M. designed research and analyzed the data; and P.D.A. designed research, analyzed the data, and wrote the final draft of the manuscript.

## Declaration of interests

P.D.A. receives royalties from the NIH Technology Transfer program for the invention of *NHD13* mice.

## Supplemental information

Document S1. Figures S1–S6 and Tables S1–S9

Document S2. Article plus supplemental information
