# Supplementary material for: Disruption of normal stem cell function and transmission of myelodysplastic syndrome by self-renewal of committed myeloid lineage cells
Source: Stem Cell Reports. 2025 Jul 3;20(8):102571. doi: 10.1016/j.stemcr.2025.102571 (PMC12365822; doi:10.1016/j.stemcr.2025.102571)
Supplement: Document S1. Figures S1–S6 and Tables S1–S9 [file mmc1.pdf]

Stem Cell Reports, Volume *20*

Supplemental Information

Disruption of normal stem cell function and transmission of myelodysplastic syndrome by self-renewal of committed myeloid lineage cells

Yang Jo Chung, Ryan Bertoli, Dengchao Cao, Robert L. Walker, Yuelin Jack Zhu, Paul Meltzer, and Peter D. Aplan

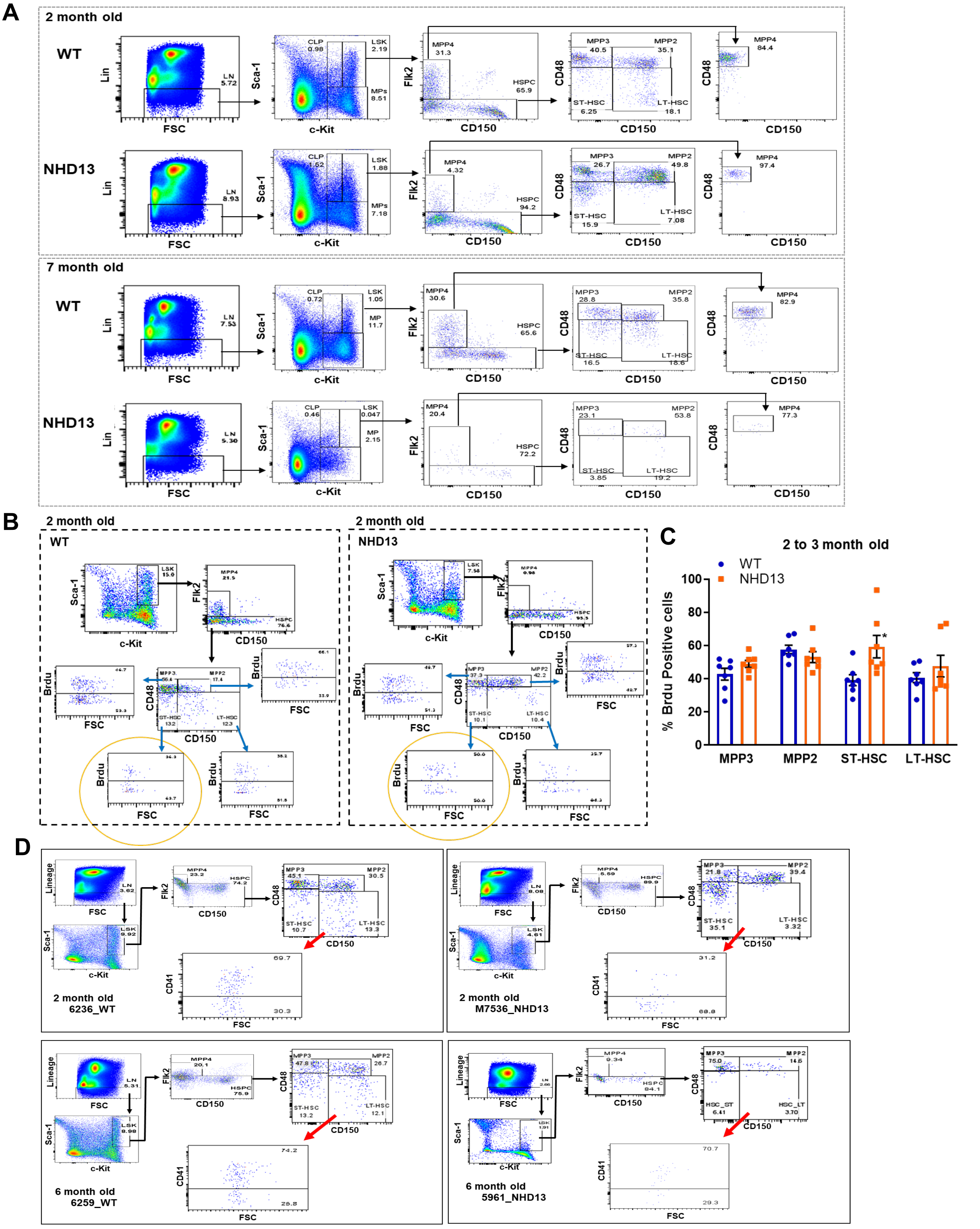

Supplementary figure S1. Hematopoietic stem and progenitor cell populations and proliferation in WT and NHD13 vary with age. (A) Representative flow cytometry profiles of BM HSPCs from age matched WT and NHD13 mice. **(B)** Representative flow cytometry profiles used to assess proliferation of HSPC in young (2-3 month) NHD13 bone marrow using Brdu incorporation. **(C)** Cell proliferation comparison of each LSK subset. Sample size for WT n=7, and for NHD13, n=7: * < p=0.05. **(D)** Representative flow cytometry profiles of HSCs on CD41 antigen expression from both genotype mice with differential age.

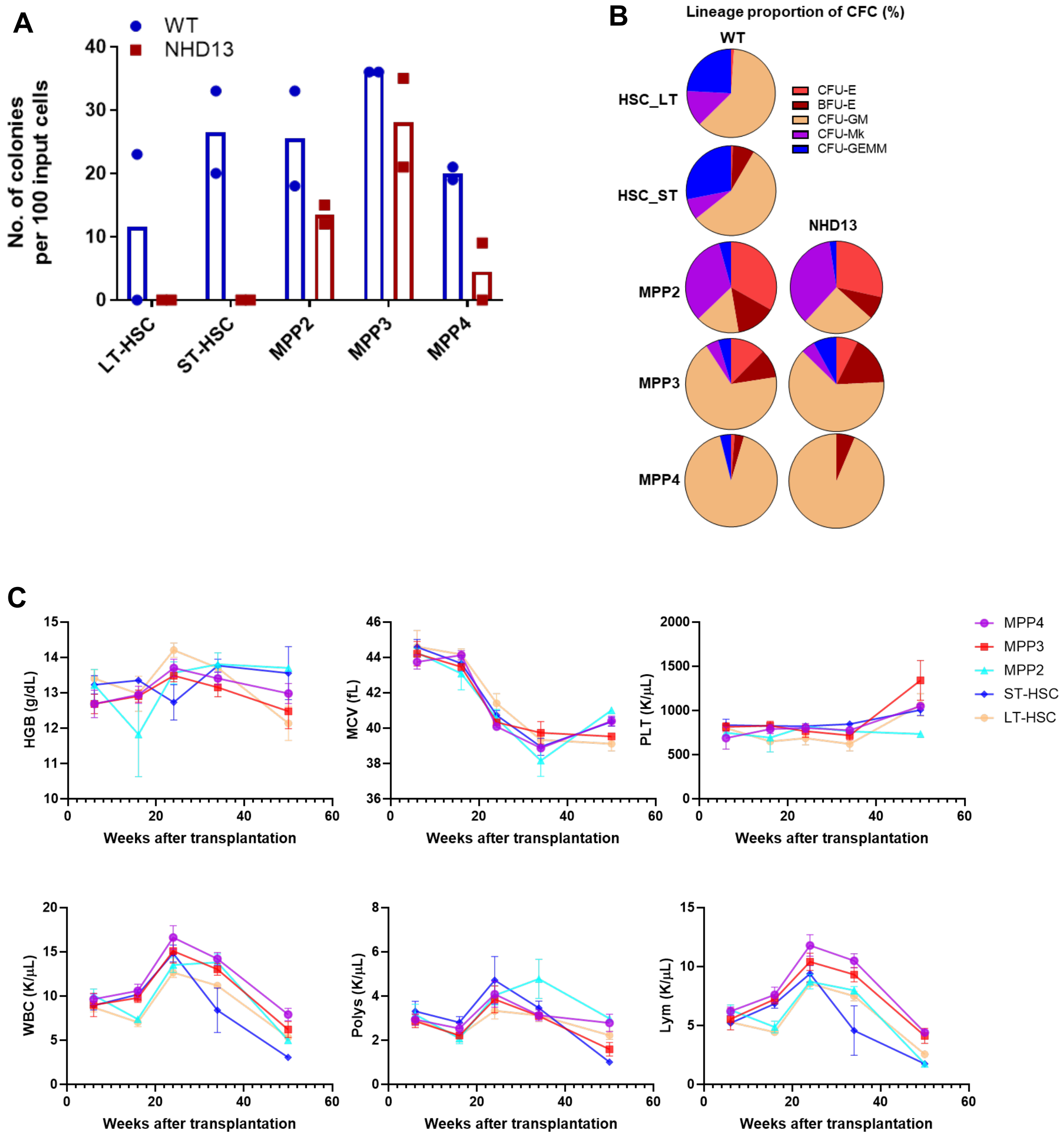

Supplementary figure S2. In vitro colony forming cell (CFC) assay and CBC from recipients of purified NHD13 HSPC. (A) Total number of colonies from each group assessed 10 days after plating. The data from the results of two independent experiments. **(B)** Lineage analysis of colonies from each HSPC population, based on colony morphology. The data from the results of two independent experiments. **(C)** HGB, hemoglobin; MCV, mean corpuscular volume; PLT, platelet; WBC, white blood cell; Polys, neutrophile cells; Lym, lymphoid cells. Recipient mice of each HSPC subset, n=5.

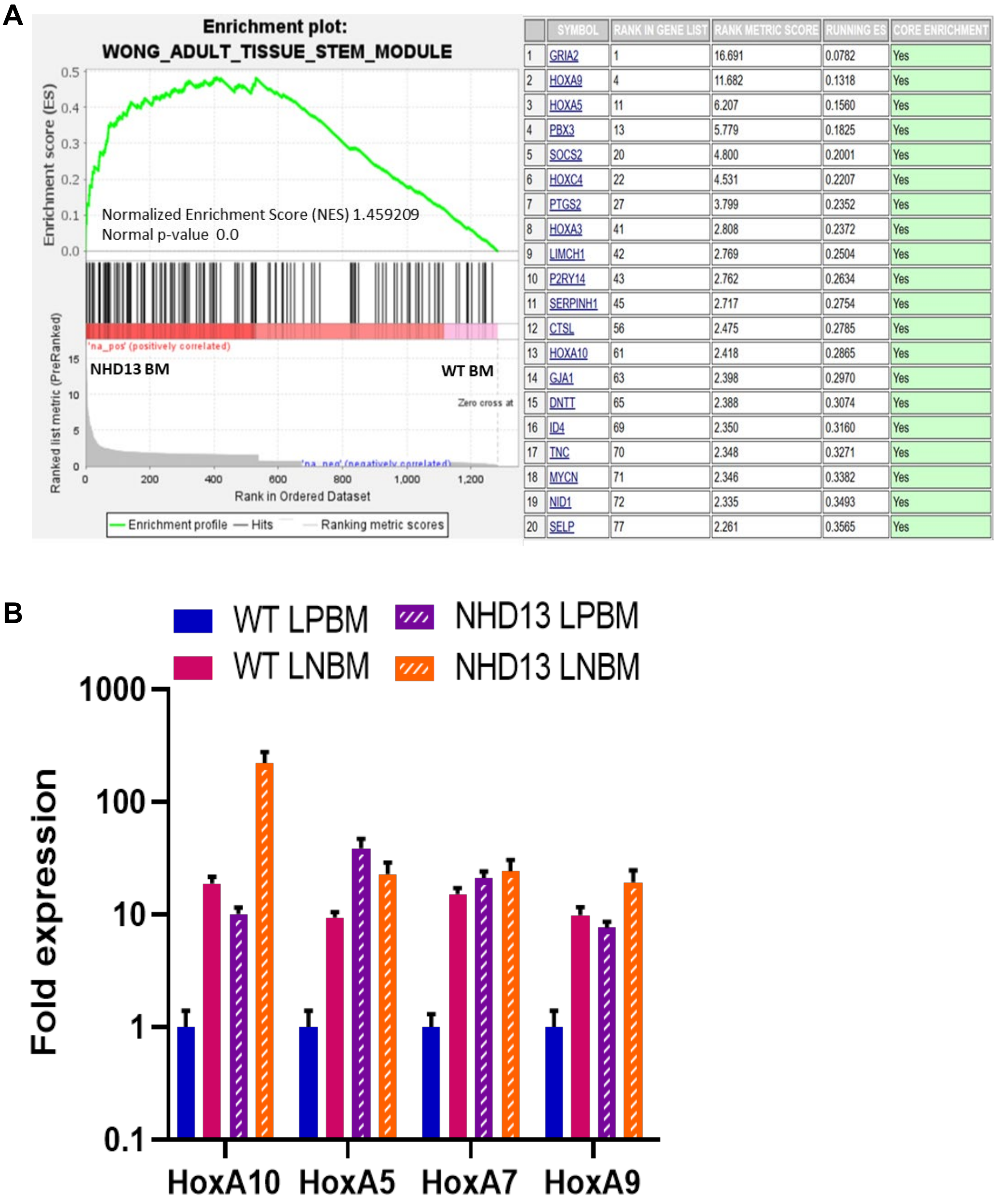

	SYMBOL	RANK IN GENE LIST	RANK METRIC SCORE	RUNNING ES	CORE ENRICHMENT
1	GRIA2	1	16.691	0.0782	Yes
2	HOXA9	4	11.682	0.1318	Yes
3	HOXA5	11	6.207	0.1560	Yes
4	PBX3	13	5.779	0.1825	Yes
5	SOCS2	20	4.800	0.2001	Yes
6	HOXC4	22	4.531	0.2207	Yes
7	PTGS2	27	3.799	0.2352	Yes
8	HOXA3	41	2.808	0.2372	Yes
9	LIMCH1	42	2.769	0.2504	Yes
10	P2RY14	43	2.762	0.2634	Yes
11	SERPINH1	45	2.717	0.2754	Yes
12	CTSL	56	2.475	0.2785	Yes
13	HOXA10	61	2.418	0.2865	Yes
14	GJA1	63	2.398	0.2970	Yes
15	DNTT	65	2.388	0.3074	Yes
16	ID4	69	2.350	0.3160	Yes
17	TNC	70	2.348	0.3271	Yes
18	MYCN	71	2.346	0.3382	Yes
19	NID1	72	2.335	0.3493	Yes
20	SELP	77	2.261	0.3565	Yes

Supplementary figure S3. Gene expression analysis of NHD13 mice vs WT. (A) Gene Set Enrichment Assay with microarray DATA of NHD13 BM vs WT. **(B)** Expression analysis of genes related with HSC self-renewal using RQ-PCR with fractionated BM in terms of lineage positive (LP) antigens or negative (LN) from each genotype of mice. Sample size for WT n=3, and for NHD13 n=3.

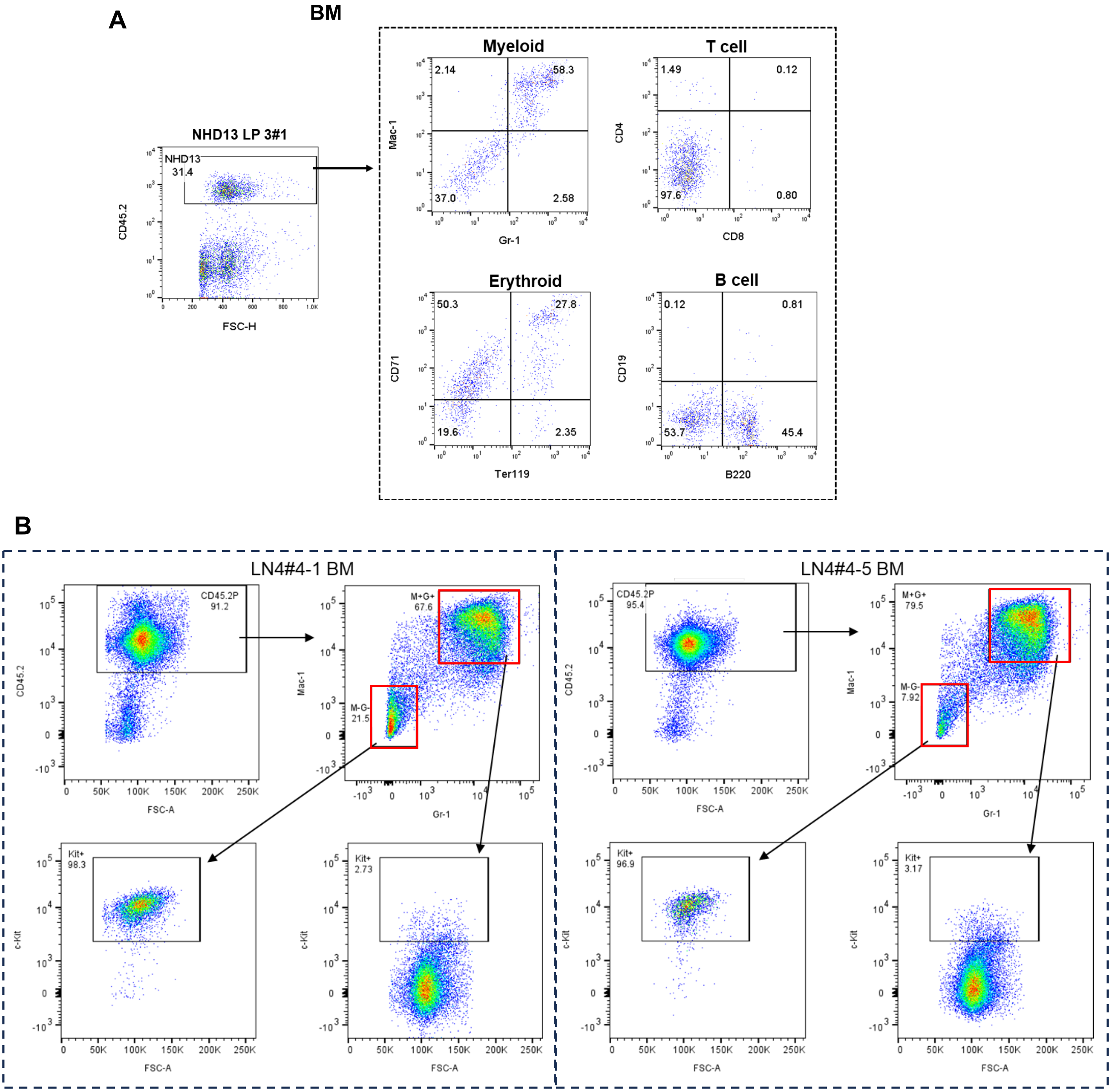

Supplementary figure S4. Flow cytometry profiles demonstrate multi-lineage potential of NHD13 LP BM cells and AML transformation. (A) Representative flow cytometry profiles demonstrate engraftment of Mac1^{+}Gr1^{+} (myeloid), CD71^{+}Ter119^{+} (erythroid), CD4^{+}, CD8^{+} (T-lymphoid), CD19^{+}B220^{+} (B-lymphoid) cells at 59 weeks post HSCT. **(B)** M+G+ indicates double positive of Mac1 and Gr1. M-G-, double negative of Mac1 and Gr1 antigens. Red line boxes indicate sorted population for the WES analysis.

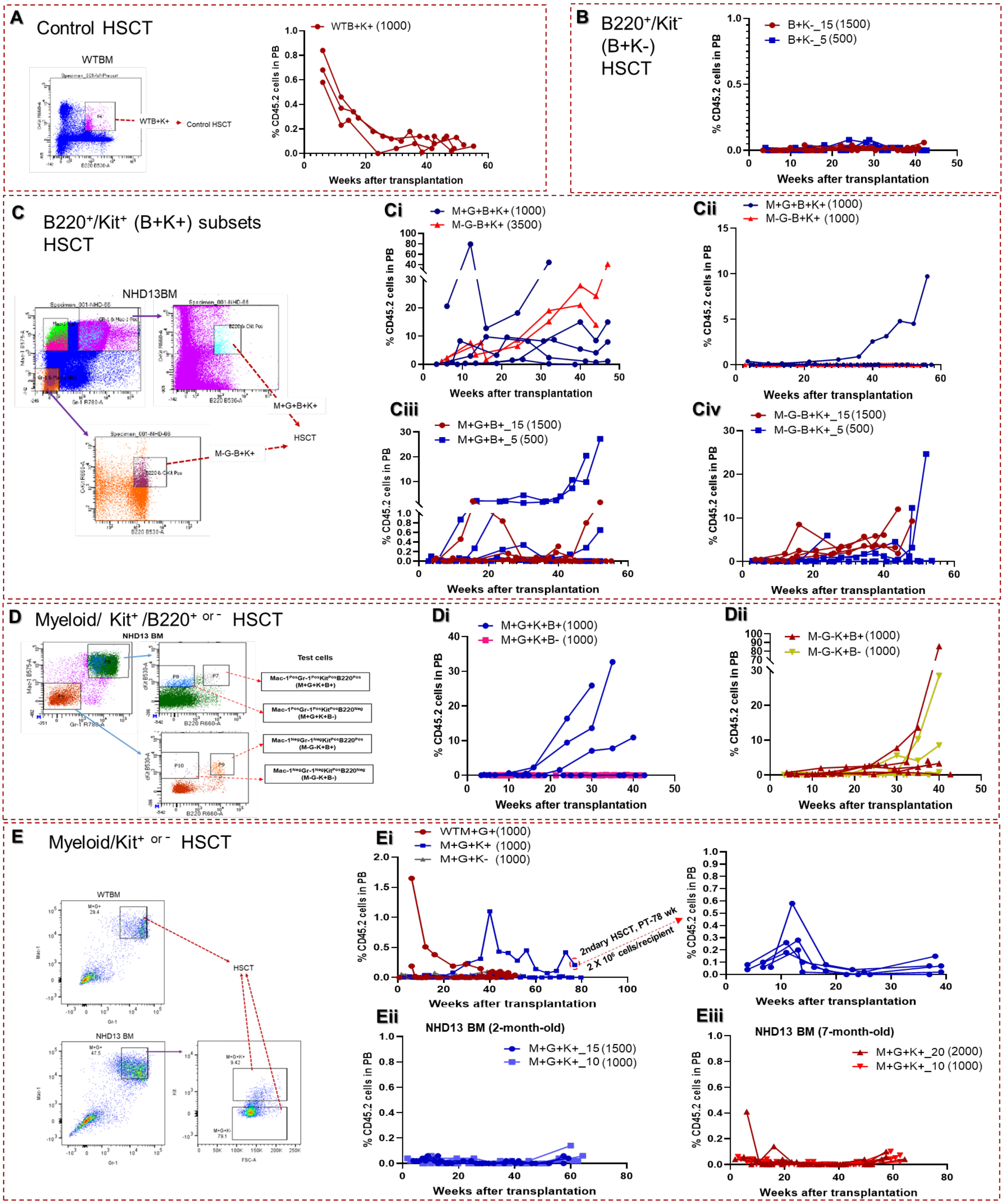

Supplementary figure S5. MDS initiating cell (MIC) may exist in B220 and Kit double positive (B+K+) cells of NHD13 LPBM cells. (A) Control HSCT with WT B+K+ cells. **(B)** NHD13 B220⁺/Kit⁻ cells transplants. **(C)** HSCTs for NHD13 B+K+ cells with or without myeloid markers. Each graph indicates independent experiments. **(D)** HSCT for testing NHD13 Kit⁺/B220⁺ or ⁻ cells with (Di) or without (Dii) myeloid markers. **(E)** Evaluation of NHD13 Kit⁺ or ⁻ cells with myeloid makers. Each roman numberings indicate independent experiments. In figure legends, upper case letters mean antigen positive and lower case, antigen negative. The numbers in the parenthesis next to the legend indicate cell numbers transplanted to given recipients in HSCT assay.

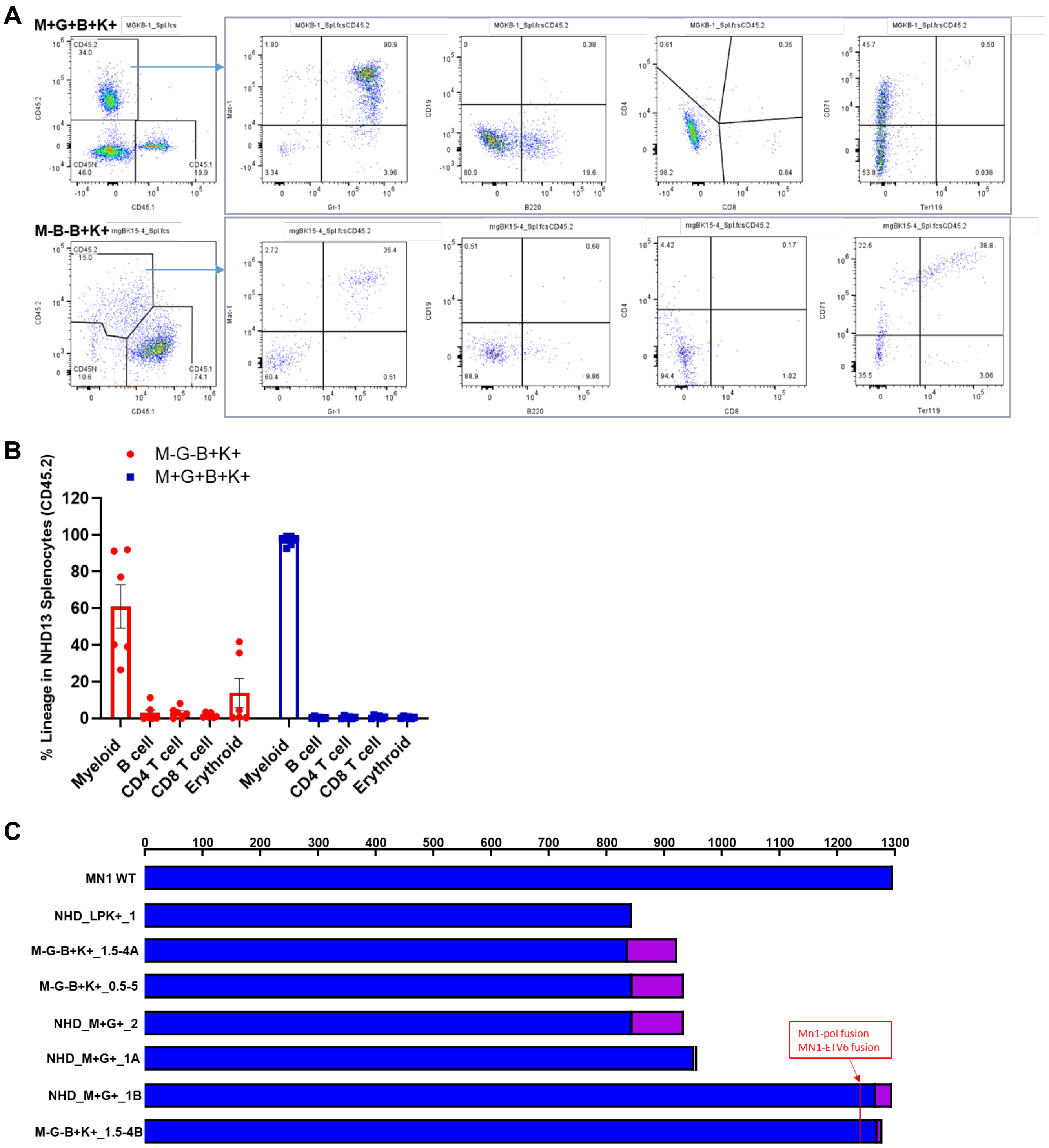

Supplementary figure S6. NHD13 B220 and Kit (B+K+) double positive cells have multilineage potential and frequently acquire Mn1 mutations. (A) Representative flow cytometry profiles demonstrate engraftment of Mac1⁺Gr1⁺ (myeloid), CD71⁺Ter119⁺ (erythroid), CD4⁺, CD8⁺ (T-lymphoid), CD19⁺B220⁺ (B-lymphoid) cells in splenocytes at 30- or 48-weeks post HSCT. **(B)** Summary of differentiated progeny of the NHD13 BK cells in the spleens of recipient mice. Sample size for M-G-B+k+, n=6, and for M+G+B+K+, n=6. Results from 4 independent experiments. **(C)** Mn1 mutant proteins were deduced from the mutation sequence. Amino acid position 1240 of MN1 protein have been reported as a fusion site of Mn1-pol fusion (Cancer Res 2007;67:(11)) and MN1-ETV6 fusion (Leuk Res 2011;35) .

Supplementary Table S1. Meta data of primary donors in this study.

Experiments (HSCT type)	Mouse ID	GT	Age (mo)	HGB (g/dL)	MCV (fL)	PLT (K/uL)	WBC (K/uL)	Polys (K/uL)	Lym (K/uL)	Mono (K/uL)
1st Exp. (LSK)	17	WT	9	na	na	na	na	na	na	na
	18	NHD13	9	na	na	na	na	na	na	na
2nd Exp. (LSK)	67	WT	7	17.0	43.6	1412	10.44	3.08	6.70	0.60
	76	NHD13	6	13.6	51.2	476	2.90	1.45	1.19	0.24
	77	NHD13	6	11.9	60.0	750	2.68	1.17	1.26	0.23
1st Exp. (LSK subset)	543	NHD13	3	13.4	49.5	760	3.80	1.44	2.01	0.34
2nd Exp. (LSK subset)	547	NHD13	4	13.3	48.4	673	3.28	1.73	1.25	0.29
2nd Exp. (Lin^+ or Lin^-)	5969	WT	6	12.8	39.0	1044	6.20	1.54	4.27	0.38
	5970	NHD13	6	11.9	45.8	472	2.82	1.65	0.88	0.25
3rd Exp. (Lin^+ or Lin^-)	7645	WT	3	15.3	40.8	1119	9.34	2.29	6.26	0.62
	7640	NHD13	4	12.5	42.9	595	1.78	0.75	0.85	0.17
4th Exp. (Lin^+ or Lin^-)	7687	WT	5	14.1	41.0	1214	19.2	5.82	11.72	1.31
	7688	NHD13	5	11.6	46.3	549	2.64	1.64	0.87	0.12
5th Exp. (Lin^+ or Lin^-)	7726	WT	5	na	na	na	na	na	na	na
	7735	NHD13	5	11.5	44.6	616	1.96	0.8	1.01	0.13
2nd Exp. (Lin^+ fraction)	5141	NHD13	5	11.9	45.4	647	3.46	1.74	1.38	0.32
3rd Exp. (Lin^+ fraction)	5165	WT	6	12.5	41.2	1635	6.84	0.63	5.74	0.47
	5166	NHD13	6	10.2	48.5	784	3.28	1.60	1.46	0.23
4th Exp. (Lin^+ fraction)	5175	WT	5	13.4	42.0	1068	11.50	1.81	8.97	0.71
	5178	NHD13	5	11.9	45.9	627	2.80	1.29	1.35	0.15
5th Exp. (Lin^+ fraction)	5197	WT	4	14.6	42.0	959	13.68	2.07	10.27	1.03
	5195	NHD13	5	11.9	47.3	616	3.08	1.37	1.43	0.27
6th Exp. (Lin^+ fraction)	5205	NHD13	6	12.6	46.6	606	3.68	1.90	1.47	0.30
7th Exp. (Lin^+ fraction)	5213	NHD13	4	12.3	46.6	598	2.98	1.60	1.17	0.20
8th Exp. (Lin^+ fraction)	5223	NHD13	7	11.3	44.9	515	2.04	1.44	0.35	0.23
	5240	NHD13	2	14.4	42.1	365	4.44	1.11	2.84	0.49
9th Exp. (Lin^+ fraction)	5282	NHD13	6	10.9	48.9	1535	3.40	1.66	1.36	0.37

na means not applicable.

Supplementary Table S2. Incidence of myeloid engraftment (>1%) in recipient mice (Engrafted mice/Transplanted mice).

Experiments	NHD Lin^-	NHD Lin^+	WT Lin^-	WT Lin^+
2nd Exp.	1/3	1/2	4/4	0/4
3rd Exp.	0/3	2/2	1/1	0/3
4th Exp.	2/4	1/5	4/4	0/4
5th Exp.	3/3	1/3	4/4	0/3
Total	6/13 (46%)	5/12 (42%)	13/13 (100%)	0/14 (0%)

Supplementary Table S3. Endpoint data from primary NHD13 recipients

Donor cells of HSCT	Recipient ID	Follow-up (*weeks)	CBC Acquisition (*week)	WBC (K/uL)	ANC (K/uL)	PLT (K/uL)	HGB (g/dL)	MCV (fL)	CD45.2P in BM (%)	Cause of Death	Cell dose per 2° recipient	Diagnosis
Lin+ (LP) BM	LP2#2	32	24	8.34	1.79	609	12.6	45.5	na	Infection (penile wound)	na	MDS
	**LP3#1**	**59**	59	4.99	0.83	1069	13.9	54.6	24.1	**Donor for secondary BMT**	**2.41E+05**	MDS
	**LP3#2**	**59**	59	2.36	0.53	530	13.0	54.7	14.5	**Donor for secondary BMT**	**1.45E+05**	MDS
	**LP4#3**	**47**	46	6.36	2.26	650	13.3	51.8	33.6	**Donor for secondary BMT**	**3.36E+05**	MDS
	LP5#1	78	78	12.26	4.15	811	11.5	48.6	na	Found Dead	na	MDS
Lin^- (LN) BM	LN2#2	58	53	4.98	1.36	1096	11.5	44.9	50	Euthanasia at the endpoint	na	MDS
	**LN4#1**	**58**	58	13.98	6.36	721	12.8	46.5	44.7	**Donor for secondary BMT**	**4.47E+05**	MDS
	**LN4#4**	**58**	58	6.82	1.95	186	10.3	47.6	51.8	**Donor for secondary BMT**	**5.18E+05**	MDS
	LN5#1	59	59	1.78	1.05	259	11.1	53.6	58.9	Euthanasia at the endpoint	na	MDS
	**LN5#2**	**41**	40	6.04	3.07	445	11.0	51.4	43.9	**Donor for secondary BMT**	**4.39E+05**	MDS
	LN5#3	54	54	11.16	1.72	414	8.7	69.2	na	Found Dead	na	MDS

*Weeks post HSCT; na, not applicable; Bold letters mean data of primary recipients used as secondary donors.

Supplementary Table S4. Endpoint data for secondary recipients.

Recipient ID	Follow-up (*weeks)	CBC Acquisition (*week)	WBC (K/uL)	Polys (K/uL)	PLT (K/uL)	HGB (g/dL)	MCV (fL)	Blast count (% BMC)	BM Engraftment (% CD45.2)	PB Engraftment (% CD45.2)	Cause of Death	Comments
LP3#1-**1**	24	24	2.08	0.64	95	1.7	65.1	NA	NA	0.00	Moribund	NHD13 cells engraftment not detected; Blood collection near portal vein.
LP3#1-**2**	31	24	9.26	1.94	654	13.5	46.9	NA	NA	0.14	Unknown	
LP3#1-**3**	34	24	4.52	0.83	609	11.5	47.5	NA	NA	0.60	Unknown	
LP3#2-**2**	59	59	1.94	1.11	360	9.7	53.1	18.1	4.15	0.43	MDS	
LP3#2-**3**	50	50	29.92	10.12	298	8.2	59.6	NA	91.5	79.79	AML	
LP3#2-**4**	59	59	12.58	4.46	474	8.3	56.2	20.7	71.6	82.01	AML	
LP3#2-**5**	43	43	2.6	0.68	630	9.7	54.0	14.5	75.5	54.59	MDS	
LP4#3-**1**	53	53	10.56	3.73	371	7.7	69.3	42.8	94.3	55.35	AML	
LP4#3-**2**	70	70	9.00	5.92	770	12.2	45.1	11.5	54.7	20.16	MDS	
LP4#3-**3**	70	70	3.72	1.66	642	11.9	48.7	25.6	70.1	17.91	AML	
LP4#3-**4**	39	39	57.48	15.52	61	3.2	92.6	NA	NA	90.11	AML	
LN4#1-**1**	53	53	17.96	7.65	212	8.7	56.6	31.3	94.5	77.57	AML	
LN4#1-**2**	53	53	4.06	0.67	381	9.4	53.8	37.9	69.1	42.14	AML	
LN4#1-**3**	44	44	14.12	3.03	98	3.9	82.0	68.5	63.3	52.28	AML	
LN4#1-**4**	6	6	6.98	2.67	504	13.2	47.5	NA	NA	NA	Iatrogenic	Experimental procedure error
LN4#1-**5**	53	53	5.78	2.54	249	4.9	55.8	32.9	70.3	49.61	AML	
LN4#4-**1**	30	30	28.9	3.22	613	7.8	66.8	52.5	80.9	84.20	AML	
LN4#4-**2**	24	24	26.04	5.53	292	10.8	47.1	20.0	88.7	52.17	AML	
LN4#4-**3**	24	24	18.98	2.92	469	9.2	61.1	28.8	84.6	27.11	AML	
LN4#4-**4**	24	24	20.38	5.00	317	9.6	60.1	20.1	88.4	41.24	AML	
LN4#4-**5**	30	30	28.8	6.86	169	9.8	63.3	38.7	93.1	69.81	AML	
LN5#2-**1**	48	48	1.66	1.12	420	11.4	52.5	11.6	60.5	45.59	MDS	
LN5#2-**2**	48	48	3.04	1.39	611	14.7	45.2	12.6	NA	NA	Euthanized	NHD13 cells engraftment not detected at the time of euthasia
LN5#2-**3**	48	45	5.2	2.18	662	10.4	44.2	NA	NA	NA	Unknown	NHD13 cells engraftment not detected at post-transplanation 45 week in PB
LN5#2-**4**	38	38	3.9	2.1	154	3.0	48.2	36.9	23.0	0.4	AML	

*Weeks post HSCT

LP, lineage positive cells from primary donor; LN, lineage negative cells from primary donor

Supplementary Table S5. Acquired mutations in recipient mice transplanted with Lin^{-} (LN) or Lin^{+} (LP) cells from NHD13 BM.

ID	Experiment	Donor	Recipient Type	CD45.2 Purity (%)	Dagnosis	Gene	Nucleotide	Amino Acid	VAF
LN4#4	4th	NHD13 (7688) Lineage Negative BM cells	Primary	51.8	MDS	Nfic	1176_1181 dupGGCCAT	A393_I394 insMA	0.158
						Tlr5	124G>A	G42S	0.118
						Spag8	1028delG	R343fs	0.176
						Ptprv	2744T>C	V915A	0.571
						Stxbp6	527G>A	R176H	0.357
						Slc17a4	532G>T	G178C	0.364
						Dpp9	37G>A	A13T	0.250
						Vwa2	112G>A	A38T	0.750
						Fam110a	461delG	R154fs	0.400
						Abca14	1810G>C	N604H	0.412
LN4#4-1_MG	4th	LN4#4 recipient WBM cells	Secondary	98.4 (sorted BM, Mac1^{+} Gr-1^{+} Kit^{-})	AML	Ptpn11	205G>A	E69K	0.375
						Kras	34G>A	G12S	0.148
						Nfic	1176_1181 dupGGCCAT	A393_I394 insMA	0.429
						Tlr5	124G>A	G42S	0.667
						Tigit	395T>A	V132E	0.667
						Spag8	1028delG	R343fs	0.409
						Pnpla8	860G>A	R287H	0.310
LN4#4-1_Kit	4th	LN4#4 recipient WBM cells	Secondary	99.8 (sorted BM, Mac1^{-} Gr1^{-} Kit^{+})	AML	Ptpn11	205G>A	E69K	0.214
						Kras	34G>A	G12S	0.250
						Nfic	1176_1181 dupGGCCAT	A393_I394 insMA	0.533
						Tlr5	124G>A	G42S	0.448
						Tigit	395T>A	V132E	0.625
						Spag8	1028delG	R343fs	0.464
						Pnpla8	860G>A	R287H	0.256
						Lhb	401A>C	H134P	0.304
						Fank1	737C>T	T246M	0.273
LN4#4-2	4th	LN4#4 recipient WBM cells	Secondary	89.9	AML	Kit	2472T>G	N824K	0.913
						Nfic	1176_1181 dupGGCCAT	A393_I394 insMA	0.111
						Tlr5	124G>A	G42S	0.500
						Tigit	395T>A	V132E	0.462
						Spag8	1028delG	R343fs	0.448
LN4#4-3	4th	LN4#4 recipient WBM cells	Secondary	86.4	AML	Nfkb2	1880A>G	E627G	0.389
						Nfic	1176_1181 dupGGCCAT	A393_I394 insMA	0.346
						Tlr5	124G>A	G42S	0.371
						Tigit	395T>A	V132E	0.400
						Spag8	1028delG	R343fs	0.361
						Sft2d2	252G>A	M84I	0.235
LN4#4-4	4th	LN4#4 recipient WBM cells	Secondary	89.8	AML	Cbl	1090-2A>T		0.500
						Nfic	1176_1181 dupGGCCAT	A393_I394 insMA	0.643
						Tlr5	124G>A	G42S	0.656
						Tigit	395T>A	V132E	0.444
						Spag8	1028delG	R343fs	0.609
LN4#4-5_MG	4th	LN4#4 recipient WBM cells	Secondary	98.9 (sorted BM, Mac1^{+} Gr-1^{+} Kit^{-})	AML	Nras	34G>T	G12C	0.500
						Nfic	1176_1181 dupGGCCAT	A393_I394insMA	0.364
						Tlr5	124G>A	G42S	0.313
						Tigit	395T>A	V132E	0.667
						Spag8	1028delG	R343fs	0.556
						Kif21a	2555G>A	R852Q	0.300
						Tgm6	1432G>A	V478M	0.500
						Slco1a4	862G>A	E288K	0.485
						Tex264	461C>A	A154D	0.667
LN4#4-5_Kit	4th	LN4#4 recipient WBM cells	Secondary	99.8 (sorted BM, Mac1^{-} Gr1^{-} Kit^{+})	AML	Nras	34G>T	G12C	0.506
						Nfic	1176_1181 dupGGCCAT	A393_I394 insMA	0.462
						Tlr5	124G>A	G42S	0.648
						Tigit	395T>A	V132E	0.500
						Spag8	1028delG	R343fs	0.420
						Kif21a	2555G>A	R852Q	0.692
						Tgm6	1432G>A	V478M	0.429
						Slco1a4	862G>A	E288K	0.377
						Actb	850A>G	K284E	0.367
						Foxp1	1315A>C	T439P	0.225
LP3#2-3	3rd	LP3#2 recipient WBM cells (LP3#2 is a primary recipient transplanted with Lineage Positive BM cells of NHD13 (7640) mouse)	Secondary	93.2	AML	Etv6	592delG	E198fs	0.684
						Trp53	808C>T	R270C	0.389
						Kras	38G>A	G13D	0.276
						Mina	652G>A	G218S	0.389
						Mob3a	332C>T	T111M	0.655
						Ldah	562C>T	R188*	0.472
						Prl5a1	284T>C	V95A	0.455
						Thoc3	374G>A	R125H	0.286
						Ptprg	2914A>G	N972D	0.615
						Fam110a	34G>C	A12P	0.667
						Taf13	238C>T	R80*	0.421
						Hyal5	1288T>C	S430P	0.435
LP3#2-5	3rd	LP3#2 recipient WBM cells (LP3#2 is a primary recipient transplanted with Lineage Positive BM cells of NHD13 (7640) mouse)	Secondary	72.5	MDS	Jarid2	1160_1161 insGG	K388fs	0.455
						Mina	652G>A	G218S	0.500
						Ndrg2	718dupC	R240fs	0.435
						Pyroxd2	564G>T	Q188H	0.375
						Rbm20	2554G>A	G852R	0.300
						Hyal5	1288T>C	S430P	0.348
						Col5a3	2066C>T	P689L	0.257
						Sidt2	1429A>T	I477F	0.231

Red letters indicate mutations associated with hematologic malignancy. Blue letters highlight mutations present in donor BM.

Supplementary Table S6. Total time from primary transplant to AML in secondary recipients.

Primary recipients	Weeks (Post-BMT)	Secondary recipients	Weeks (Post-BMT)	Total Weeks	Total Months
LP3#2	59	LP3#2-2	59	118	29.5
		LP3#2-3	50	109	27.3
		LP3#2-4	59	118	29.5
		LP3#2-5	43	102	25.5
LP4#3	46	LP4#3-1	53	99	24.8
		LP4#3-2	70	116	29.0
		LP4#3-3	70	116	29.0
		LP4#3-4	39	85	21.3
Mean					**27.0**
SD					**3.0**

Supplementary Table S7. Engraftment of lineage positive NHD13 sub-populations.

	B220+ Kit+ cells		Kit+ B220- cells		Kit- B220- cells	
	Mice tested	Mice engrafted	Mice tested	Mice engrafted	Mice tested	Mice engrafted
	55	28	29	1	15	0
Ratio		0.51		0.03		0.00

	M+G+B+K+ cells	
	Mice tested	Mice engrafted
	34	13
Ratio		0.38

	M-G-B+K+ cells	
	Mice tested	Mice engrafted
	21	14
Ratio		0.67

M+, Mac1 positive; G+, Gr1 positive; M-, Mac1 negative; G-, Gr1 negative; B+, B220 positive; K+, Kit positive. B220+Kit+ cells were further separated using Mac1 and Gr1 antibodies into M+G+B+K+ and M-G-B+K+ populations.

Supplementary Table S8. Endpoint data for recipients of isolated NHD13 sub-populations.

Exp.	Recipient ID	Follow-up (*weeks)	WBC (K/uL)	Poly (K/uL)	PLT (K/uL)	HGB (g/dL)	MCV (fL)	Blast count (%BMC)	BM Engraftment (%CD45.2)	Cause of Death
2nd	NHD LPK+_1	48	1.80	0.57	413	11.2	49.0	18.7	75.0	MDS
	NHD LPK+_2	48	2.44	0.87	662	11.7	48.6	13.0	76.7	MDS
	NHD LPK+_3	48	4.22	0.75	509	6.9	56.9	36.1	38.5	AML
	NHD LPK+_4	40	4.96	1.49	457	9.8	46.7	33.5	89.3	AML
	NHD M+G+_1	48	7.32	3.63	1019	12.3	43.9	17.4	48.1	MDS
	NHD M+G+_2	48	2.54	0.73	813	8.2	49.7	15.3	53.1	MDS
3rd	M+G+B+K+_1	47	8.5	5.26	1072	10.1	45.8	18.8	79.6	MDS
	M+G+B+K+_2	47	10.16	4.11	1043	11.1	40.7	10.6	10.9	MDS
	M+G+B+K+_3	32	42.22	22.67	298	11.8	52.8	28.6	54.2	T ALL (recipient)
	M+G+B+K+_5	47	15.58	9.67	1162	10.3	37.2	16.8	9.03	MDS
	M-G-B+K+_1	46	5.5	2.47	573	13	47.6	11.4	88.8	MDS
	M-G-B+K+_2	46	na	na	na	na	na	na	na	MDS
4th	M+G+B+K+_4	56	4.78	2.74	531	12.1	42.6	na	na	MDS
5th	M+G+K+_3	78	9.42	4.03	796	12.2	41.2	8.9	1.23	MDS
6th	M+G+B+_5-5	74	9.06	3.58	972	9.1	48.5	29.9	74.6	MDS
	M+G+B+_5-3	48	9.54	3.91	1212	12.2	41.2	7.2	76.6	MDS
	M+G+B+_5-4	74	28.24	14.94	311	7.7	56.6	31.7	89.8	AML
	M+G+B+_5-5	52	17.02	10.17	1347	10.6	47.6	10.1	86.1	MDS
7th	M-G-B+K+_1.5-1	41	4.24	0.96	929	10.4	41.4	8.6	20.6	MDS
	M-G-B+K+_1.5-2	40	4.96	1.22	1363	7.3	43.8	32.3	68.9	AML
	M-G-B+K+_1.5-3	44	3.72	0.79	953	9.7	45.2	10.4	69.0	MDS
	M-G-B+K+_1.5-4	48	6.00	1.78	1949	12.8	47.6	11.5	52.9	MDS
	M-G-B+K+_0.5-2	48	3.94	2.03	1218	11.6	43.9	7.1	36.4	MDS
	M-G-B+K+_0.5-5	52	6.46	2.45	1216	10.5	44.2	13.1	36.3	MDS
9th	M+G+B+K+_1	30	12.24	3.4	945	8.5	41.2	8.3	67.3	MDS
	M+G+B+K+_2	52	2.68	2.06	904	8.0	43.7	16.6	50.5	MDS
	M+G+B+K+_4	35	5.64	1.18	304	7.3	50.4	26.4	59.8	AML
	M-G-B+K+_1	52	3.22	1.15	646	7.1	44.1	31.4	42.6	T ALL (immature)
	M-G-B+K+_2	40	258.2	55.33	818	9.4	61.1	89.4	82.8	T ALL (immature)
	M-G-B+K+_3	56	3.84	1.62	726	7.4	49.7	19.7	49.4	AML
	M-G-B+K+_4	60	6.38	2.84	168	4.5	52.3	34.0	54.5	AML
	M-G-B-K+_1	56	55.9	33.93	389	8.1	48.6	59.0	77.4	T ALL (immature)
	M-G-B-K+_2	72	10.16	3.84	1923	10.3	45.3	12.4	31.3	MDS
	M-G-B-K+_4	72	4.74	2.85	501	8.7	45.1	17.9	36.0	MDS
	M-G-B-K+_5	48	7.8	3.36	727	8.3	47.9	34.6	70.4	AML

*Weeks post HSCT. M+, Mac1 positive; G+, Gr1 positive; M-, Mac1 negative; G-, Gr1 negative; B+, B220 positive; K+, Kit positive.

Supplementary Table S9. Acquired mutations in recipient mice transplanted with purified NHD13 BM sub-populations.

ID	Experiment	Donor	Recipient Type	CD45.2 Purity (%)	Diagnosis	Gene	Nucleotide	Amino Acid	VAF
LPK+_1	2nd	5141 LPK	Primary	75	MDS	Mn1	2536G>T	E846*	0.222
						Prrt3	1328A>C	N443T	0.326
						Trbd1 -> (Trbj1-1, 2, 3, 4, 5), (Trbj2-1, 3, 4, 5)[a]			≥0.2
LPK+_2	2nd	5141 LPK	Primary	76.7	MDS	Mn1	3021_3022insAGGG	S1008Efs*12	0.148
LPK+_3	2nd	5141 LPK	Primary	99.0	AML	Kprp	806G>A	R269H	0.217
						Rars2	1268C>T	T423M	0.205
						Dync1li2	1004C>T	A335V	0.211
M+G+_1	2nd	5141 MG	Primary	99.4	MDS	Mn1	2851_2858dup	P954Gfs*6	0.127
						Mn1	3802_3803dup	D1268Efs*30	0.126
						Otulin	717_718del	R239Sfs*7	0.411
						Trbd1 -> Trbj1-4 ; Trbd1 -> Trbj2-5			≥0.2
M+G+_2	2nd	5141 MG	Primary	99.4	MDS	Hoxb2	266dup	A90Sfs*75	0.500
						Mn1	2540_2541dup	D848Pfs*89	0.422
						Pgls	582_583insCCCTC	A195Pfs*20	0.416
M+G+B+K+_1	3rd	5166 MGBK	Primary	79.6	MDS	Cyp4b1	734G>A	R245H	0.321
M+G+B+K+_5	3rd	5166 MGBK	Primary	71.9	MDS	Setd1a	3850_3851 insTC	D1284Vfs*30	0.232
M-G-B+K+_1	3rd	5166 mgBK	Primary	88.8	MDS	Etv6	771_772 ins AGATT	R258*	0.375
						Vmn2r6	1676A>C	H559P	0.376
						Ankzf1	2012T>G	F671C	0.212
						Arap3	4571A>C	Q1524P	0.204
M+G+B+_15-5	6th	5205 MGB	Primary	99.5	MDS	Irf2bp2	1458_1459dup	L487Pfs*77	0.500
						Btbd18	1674G>C	E558D	0.487
						Rapgef1	2215G>C	D739H	0.479
						Kcnf1	1098C>A	F366L	0.291
M+G+B+_5-3	6th	5205 MGB	Primary	99.6	MDS	Btbd18	1674G>C	E558D	0.448
						Rapgef1	2215G>C	D739H	0.469
						Cst13	135G>T	Q45H	0.518
M+G+B+_5-4	6th	5205 MGB	Primary	89.8	AML	Tnfrsf22	88_90dup	L30dup	0.417
						Btbd18	1674G>C	E558D	0.400
						Rapgef1	2215G>C	D739H	0.400
						Kcnk10	1123C>T	R375W	0.517
						Zfp618	1817C>T	S606L	0.491
						C1galt1	329C>T	T110M	0.466
						Sec14l3	953G>A	G318E	0.378
						Fam124a	529C>T	R177C	0.358
						Nol11	2099G>A	R700Q	0.343
M+G+B+_5-5	6th	5205 MGB	Primary	86.1	MDS	Pik3c2a	2441dup	S815Ifs*25	0.313
						Rapgef1	2215G>C	D739H	0.468
						Btbd18	1674G>C	E558D	0.415
						Serpinb5	527C>T	P176L	0.272
M-G-B+K+_1.5-1	7th	5213 mgBK	Primary	98.8	MDS	Tmem266	871C>T	R291C	0.412
						Ptk7	89C>T	P30L	0.375
M-G-B+K+_1.5-2	7th	5213 mgBK	Primary	99.8	AML	Nras	34G>A	G12S	0.436
						Nr0b2	392dup	S131Rfs*33	0.405
						Cfap157	358_359insT	A120Vfs*12	0.419
						Cmtm7	443C>A	A148E	0.322
M-G-B+K+_1.5-3	7th	5213 mgBK	Primary	98.7	MDS	Trp53	716G>A	C239Y	0.250
						Ptk7	89C>T	P30L	0.357
						Pde8b	1625G>A	S542N	0.295
M-G-B+K+_1.5-4	7th	5213 mgBK	Primary	99.7	MDS	Mn1	3812_3819del	A1271Vfs*11	0.247
						Mn1	2514_2542del	K839Qfs*7	0.123
						Tmem266	871C>T	R291C	0.417
						Ptk7	89C>T	P30L	0.533
						Thbs2	1384C>T	R462C	0.272
						Mrpl24	386A>C	K129T	0.210
						Mup10	10A>C	M4L	0.258
M-G-B+K+_0.5-2	7th	5213 mgBK	Primary	99.0	MDS	Ston1	1432C>G	L478V	0.229
						Spty2d1	541G>A	E181K	0.209
						Stac	127C>T	R43W	0.216
M-G-B+K+_0.5-5	7th	5213 mgBK	Primary	98.6	MDS	Mn1	2542_2543insTGGG	D848Gfs*9	0.426
						Tmem266	871C>T	R291C	0.291
						Ptk7	89C>T	P30L	0.344
						Zbtb24	484C>T	R162W	0.377
						Tiam2	2880C>G	N960K	0.330
						Zfp462	4507G>A	V1503I	0.380

Red letters indicate mutations associated with hematologic malignancy. Blue letters highlight mutations present in donor BM. M+, Mac1 positive; G+, Gr1 positive; M-, Mac1 negative; G-, Gr1 negative; B+, B220 positive; K+, Kit positive.
