# Supplementary material for: Disruption of normal stem cell function and transmission of myelodysplastic syndrome by self-renewal of committed myeloid lineage cells
Source: Stem Cell Reports. 2025 Jul 3;20(8):102571. doi: 10.1016/j.stemcr.2025.102571 (PMC12365822; doi:10.1016/j.stemcr.2025.102571)
Supplement: Document S2. Article plus supplemental information [file mmc2.pdf]

Stem Cell Reports
Article
OPEN ACCESS

Disruption of normal stem cell function and transmission of myelodysplastic syndrome by self-renewal of committed myeloid lineage cells

Yang Jo Chung,[1] Ryan Bertoli,[1] Dengchao Cao,[1] Robert L. Walker,[1] Yuelin Jack Zhu,[1] Paul Meltzer,[1] and Peter D. Aplan[1,2,3,*]
[1]Genetics Branch, Center for Cancer Research, National Cancer Institute, National Institutes of Health, Bethesda, MD, USA
[2]Myeloid Malignancies Program, National Institutes of Health, Bethesda, MD, USA
[3]Lead contact
*Correspondence: aplanp@mail.nih.gov
https://doi.org/10.1016/j.stemcr.2025.102571

SUMMARY

The ineffective hematopoiesis of myelodysplastic syndrome (MDS) suggests that hematopoietic stem and progenitor cells (HSPCs) are defective. Here, we demonstrate that *NUP98::HOXD13* (*NHD13*) MDS mice have significantly decreased functional HSPCs. Moreover, in contrast to wild-type (*WT*) bone marrow (BM), lineage-positive (Lin^+) BM cells from *NHD13* mice have self-renewal potential. Specific subsets of *NHD13* Lin^+ cells that express B220 and Kit antigens were able to self-renew and generate MDS in *WT* recipients. Although this unique $B220^+Kit^+$ phenotype could be found in *WT* as well as *NHD13* BM, the population was markedly increased in *NHD13* BM. Further characterization using Mac1 and Gr1 markers revealed that both $Mac1^+Gr1^+B220^+Kit^+$ and $Mac1^-Gr1^-$ $B220^+Kit^+$ populations showed self-renewal and led to an MDS phenotype in *WT* recipients. Taken together, these findings demonstrate that as normal hematopoiesis derived from typical HSPCs decreases in *NHD13* mice, committed hematopoietic progenitor cells proliferate, self-renew, and initiate MDS.

INTRODUCTION

The myelodysplastic syndromes (MDSs) represent a heterogeneous group of clonal hematopoietic stem cell (HSC) disorders and are a significant cause of morbidity and mortality. In general, the incidence of MDS increases with age, and the median age at diagnosis is 70 years old (Maynadie et al., 1996). Although some forms of MDS can be considered a pre-leukemic condition, MDS represents a disease entity that is distinct from acute myeloid leukemia (AML) (Nimer, 2008). Using xenograft mouse models, AML-initiating cells have been thoroughly characterized (Bonnet and Dick, 1997; Hope et al., 2004; Lapidot et al., 1994; Shlush et al., 2014). However, isolation and characterization of MDS-initiating or stem cells using xenotransplantation of human cells from patients with MDS has been challenging due to relatively low-level engraftment of MDS cells, even in severely immunodeficient mice. Moreover, the engrafted cells do not consistently generate the characteristic features of MDS in mice (Benito et al., 2003; Kerbauy et al., 2004; Nilsson et al., 2002; Thanopoulou et al., 2004), possibly because the murine hematopoietic system does not produce an environment (including cytokines, chemokines, and cell-cell interaction) that will support the entire spectrum of human hematopoietic differentiation. Therefore, to avoid issues caused by immunodeficiency and cross-species barriers, we used murine MDS cells within the context of a murine host to identify MDS stem cells.

We employed a mouse model of MDS based on the expression of a *NUP98::HOXD13* (*NHD13*) fusion gene, which was initially cloned from a pediatric patient with MDS (Raza-Egilmez et al., 1998; Slape et al., 2008). This model faithfully recapitulates all of the key features of MDS, including peripheral blood (PB) cytopenias, maturation arrest, bone marrow (BM) dysplasia, and transformation to acute leukemia in approximately 60% of mice (Lin et al., 2005). Using this model, prior studies have demonstrated that MDS can be transferred to healthy recipients via HSC transplantation (HSCT), suggesting the existence of an MDS stem cell (Chung et al., 2008).

In this study, we demonstrate that normal hematopoiesis rapidly diminishes as *NHD13* mice age and can be replaced by hematopoiesis derived by extended self-renewal of committed, lineage-positive (Lin^+) cells. We further demonstrate that hematopoiesis derived from these Lin^+ cells can be transplanted to wild-type (WT) recipients, leading to MDS and extensive self-renewal of these committed cells, culminating in AML transformation after an extensive incubation period (up to 27 months) and acquisition of mutations in collaborating genes *in vivo*.

RESULTS

NHD13 mice show progressive pancytopenia and loss of hematopoietic stem and progenitor cells with age

We and others have previously shown that *NHD13* mice invariably develop MDS, with most mice showing anemia and leukopenia by 5–6 months of age (Balderman et al.,

Published by Elsevier Inc. on behalf of International Society for Stem Cell Research.
This is an open access article under the CC BY license (http://creativecommons.org/licenses/by/4.0/).

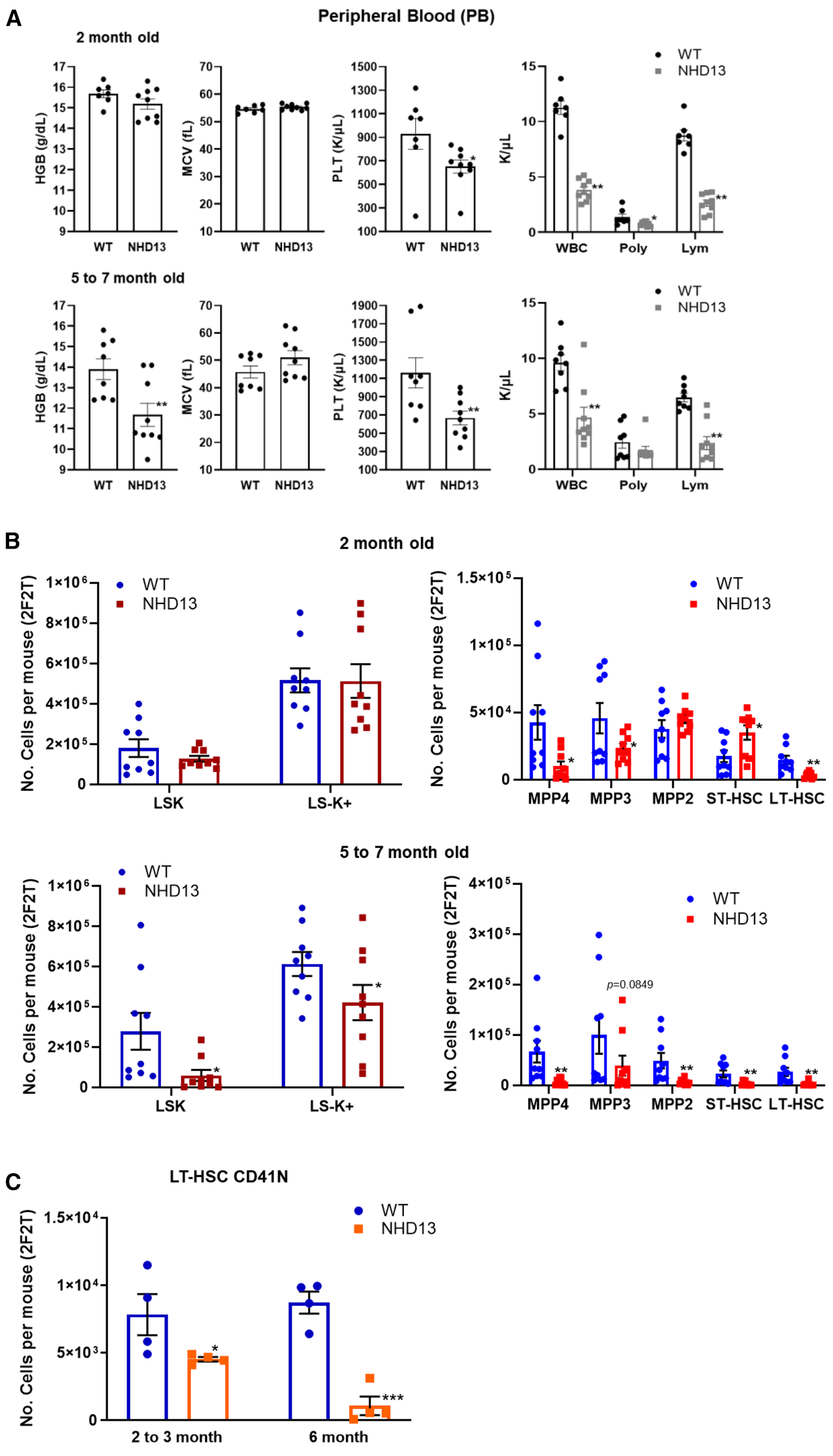

Figure 1. Hematopoietic abnormalities in *NHD13* mice progress with age

(A) Complete blood count (CBC) from age-matched *WT* ($n = 9$) and *NHD13* ($n = 9$) mice: *< $p = 0.05$; **< $p = 0.01$.

(B) HSPC analysis from age-matched *WT* ($n = 9$) and *NHD13* ($n = 9$) mice: *< $p = 0.05$; **< $p = 0.01$.

(C) Numbers of CD41 N (negative) LT-HSCs at 2 and 6 months of age: *WT* $n = 4$, *NHD13* $n = 4$; LN, lineage negative; MPP, multi-potential progenitor; 2F2T, two femora and two tibiae per mouse; *< $p = 0.05$; ***< $p = 0.001$.

2016; Gough et al., 2012; Lin et al., 2005; Nimer, 2008). However, young (2 months old) *NHD13* mice show minimal PB abnormalities, principally, lymphopenia and mild thrombocytopenia and neutropenia (Figure 1A). Anemia (Hgb 11.6 ± 0.5 vs. 13.9 ± 0.5) is the earliest sign of MDS in this model and typically becomes evident in adult (>5 months) *NHD13* mice (Figure 1A). To determine if these age-dependent changes in PB (Gough et al., 2012) indices

reflected progressive changes in the hematopoietic stem and progenitor cell (HSPC) compartment with age, we assessed HSPC number in young and adult *NHD13* mice. Two-month-old *NHD13* mice have normal numbers of lineage-negative (Lin$^-$), Sca1$^+$, Kit$^+$ (LSK) and LS$^-$K$^+$ cells, but further fractionation shows a 4.7-fold decrease in long-term (LT) HSCs and a compensatory increase in short-term (ST) HSCs in the *NHD13* BM. However, consistent with anemia and thrombocytopenia, older (5–7 months) *NHD13* mice show a >30% decrease in LS$^-$K$^+$ cells, a 4-fold decrease in LSK cells, and a marked decrease in LT- and ST-HSCs compared to age-matched *WT* mice (Figures 1B and S1A). The decreased number of LT-HSC in 2-month-old *NHD13* mice indicates that the defect in early hematopoietic differentiation precedes the most marked PB abnormalities. To assess whether increased proliferation of LSK subsets could compensate for decreased numbers of LSK subsets, we injected mice with bromodeoxyuridine (BrdU) 48 h prior to harvesting BM. There was no consistent difference in the percent of cycling LSK subsets (as determined by BrdU incorporation), except a modest increase in *NHD13* ST-HSCs (Figures S1B and S1C).

CD41 expression on the surface of adult LT-HSCs is dependent on the age and functional potential of HSPCs. CD41$^-$ LT-HSCs are thought to reside at the apex of the hematopoietic hierarchy and to be more quiescent and less frequently dividing than CD41$^+$ LT-HSCs (Bernitz et al., 2016; Gekas and Graf, 2013; Yamamoto et al., 2013). Young *NHD13* mice showed a modest decrease in the absolute number of quiescent CD41$^-$ LT-HSCs, and this reduction became more severe in adult *NHD13* mice (Figures 1C and S1D). In contrast, *WT* mice maintained similar numbers of CD41$^-$ LT-HSCs in young and adult mice (Figure 1C). Taken together, these results suggest that the MDS phenotype in *NHD13* mice is associated with loss of the most primitive self-renewing HSPCs in the BM.

HSPCs from *NHD13* mice show decreased self-renewal *in vitro* and *in vivo*

We used functional *in vitro* and *in vivo* assays to identify a potential MDS stem cell within the LSK population. An *in vitro* colony-forming cell (CFC) assay (Ito et al., 2003) was used to assess colony formation of flow-sorted LSK subsets from *NHD13* mice. MPP2 and MPP3 cells produced a decreased number of colonies compared to *WT*, while MPP4 cells, ST-HSCs, and LT-HSCs from *NHD13* mice produced no colonies (Figure S2A). The proportions of burst forming unit erythroid (BFU-E), colony forming unit erythroid (CFU-E), colony forming unit granulocyte macrophage (CFU-GM), colony forming unit megakaryocyte (CFU-Mk), and colony forming unit granulocyte erythroid macrophage megakaryocyte (CFU-GEMM) generated by *NHD13* MPP2 and MPP3 were similar to those of *WT* mice (Figure S2B).

We previously reported that MDS was transplantable as a disease entity to healthy recipient mice via HSCT (Chung et al., 2008). Additional experiments by us and others (Balderman et al., 2016; Cheng et al., 2017) have shown that mice transplanted with non-fractionated *NHD13* BM will invariably engraft and that *NHD13* cells will eventually outcompete the *WT* cells (Balderman et al., 2016; Cheng et al., 2017; Chung et al., 2008). In an effort to identify an MDS-initiating cell (MIC), five HSPC subpopulations (MPP2/3/4, ST-HSCs, and LT-HSCs) from young *NHD13* BM (3–4 months old) were purified using flow cytometry and transplanted into lethally irradiated (900 cGy) mice (3–5 recipients each subpopulation) along with 5×10^5 competitor cells (Figure 2A). The *NHD13* cells expressed the CD45.2 isoform, while the competitor cells expressed CD45.1. Serial PB engraftment assays were followed for 1 year (Figures 2B and S2C). Given that one of the hallmarks of MDS is ineffective hematopoiesis, we also searched for *NHD13* cells in the BM of recipient mice at 17 weeks after transplant. With one exception (a faint band in MPP#2), there was no evidence of engraftment of any HSPC subpopulation in the recipients (Figure 2C). A limitation of this experiment is the low number (14–143 cells) of cells transplanted per mouse. However, the results obtained by transplantation of *NHD13* LSK subsets were consistent with those obtained by transplantation of unfractionated *WT* and *NHD13* LSK cells. All (4/4) mice transplanted with 200 *WT* LSK cells engrafted, whereas 0/5 mice transplanted with 200 *NHD13* LSK cells engrafted (Figure 2D). Taken together, these results indicate that *NHD13* HSPC subsets lack effective self-renewal potential and do not successfully engraft recipient mice.

Committed progenitor cells of *NHD13* MDS mice have self-renewing potential

Although it has been well documented that murine WT committed, Lin$^+$ BM cells will not self-renew (Spangrude et al., 1988) and that HSPCs lie within the LSK compartment, the lack of engraftment by *NHD13* LSK subpopulations suggested the intriguing possibility that the population responsible for MDS transmission may not lie within the LSK population. Our initial experiments assessed whether the cell type responsible for engraftment and self-renewal might be found in a "committed" Lin$^+$ cell population. To limit cross-contamination of cell types, we employed widely separated flow cytometry sorting gates for Lin$^+$ and Lin$^-$ cells from 3- to 6-month-old *WT* or 4- to 6-month-old *NHD13* mice (Figure 3A; Table S1). As in prior experiments, the *NHD13* cells expressed CD45.2, while the *WT* recipient and competitor cells expressed CD45.1. As expected, *WT* Lin$^-$ cells engrafted all recipient

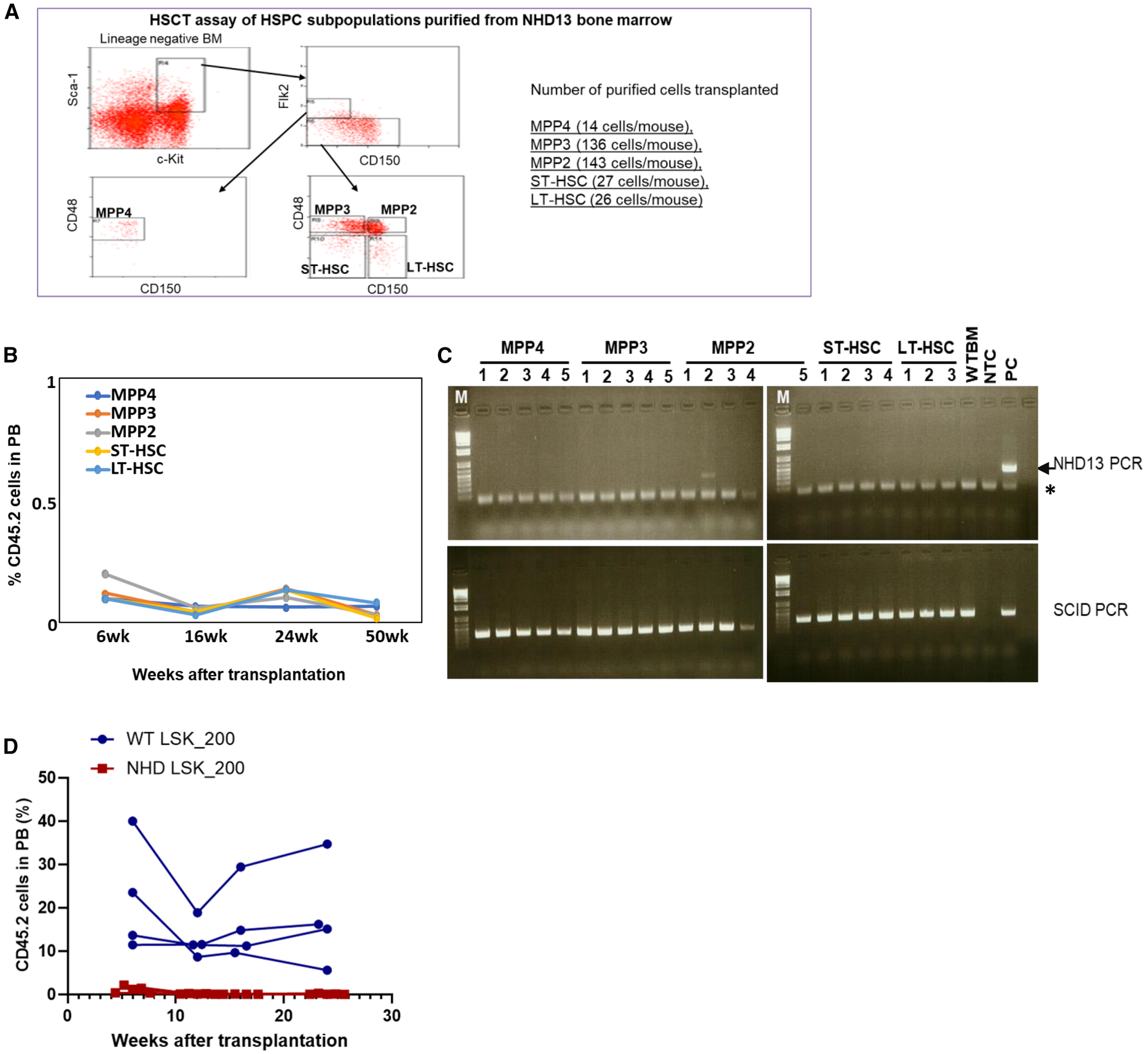

Figure 2. Engraftment of purified HSPC subpopulations from *NHD13* bone marrow

(A) Diagram of the experiment and cell sorting.

(B) Engraftment of CD45.2$^+$ *NHD13* cells at indicated time post transplant: MPP4 $n = 5$, MPP3 $n = 5$, MPP2 $n = 5$, ST-HSCs $n = 4$, LT-HSCs $n = 3$.

(C) Donor cell engraftment assessed by PCR. Genomic DNA prepared from BM cell aspirated from femur of recipient mice at post-transplantation week 17; PC, positive control for *NHD13* transgene; NTC, no template control; * indicates non-specific band, likely primer dimers. The results shown represent one of two independent experiments.

(D) Two independent experiments, HSCT of 200 LSK cells from either *WT* or *NHD13* BM: WT LSK_200 $n = 4$, NHD LSK_200 $n = 5$.

mice in all experiments (Figure 3B), while only one mouse engrafted any *WT* Lin$^+$ cells (Figure 3C, mouse 4#3). Further investigation demonstrated that this mouse had engrafted only mature CD19$^+$B220$^+$ B cells, which gradually diminished in number.

6 of 13 mice transplanted with Lin$^-$ *NHD13* cells showed evidence of engraftment, typically beginning >20 weeks post transplant (Figure 3D). However, in contrast to the lack of engraftment of *WT* Lin$^+$ cells, 5 of 12 mice transplanted with *NHD13* Lin$^+$ cells showed LT engraftment (Figure 3E; Table S2), at 20–60 weeks of age, indicating that a self-renewing, Lin$^+$ cell had been transplanted. In several cases (3#1, 3#2, 5#1; Figure 3E), there is only low-level engraftment (<1.0%) until 40 weeks

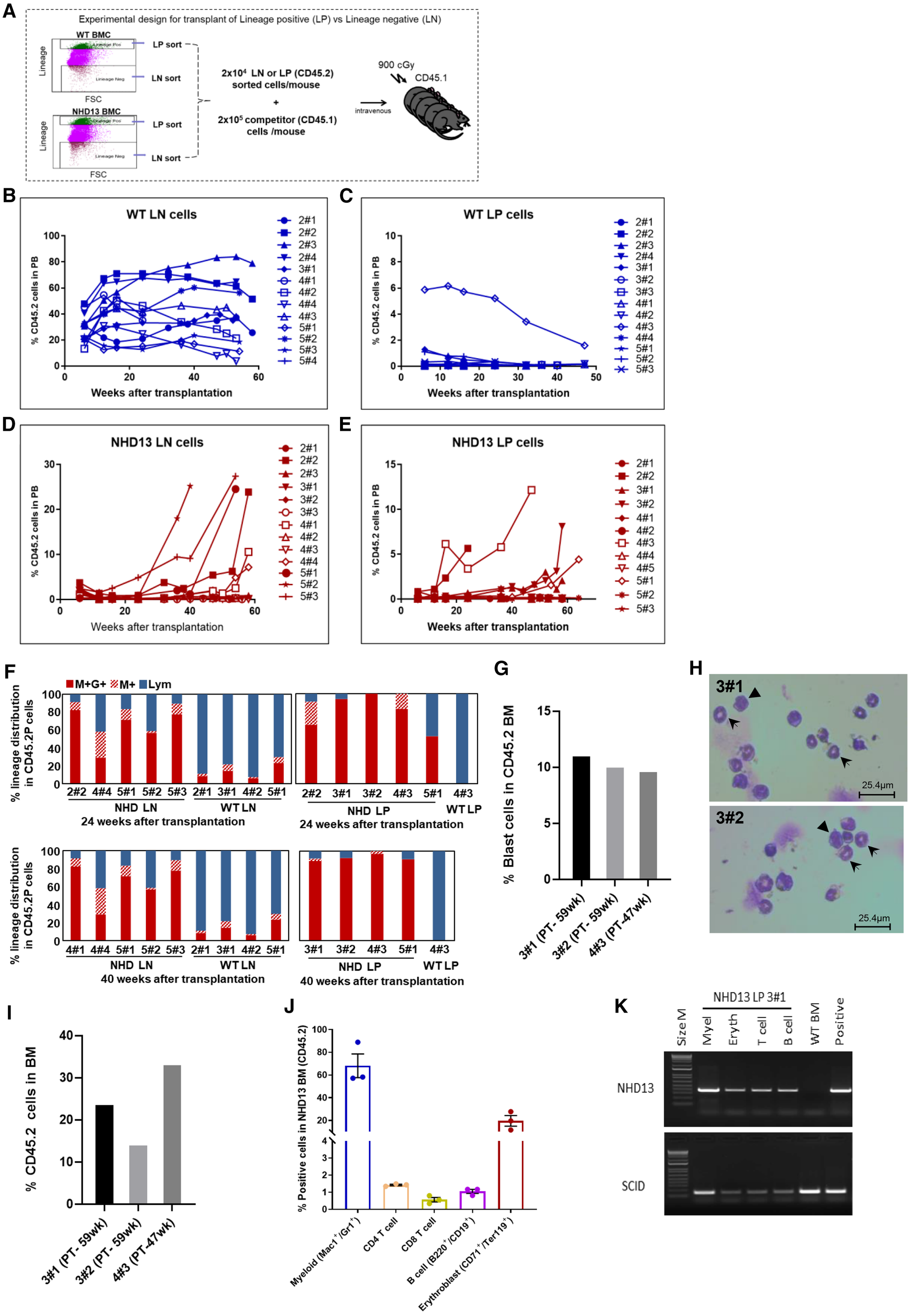

(legend on next page)

post transplant. Transplant of either Lin⁻ or Lin⁺ *NHD13* cells showed a marked skewing toward myeloid cells, which progressed with age (Figure 3F). Comparison of a publicly available gene expression profile of *NHD13* BM and *WT* BM (Novak et al., 2012) using gene set enrichment analysis (GSEA) revealed enrichment of stem cell module gene sets in *NHD13* BM. The GSEA leading-edge analysis showed enhanced expression of *Hoxa5*, *7*, *9*, and *10*; this was confirmed by quantitative reverse-transcription PCR (RT-qPCR) comparison of RNA isolated from *NHD13* Lin⁺ BM cells to RNA isolated from *WT* Lin⁺ BM cells (Figure S3).

To rule out the possibility that the engraftment of *NHD13* Lin⁺ cells represented engraftment of fully transformed AML cells, we euthanized three engrafted mice from this experiment and assessed the percentage of CD45.2 engraftment, and blast counts. Mice euthanized 47–59 weeks post transplant had 24.1% ± 9.6% CD45.2 cells in the BM, less than half of which displayed blast morphology (Figures 3G–3I). The CD45.2 cells were underrepresented in the PB (7.4% ± 5.1%) compared to the BM (24.1% ± 9.6%), consistent with the ineffective hematopoiesis previously reported in the *NHD13* MDS model (Chung et al., 2008). Flow cytometry using lineage commitment markers revealed multilineage hematopoiesis, characterized by the presence of myeloid, erythroid, and lymphoid cells derived from the *NHD13* Lin⁺ cells in the BM (Figures 3J and S4). Cells of *NHD13* origin were identified initially by CD45.2 staining (Figures 3J and S4) and confirmed by PCR amplification of the *NHD13* transgene (Figure 3K).

In addition, despite a median follow-up of 58 weeks (range 24–78) post transplant, none of the recipients showed evidence of transformation to frank AML (Table S3). These results suggest that the impaired engraftment potential of the *NHD13* Lin⁻ cells is compensated by acquisition of engraftment and self-renewal by *NHD13* Lin⁺ cells, supporting the hypothesis that hematopoiesis in older *NHD13* mice is at least partially derived from "committed" Lin⁺ cells.

Unique immunophenotypic and functional characteristics of *NHD13* LP cells

We used both immunophenotypic and functional assays to characterize the Lin⁻ and Lin⁺ cells that had demonstrated LT engraftment of *WT* recipients (Figure 4A). We first determined the fraction of Lin⁻ and Lin⁺ cells in recipients that had been transplanted with *NHD13* Lin⁻ (CD45.2⁺) and *WT* BM competitor cells (CD45.1⁺). As shown in Figure 4B, these mice were chimeric, with approximately 50% CD45.2⁺ (*NHD13* derived) and 50% CD45.2⁻ (*WT* derived). Both the CD45.2⁺ (*NHD13*) and CD45.2⁻ (*WT*) cells contained similar percentages of Lin⁻ and Lin⁺ cells. However, although BM from *NHD13* Lin⁺ recipients also showed similar percentages of CD45.2⁺ and CD45.2⁻ cells (Figure 4C), BM derived from *NHD13* Lin⁺ cells (CD45.2⁺) showed a marked decrease in Lin⁻ cells compared to those derived from *WT* BM competitor cells (CD45.2⁻) (Figure 4C, right panel). As shown in Figure 4D, recipients of Lin⁻ *NHD13* cells had 2.98% ± 1.1% Lin⁻ cells, whereas recipients of LP *NHD13* cells had only 0.43% ± 0.3% Lin⁻ cells, a 6.5-fold difference ($p = 0.00981$). These results indicate that the extent of Lin⁻ cell repopulation in *NHD13* BM cells is dependent on the donor cell type, either Lin⁻ or Lin⁺ BM cells.

BM repopulated from *NHD13* Lin⁺ donors showed a markedly diminished ability to generate LSK cells compared with *WT* BM donors (Figure 4E, left panel). In contrast, BM repopulated by *NHD13* Lin⁻ BM showed similar numbers of LSK cells compared to *WT* BM (Figure 4E, right panel). The proportions of *WT* LSK in the recipients of *NHD13* Lin⁻ or Lin⁺ BM were similar, suggesting that there is not a major influence of the *NHD13* cells (Lin⁻ or Lin⁺) on the engraftment and expansion of *WT* BM (Figure 4E). Taken together, these results suggest that *NHD13* Lin⁺ BM has the ability to self-renew and produce

Figure 3. Engraftment and multi-lineage potential of *NHD13* Lin⁺ BM cells
(A) Schematic outline of the experiment. Four- to 6-month-old *NHD13* or *WT* mice served as donors.
(B–E) Peripheral blood engraftment of Lin⁻ (LN) or Lin⁺ (LP) cells from *WT* or *NHD13* donors. Results represent individual mice from four independent experiments (designated by experiment number and mouse number); blue lines indicate engraftment from *WT* LN or LP donor cells. Dark red lines indicate engraftment from *NHD13* LN or LP donor cells.
(F) Percentage of myeloid and lymphoid cells at 24 and 40 weeks post transplant (PT): M+, Mac1 single positive; M+ G+, Mac1 and Gr1 double-positive; Lym, lymphoid.
(G) Percent blast cells in *NHD13* Lin⁺ BM cells at time of euthanasia.
(H) Morphology of CD45.2-purified BM cells from *NHD13* LP BM recipients. BM cells were purified using magnetic cell sorting (MACS) with a CD45.2 antibody and stained with May-Giemsa (MG) stain (400X); arrowhead, blast cell; arrow, ring neutrophil.
(I) Percent chimerism of *NHD13* cells in *NHD13* LP BM recipients.
(J) Summary of differentiated progeny of the *NHD13* Lin⁺ BMCs in recipient mice ($n = 3$).
(K) Detection of the *NHD13* transgene in flow-sorted populations. Amplification of the *Scid* locus is used as a DNA quality control; decreased intensity of the Scid PCR product is expected for erythroid, T, and B cells as fewer cells were recovered after sorting.

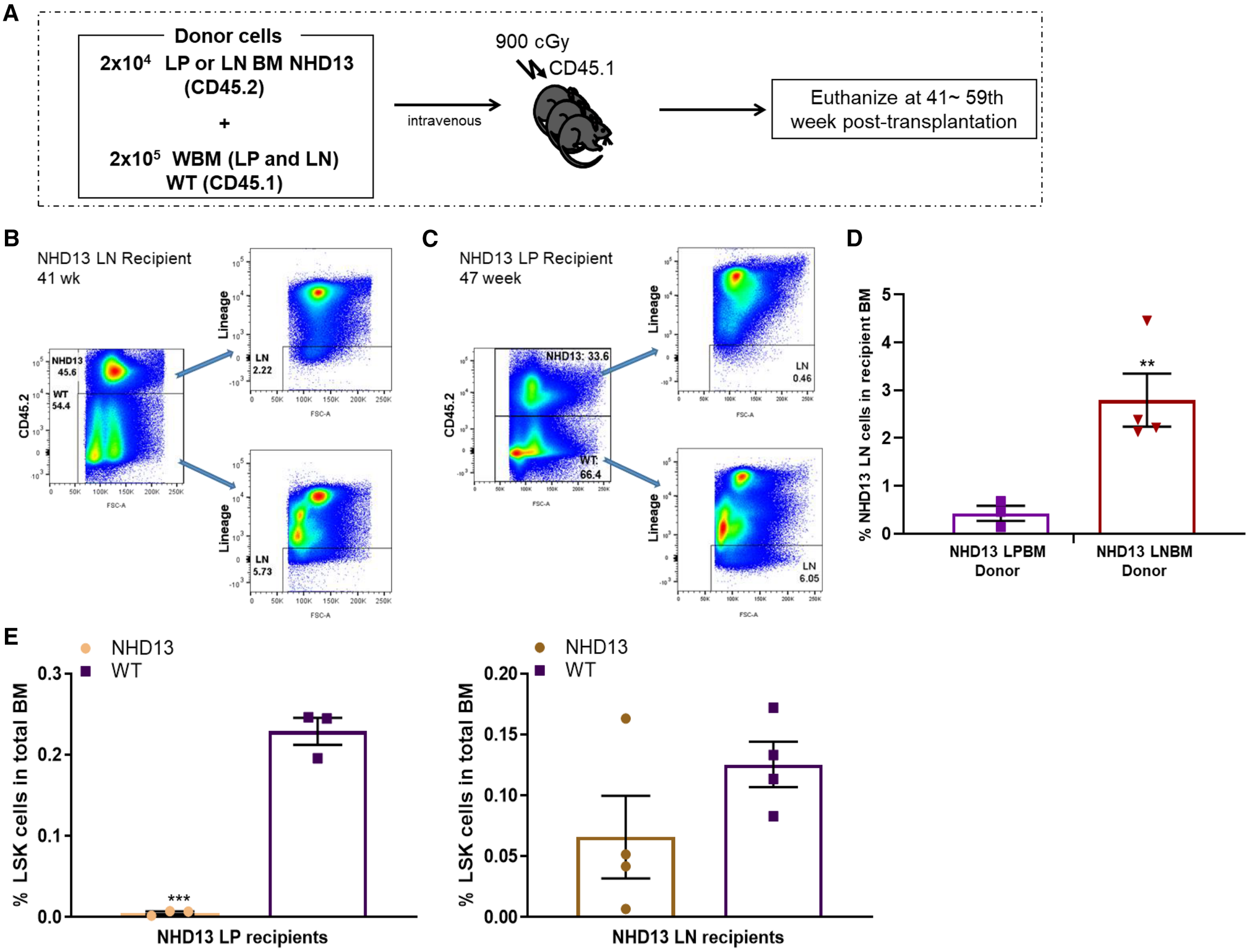

Figure 4. Distinct repopulation patterns from *NHD13* Lin$^+$ or Lin$^-$ BM cells

(A) Schematic of the experiment.

(B) Representative flow cytometry profiles from *NHD13* LN recipient.

(C) Representative flow cytometry profiles from *NHD13* LP recipient.

(D) Percent LN BM cells in LN ($n = 4$) vs. LP ($n = 3$) recipients: **< $p = 0.01$.

(E) HSPC repopulation in each transplant group: NHD13 $n = 3$, WT $n = 3$ in *NHD13* LP recipients; NHD13 $n = 4$, WT $n = 4$ in *NHD13* LN recipients; ***< $p = 0.001$.

Lin$^+$ cells directly, as opposed to de-differentiating to *NHD13* Lin$^-$ cells, followed by forward differentiation to Lin$^+$ cells.

To determine the *in vivo* functionality of cells derived from *NHD13* Lin$^-$ or Lin$^+$ cells, we assessed the ability of these cells to engraft recipient mice in a secondary transplant assay. Secondary HSCT recipients received 1×10^6 unfractionated BM cells from primary recipients (Figures 3D and 3E). Since the primary recipients were chimeric, the total number of *NHD13* cells transplanted varied between 1.4 and 5.2×10^5 cells (Table S3). The secondary HSCT demonstrated that the *NHD13* Lin$^-$ BM cells successfully engrafted most recipients, with the majority of engrafted recipients eventually transforming to AML (Figure 5A; Table S4). Similar results were seen with the secondary HSCT from the *NHD13* Lin$^+$ donors (Figure 5B; Table S4). Of note, secondary recipients of *NHD13* Lin$^-$ BM showed an engraftment pattern that was dependent on the primary donor. For instance, recipients of 4#4 BM (red squares) showed a high engraftment at 6 weeks post transplant (43.1% ± 3.1%), followed by a nadir of 9.66% ± 1.8% engraftment at 12 weeks, culminating with a terminal increase to 32.5%–77.0% at 24–30 weeks post transplant (Figure 5A). Recipients of 4#1 and 5#2 BM (blue circles, yellow diamonds) showed modest initial engraftment (from week 6–30), with a terminal increase in weeks 40–52. In contrast, secondary recipients of *NHD13* Lin$^+$ BM all showed a pattern that was independent of

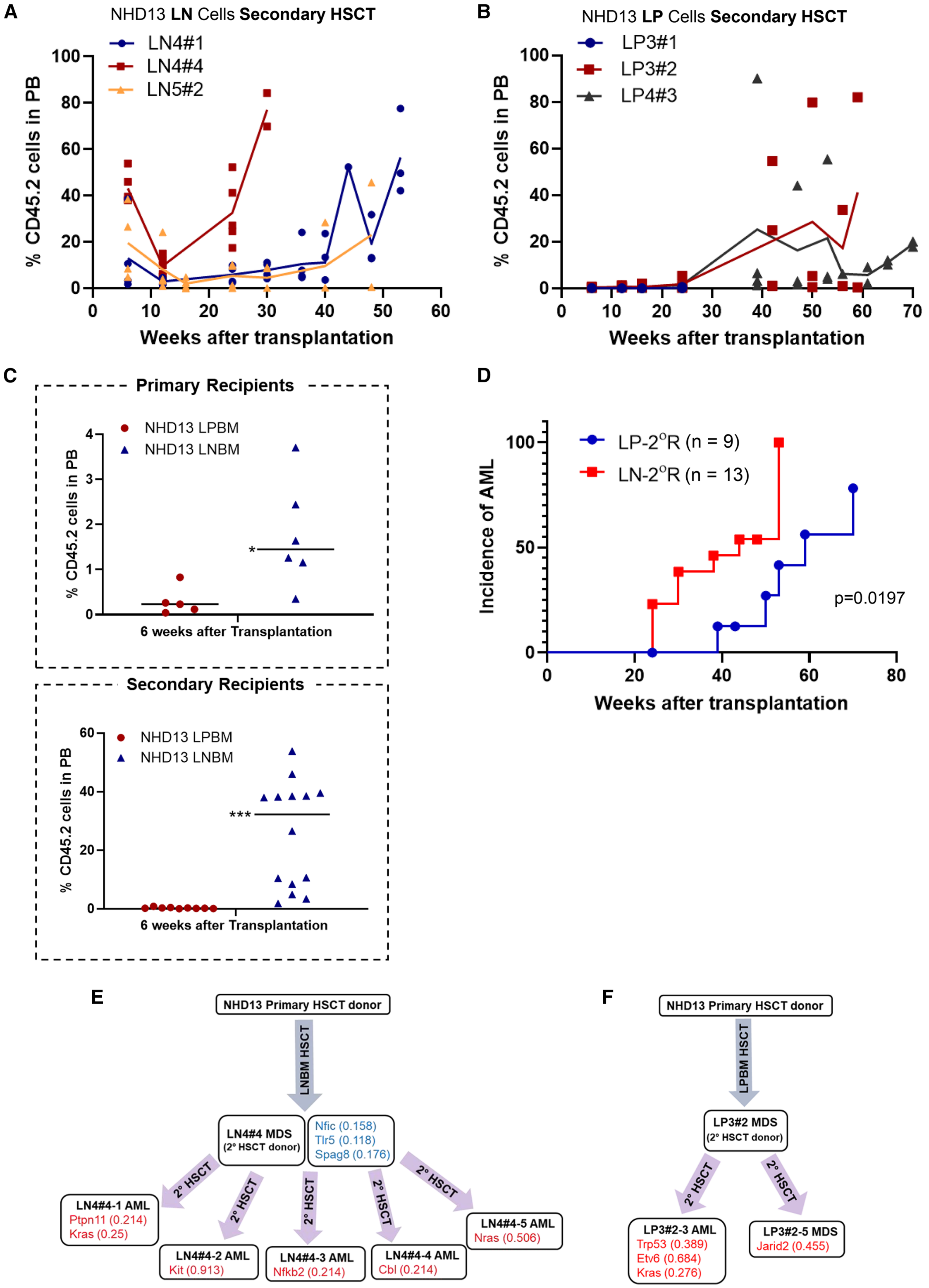

Figure 5. Distinct long-term repopulation features of *NHD13* Lin$^+$ vs. Lin$^-$ BM cells

(A) Secondary recipients of *NHD13* LN BM cells: LN4#1 *n* = 5, LN4#4 *n* = 5, LN5#2 *n* = 4.

(B) Secondary recipients of *NHD13* LP BM cells: LP3#1 *n* = 3, LP3#2 *n* = 5, LP4#3 *n* = 4.

(legend continued on next page)

the primary donor, with minimal initial engraftment (<2%) in all recipients up to 12 weeks, followed by a highly variable terminal engraftment at weeks 30–70 (Figure 5B).

Primary recipients of *NHD13* Lin^- BM showed increased engraftment compared to Lin^+ BM recipients (Figure 5C, upper panel). However, the secondary recipients of Lin^- BM showed a more dramatic difference in the rapidly engrafting population (Figure 5C, lower panel). Although most mice in both the Lin^- and Lin^+ secondary HSCT groups developed AML, the Lin^- recipients developed AML more rapidly (median 44 vs. 59 weeks; Figure 5D; Table S4). Taken together, these secondary transplant results suggest that a pre-leukemic, but not fully transformed, self-renewing population exists within the Lin^+ compartment.

We used whole-exome sequencing (WES) to identify acquired mutations in MDS and AML samples from primary and secondary transplants. Comparison of AML or MDS tissue to normal tissue from the same individual was of limited utility in identifying acquired mutations, since the leukemias were derived from donor mice (NIH C57BL/6 *NHD13* CD45.2 breeding colony) and the normal tissues were from recipient mice (Charles River C57BL/6 CD45.1). Therefore, we compared the AML/MDS samples to pooled tail DNA (n = 3) from the *NHD13* breeding colony, using Mutect or Dragen software and recommended criteria (BaitRegion = TRUE, FILTER = PASS, Impact = MODERATE or HIGH, variant allele frequency [VAF] > 0.2). We eliminated any identical single-nucleotide variants (SNVs) that were present in mice from different experiments (using different donors), as previous studies have shown that these are most likely rare germline SNVs that were present in the breeding colony as opposed to acquired oncogenic SNVs (Goldberg et al., 2017). We next eliminated any SNVs that were also identified in *WT* tissue from recipient mice as these were due to contaminating *WT* tissue in the tumor sample. We were left with 5–12 acquired tier I SNVs per sample (Table S5).

The LN4#4 sample (used as donor for the secondary transplant) had acquired a prominent, but not dominant, clone characterized by tier I variants in *Tlr5*, *Nfic*, and *Spag8*, with a VAF of 0.12–0.18. These variants, as well as a variant in the *Tigit* gene, are present in all seven samples from the recipient mice with a mean VAF of 0.48 ± 0.08, suggesting that this clone had a fitness advantage *in vivo*. All samples had acquired at least one additional mutation in signaling pathway genes that are well known to be involved in AML, including *Ptpn11*, *Kras*, *Nras*, *Kit*, *Cbl*, and *Nfkb2* (Figure 5E). Of note, none of the acquired mutations were the same between different recipients, indicating significant diversity in collaborating events that occurred as the disease evolved from MDS to AML. However, acquired oncogenic mutations in the flow-sorted subsets (Figure S4B) from LN4#4-1 and LN4#4-5 were identical (Table S5), leading us to suspect that the difference in antigen expression (Mac1/Gr1 vs. Kit) in those mice was due to epigenetic, as opposed to genetic, events. In contrast to the findings with LN4#4 recipients, the two recipients of BM from donor LP3#2 had clearly distinct clones, one with oncogenic mutations involving *Trp53*, *Etv6*, and *Kras* and the other with a *Jarid2* frameshift mutation (Table S5). Of note, the recipient with a signaling mutation (*Kras*) evolved to AML, whereas the recipient with an epigenetic mutation (*Jarid2*) died of MDS without transformation to AML (Figure 5F). Taken together, these results demonstrate that *NHD13* LP cells can persist and self-renew for an extended period of time (27 ± 3 months; Table S6) before ultimately transforming to AML, following acquiring mutations in genes well known to be relevant for human MDS/AML (Makishima et al., 2017; Sperling et al., 2017).

MICs are enriched in a $B220^+$/Kit^+ population

Given that the Lin^+ cells utilized in the aforementioned experiments consist of a heterogeneous population of cells, we fractionated Lin^+ cells using cell surface markers that we thought might contain a self-renewing MIC. Because the self-renewing MICs were myeloid biased, we tested $Mac1^+Gr1^+$ cells, and because Kit expression is often associated with stem cell self-renewal (Rojas-Sutterlin et al., 2014), we tested Kit^+ cells. Flow-sorted $Mac1^+Gr1^+$ and Lin^+Kit^+ cells isolated from *NHD13* BM were transplanted along with *WT* competitor cells, as outlined in Figure 6A (Table S1). Both the $Mac1^+Gr1^+$ and Lin^+Kit^+ populations were able to self-renew and engraft; however, all (4/4) mice transplanted with Lin^+Kit^+ cells engrafted, whereas 2 of 3 mice transplanted with the $Mac1^+Gr1^+$ population engrafted (Figure 6B; Table 1). The pattern of engraftment (Figure 6B) was similar to that seen with *NHD13* Lin^+ cells shown in Figure 3E, with low level engraftment until week 30, followed by a brisk expansion, terminating in a lethal MDS or AML by week 50.

$B220^+Kit^+$ cells have previously been reported to function as progenitors with lymphoid and myeloid differentiation potential in *WT* mice (Balciunaite et al., 2005;

(C) Short-term (6 weeks) engraftment of primary (upper: LP BM n = 5, LN BM n = 6) or secondary (lower: LP BM n = 9, LN BM n = 14) recipients of *NHD13* LN or LP BM cells: *< p = 0.05; ***< p = 0.001.

(D) AML incidence in secondary recipients following transplantation. Results from three independent experiments. LP or LN-2° indicates secondary recipients of *NHD13* LP or LN BM, respectively.

(E and F) Summary of whole-exome sequencing (WES) results. The digits in the brackets indicate variant allele frequencies.

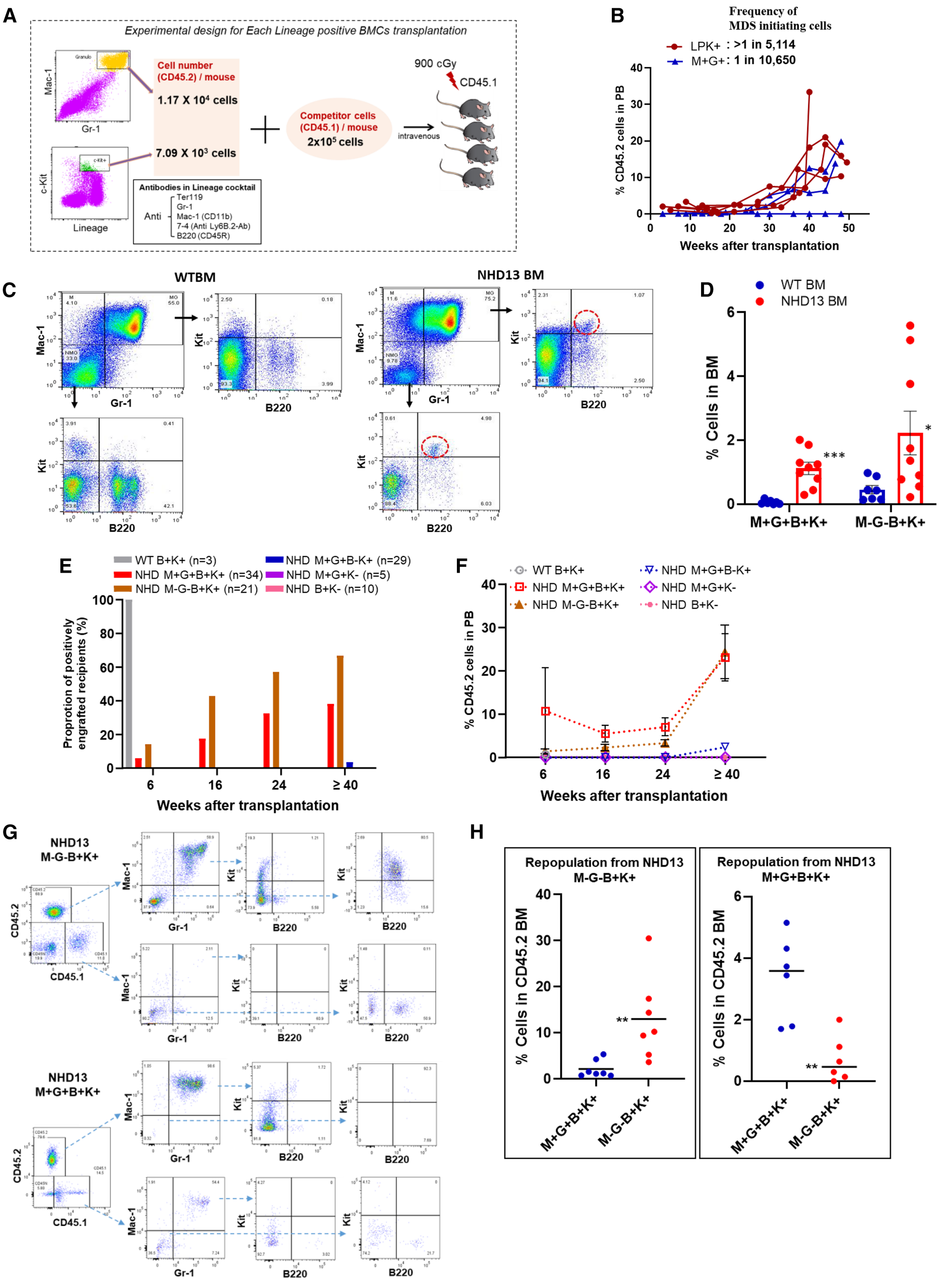

Figure 6. MDS-initiating cell populations in *NHD13* Lin+ BM
(A) Outline of the experiment.
(B) Engraftment kinetics and estimated frequency of MIC in each sorted cell population. LPK+, positive for lineage makers and Kit ($n = 4$); M+ G+, positive for both Mac1 and Gr1 ($n = 3$).

(legend continued on next page)

Kawaguchi, 2005; Ogawa et al., 2000). Given that self-renewal potential had been identified in $Mac1^+Gr1^+$ cells as well as Lin^+Kit^+ cells, we assessed the presence of $B220^+Kit^+$ cells within both the $Mac1^+Gr1^+$ and $Mac1^-$ $Gr1^-$ populations. Markedly increased numbers of $B220^+Kit^+$ cells were identified in *NHD13* BM compared to *WT* BM, among both the $Mac1^+Gr1^+$ and $Mac1^-$ $Gr1^-$ populations (Figures 6C and 6D). In order to assess the function of these *NHD13* populations ($Mac1^-$ $Gr1^-$ $B220^+Kit^+$ and $Mac1^+Gr1^+$ $B220^+Kit^+$) from 5-month-old mice (median age; Table S1), we tested the ability of these populations to engraft *WT* mice. $Mac1^+Gr1^+$ $B220^-$ Kit^+, $Mac1^+Gr1^+$ Kit^-, and $B220^+Kit^-$ cells from *NHD13* BM and $B220^+Kit^+$ cells from *WT* BM were used as additional controls (Figures 6E, 6F, and S5). Pooled engraftment results from seven independent experiments are summarized in Table S7. *WT* $B220^+Kit^+$ cells showed positive engraftment ($\geq$0.5% CD45.2 cells in PB) at post-transplant week 6 but no LT repopulating potential (Figures 6E, 6F, and S5). However, *NHD13* $B220^+Kit^+$ BM cells showed repopulation ability in both the $Mac1^+Gr1^+$ or $Mac1^-$ $Gr1^-$ recipient groups (Figures 6E, 6F, and S5), summarized in Table S7. Additionally, the *NHD13* $Mac1^-$ $Gr1^-$ $B220^+Kit^+$ cells repopulate and produce both $Mac1^+Gr1^+$ $B220^+Kit^+$ and $Mac1^-$ $Gr1^-$ $B220^+Kit^+$ cells in recipient mice along with multilineage potential, whereas $Mac1^+Gr1^+$ $B220^+Kit^+$ cells repopulate primarily with $Mac1^+Gr1^+$ $B220^+Kit^+$ cells and very few $Mac1^-$ $Gr1^-$ $B220^+Kit^+$ cells with lack of multilineage potential, suggesting that $Mac1^-$ $Gr1^-$ $B220^+Kit^+$ cells may be more immature than $Mac1^+Gr1^+$ $B220^+Kit^+$ cells (Figures 6G, 6H, S6A, and S6B). Taken together, these results reveal that an MIC is present in *NHD13* $B220^+Kit^+$ cells and that $Mac1^+Gr1^+$ $B220^+Kit^+$ cells from *NHD13* mice can self-renew for at least 56 weeks (Table S8).

We used WES to identify acquired mutations in MDS or AML that developed in mice transplanted with flow-sorted subpopulations (Table S9). Although minor clones within the donor population evolved into major clones in the recipients of sorted cells from 5205 to 5213 (marked by *Btbd18*/*Rapgef1* and *Ptk7*/*Tmem266* mutations, respectively), there were no common mutations in the recipients from donor 5141 or 5166.

Remarkably, 4 of 5 recipients of 5141 BM and 2 of 6 recipients of 5213 BM had acquired at least one independent truncation mutation of *Mn1* (Table S9), suggesting *in vivo* selection of clones that had acquired *Mn1* truncation mutations. All *Mn1* mutations encoded truncated versions of *Mn1*; there were no missense mutations. Moreover, 4 of the 7 independent *Mn1* mutations were clustered within a 6 bp “hotspot” (Table S9; Figure S6C). We identified two additional Mn1 truncation mutations that were associated with murine AML in the literature (Nofrini et al., 2011; Slape et al., 2007), both near amino acid (aa) 1240 of MN1. Including these two mutations, all nine of the MN1-truncating mutations retain the first 838 aa but the variations occur in the rest of 459 aa region (Figure S6C), suggesting an oncogenic effect of the truncated MN1. All these mutation analyses suggest that MICs may need to have additional relevant mutations for developing AML.

DISCUSSION

The incidence of MDS has long been known to increase with age, showing an exponential increase starting at age 60 (Maynadie et al., 1996). In this study, we show that this age-dependent disease can be recapitulated using the *NHD13* mouse model for MDS. Although young *NHD13* mice appear healthy, with normal PB counts and indices at 2 months of age, these mice invariably develop pancytopenia by 7 months of age. This decrease in hematopoietic output is reflected in the HSPC compartment; at 2 months of age, there was no difference in LSK number between *WT* and *NHD13* mice, while at 7 months of age, there was a 4.5-fold decrease in LSK cells in *NHD13* mice. Furthermore, *NHD13* BM showed a marked decrease in $CD41^-$ LT-HSCs, a population which is thought to reside at the apex of the HSC hierarchy (Bernitz et al., 2016; Gekas and Graf, 2013; Yamamoto et al., 2013).

Despite the clear loss of LSK and HSPC numbers, *NHD13* mice maintain PB complete blood counts (CBCs) that are only modestly decreased, and mice are generally asymptomatic until 8+ months of age. We considered the possibility that increased function of the *NHD13* HSPCs could

(C) Comparison of Mac1, Gr1, B220, and Kit staining of *WT* and *NHD13* BM cells.
(D) Proportion of M+G+B+K+ ($Mac1^+Gr1^+B220^+Kit^+$) and M−G−B+K+ ($Mac1^-Gr1^-B220^+Kit^+$) cells in *NHD13* (n = 9) and *WT* BM (n = 7): $^*< p = 0.05$; $^{***}< p = 0.001$.
(E) Each bar represents the proportion of recipient mice engrafted with CD45.2 cells ($\geq$0.5%) in PB at the indicated time. Results analysis from seven independent experiments.
(F) Mean percent engraftment of engrafted recipients at the indicated time. Each number of recipients is the same as indicated in the graph (E) legend. Results from seven independent experiments.
(G) Representative flow cytometry profiles for recipients of *NHD13* M−G−B+K and M+G+B+K+ cells.
(H) Summary of repopulation from *NHD13*: M+G+B+K+ n = 7, M−G−B+K+ n = 7 in the left; M+G+B+K+ n = 6, M−G−B+K+ n = 6 in the right; $^{**}< p = 0.01$.

Table 1. Frequency of an MDS-initiating cell among lineage-positive BM cells

NHD13 BM	Exp.	Cell dose for testing	No. of responders	No. of test	Estimated transplantable cell frequency	95% confidence interval
M+G+	LP	1.17×10^4	2	3	1/10,650	1/45,706–1/2,481
LPK+	LP	7.09×10^3	4	4	>1/5,114	1/17,400–1/1,503
M+G+B+K+	A	1,000	4	5	1/1,110	1/1,942–1/634
	B	1,000	1	4		
	C	597	2	5		
		199	3	5		
	D	200	0	10		
	E	1,000	3	5		
M−G−B+K+	A	3,500	2	2	1/899	1/1,607–1/502
	B	1,000	0	5		
	D	1,500	4	4		
		500	3	5		
	E	1,000	5	5		

M+, Mac1 positive; G+, Gr1 positive; M−, Mac1 negative; G−, Gr1 negative; B+, B220 positive; K+, Kit positive; LP, lineage positive. Exp. indicates the designation for independent experiments.

theoretically serve to maintain PB counts. However, CFC and HSCT assays indicated that *NHD13* HSPCs had decreased, as opposed to increased, functional output.

We finally considered the possibility that hematopoiesis in *NHD13* mice may be driven by Lin$^+$, as opposed to the expected Lin$^-$ cell population. In support of this hypothesis, *NHD13* Lin$^+$ cells express a stem cell module, including genes well known to be overexpressed in stem and progenitor cells, such as Hoxa7, Hoxa9, and Meis1. An *in vivo* HSCT assay supported this possibility, as approximately half of the mice transplanted with *NHD13* Lin$^+$ cells showed LT multilineage engraftment, whereas none of the mice transplanted with *WT* Lin$^+$ cells showed LT multilineage engraftment. In this context, it is important to note that chromatin immunoprecipitation experiments have shown that the NHD13 protein is bound to important upregulated target genes, such as *Hoxa*, *Hoxb*, and *Meis1* (Xu et al., 2016). This leads us to speculate that enforced expression of an NHD13 protein, driven by *Vav1* regulatory elements, enforces a self-renewal program in NHD13-committed progenitor cells.

The surprising finding that MDS could be transplanted by a Lin$^+$ cell led us to further refine the nature of an MIC. Recipients of Lin$^+$ BM cells produced very few LSK cells compared to recipients of Lin$^-$ BM, indicating that engraftment of the Lin$^+$ cells was not due to transplantation of contaminating Lin$^-$ cells, nor was it due to de-differentiation of Lin$^+$ cells to a Lin$^-$ population. In addition, secondary transplant experiments indicated that MDS could be transmitted by both Lin$^-$ and Lin$^+$ cells but that the pattern of engraftment differed between these two populations. Whereas engraftment of Lin$^-$ cells showed a rapid burst of engraftment, followed by a rapid decline and a subsequent terminal rise, engraftment of Lin$^+$ cells showed no rapid engraftment phase but instead an extended period (>26 weeks) of quiescence, followed by an eventual terminal rise in engraftment.

An MIC could be further refined based on the expression of the B220 and Kit cell surface markers. Over half of mice transplanted with B220$^+$Kit$^+$ cells showed LT engraftment, whereas <5% of mice transplanted with B220$^-$Kit$^+$ or B220$^+$Kit$^-$ cells showed LT engraftment. Of note, the frequency of B220$^+$Kit$^+$ cells was markedly increased in the BM of *NHD13* mice, which inevitably develop MDS (Lin et al., 2005; Slape et al., 2008), compared to *WT* mice. The B220$^+$Kit$^+$ cells were further fractionated using Mac1 and Gr1 surface markers using a limiting dilution assay. These experiments demonstrated that the estimated transplantable cell frequency of both Mac1$^+$Gr1$^+$ B220$^+$Kit$^+$ and Mac1$^-$ Gr1$^-$ B220$^+$Kit$^+$ cell populations was ~1/1,000, demonstrating that Mac1$^+$Gr1$^+$ cells, which are typically regarded as terminally differentiated, can self-renew and transmit MDS. These results are not entirely unexpected, as a prior study demonstrated that Mac1$^+$B220$^+$ BM cells

transduced with a PICALM::AF10 expression vector could self-renew as AML cells (Deshpande et al., 2006). These results, based on the *NHD13* mouse model, support recent findings that heavily mutated HSPCs from human patients with MDS retain the ability to both self-renew and differentiate into a spectrum of mature hematopoietic cell types (Rao, 2023; Schnegg-Kaufmann et al., 2023). In that study, the maturation of mutant HSPCs was more prominent following treatment with a DNA methyltransferase inhibitor, in at least a subset of patients (Schnegg-Kaufmann et al., 2023).

WES of AML or MDS cells from primary or secondary transplant experiments revealed several patterns of acquired mutations. In one example, a low VAF founder clone present in the primary transplant recipient became the predominant clone in AML that evolved in the secondary recipients. Of note, the secondary recipients all had unique mutations in genes encoding signaling proteins that are well known to be mutated in human AML (*Ptpn11*, *Nras*, *Kras*, *Cbl*, and *Kit*). These results, which show acquisition of mutations in signaling proteins as the disease evolves from MDS to AML, are similar to findings in human patients (Makishima et al., 2017; Sperling et al., 2017). An additional feature seen in a separate experiment was the acquisition of six different Mn1 mutations in recipients from two independent donors. All *Mn1* mutations encoded truncated versions of Mn1, suggesting that there was a strong *in vivo* selection for Mn1 mutations in the context of MDS caused by an *NHD13* transgene.

In summary, this study demonstrates that MDS progression in the *NHD13* mouse model is associated with a marked decrease in primitive HSPCs. This vacuum of normal hematopoiesis is then partially replaced by abnormal hematopoiesis originating from a multipotential B220$^+$ Kit$^+$ MIC. These data provide new insight into the pathogenesis and evolution of MDS and provide a foundation for the development of future therapeutic strategies.

METHODS

Mice

All mice for this study were generated and maintained on a C57BL/6 background. The *NHD13* transgenic mice were bred and maintained in NIH animal facilities. Two- or 7-month-old *NHD13* mice (CD45.2) were used for MDS hematopoiesis analysis. Eight- to 10-week-old C57BL/6 (CD45.1) recipient mice that were used for HSCT were purchased from either Charles River or Jackson Laboratory and maintained under micro-isolation conditions. All mouse handling and procedures were approved by the NCI Bethesda Animal Care and Use Committee (ACUC).

Flow cytometry

Antibodies for flow cytometry were obtained from either BD Pharmingen (BD), Thermo Fisher Scientific (TFS), BioLegend (BL), or SouthernBiotech (SB). Staining was performed in Hank's balanced salt solution (Ca^{2+}, Mg^{2+} free, Invitrogen, CA) containing 2% fetal bovine serum (FBS) for 30–60 min at 4°C. Combinations of antibodies and instruments used were as follows. For HSPC analysis, Lineage Biotin Antibody Cocktail (Miltenyi Biotec), Streptavidin-PerCP-Cy5.5 (TFS), CD117 (cKit)-APC-eFluor 780 (TFS), Sca-1-PE-Cy7 (TFS), CD150-APC (BL), CD135 (Flk2)-BV421 (BD), CD48-FITC (TFS), and DAPI (BD) were analyzed with a LSRFortessa instrument (BD Biosciences). For the evaluation of engraftment, Mac-1-PE (TFS), Gr-1-FITC (TFS), CD45.2-APC (BL), B220-PerCP-eFlour 710 (TFS), CD4-PerCP-Cy5.5 (BL), CD8-APC-Cy5.5 (SB), CD16/32 (clone 2.4G2, BD), and 7AAD were analyzed with a Northern Lights instrument (Cytek Biosciences). For committed lineage cell analysis, Mac-1-PE (TFS), Gr-1-FITC (TFS), CD71-PE/Dazzle594 (BL), B220-PerCP-eFlour 710 (TFS), CD19-Alexa Flour 700 (BL), CD4-PerCP-Cy5.5 (BL), CD8-APC-Cy5.5 (SB), Ter119-APC (BL), CD117 (cKit)-APC-eFluor 780 (TFS), and Sca-1-PE-Cy7 (TFS) were analyzed. CD45.1-Alexa Fluor 532 (TFS), CD45.2-PerCP (BL), and 7AAD were analyzed with a Northern Lights instrument (Cytek Biosciences).

Cell proliferation assay with BrdU

For cell proliferation assessment, mice were injected with 1 mg of BrdU intraperitoneally 48 h prior to euthanasia and BM harvest. BrdU and HSPC staining were performed using the manufacturer's suggested protocol and reagents (BrdU Flow Kit; BD Pharmingen).

HSCT procedure and engraftment evaluation

HSCT and engraftment assays were performed with minor modification of previously published procedures (Chung et al., 2018). In brief, BM cells (BMCs) from femora and tibiae of mice were stained with the antibody combinations as outlined in the text, and then collected using a MoFlo Astrios EQ (Beckman Coulter, IN) cell sorter. 1×10^3 to 2×10^4 sorted *NHD13* BMCs (CD45.2) were injected into lethally irradiated (900 cGy) WT recipient mice (CD45.1) via tail vein along with 2×10^5 BMCs from a non-irradiated healthy WT donor (CD45.1, used as competitor cells). For secondary transplantation, 1×10^6 of primary recipient BMCs per mouse were transplanted to lethally irradiated recipients.

Engraftment assays were carried out with PB obtained from the tail vein using di-potassium EDTA (ethylenediaminetetraacetic acid) salt as an anticoagulant. Each PB sample was separated into samples for flow cytometry and CBCs. CBCs were determined using a HEMAVET

Multispecies Hematology Analyzer (CDC Technologies, Oxford, CT). To evaluate engraftment level in tissues other than PB (typically BM or spleen), mice were euthanized, and tissues were harvested and placed into a single-cell suspension in Hank's balanced salt solution supplemented with 2% FBS (HF2) buffer.

Morphology of PB and BM was evaluated using May-Grünwald Giemsa staining of air-dried PB smear slides or BMC cytospin slides.

DNA extraction and PCR

Genomic DNA was extracted from PB or BMCs using the DNeasy Blood & Tissue kit (QIAGEN) and the manufacturer's suggested protocol. The *NHD13* transgene was amplified using primers 5′-TGGAGGGCCTCTTGGTACAG G-3′ (NUP98001) and 5′- GGCTTCTAAGCTGTCTGTGGC C-3′ (HOXD13-L1), and the program consisted of incubation at 95°C for 3 min, followed by 35 cycles of 95°C for 30 s, 62°C for 30 s, and 72°C for 30 s. Scid gene, as DNA quality control, was amplified using primers 5′-GGAA GAGTTTTGAGCAGACAATG-3′ (SCID A) and 5′- CATCAC AAGTTATAACAGCTGGG-3′ (SCID B), and the program consisted of incubation at 95°C for 3 min, followed by 35 cycles of 95°C for 30 s, 56°C for 30 s, and 72°C for 30 s.

GSEA and RT-qPCR

GSEA was performed with previously reported gene expression data (Novak et al., 2012) using the GSEA software (Subramanian et al., 2005). For RT-qPCR verification, complementary DNAs were synthesized using Superscript III Reverse Transcriptase (Invitrogen) with RNAs isolated by Trizol (Invitrogen) and the manufacture's protocol. RT-qPCR analysis was performed on a 7500 Fast RT-PCR system (Applied Biosystems, CA, USA) using the default thermal cycling conditions. 18S ribosomal RNA was used as an internal control. TaqMan primer and probe sets (Applied Biosystems) were used for Hoxa5 (Mm00439362_m1), Hoxa7 (Mm00657963_m1), Hoxa9 (Mm00439364), and Hoxa10 (Mm00433966).

CFC

CFCs were plated with 50–100 cells onto 35-mm Petri dishes in Methocult M3434 methylcellulose medium (STEMCELL Technologies, Canada) supplemented with cytokines (50 ng/mL recombinant mouse stem cell factor, 10 ng/mL rmIL-3, 10 ng/mL rhIL-6, and 3 U/mL rhEpo) and were incubated at 37°C in a 5% CO_2 incubator. The number of colonies was counted at day 10 after plating the cells.

WES capture and mutation analysis

Mouse Illumina DNA libraries were prepared and captured using the Agilent SureSelectXT Mouse All Exon Kit according to the manufacturer's instructions. In brief, DNA was fragmented on a Covaris S1 sonicator followed by end repair and phosphorylation. Blunt fragments were adenylated, ligated to Illumina Y-adapters, and PCR amplified. Bait hybridization proceeded for 48 h, followed by recovery of captured exome fragments by PCR. Captured exomes were sequenced on an Illumina HiSeq 2000.

Data processing and variant calling procedure mainly followed the Best Practices workflow recommended by the Broad Institute (http://www.broadinstitute.org/gatk/guide/best-practices). Briefly, the raw sequencing reads were mapped to mouse genome build 10 (mm10) by the Burrows-Wheeler Aligner (Li and Durbin, 2009), followed by local realignment using the GATK suite from Broad Institute, and duplicated reads were marked by Picard tools (http://picard.sourceforge.net).

Somatic variant calling was performed on data of tumor/normal paired samples by Mutect2 in GATK suit (https://gatk.broadinstitute.org/hc/en-us/articles/360037593851-Mutect2), and germline variant calling was done with the UnifiedGenotyper from the Broad Institute (https://www.broadinstitute.org/gatk/). SnpEff (Cingolani et al., 2012) and dbSNP 137 (NCBI) were used to annotate and predict effects of the variants.

The following filtering criteria were used for germline variation calls: (1) minimum read depth is 5; (2) minimum altered read number is 3; (3) minimum fraction of altered reads is 0.01; and (4) impact is "high" or "moderate."

The following filtering criteria were used for somatic variation calls: (1) minimum read depth is 5; (2) maximum altered read number in reference samples ("normal") is 2, or maximum fraction of altered reads in reference samples (normal) is 0.01; (3) minimum altered read number in samples with the fusion genes is 3 or minimum fraction of altered reads in tumor samples is 0.1; and (4) impact effect is high or moderate.

Statistics

Data are expressed as the mean ± standard errors of the mean or standard deviation where applicable. Differences between groups were analyzed by Student's t test.

RESOURCE AVAILABILITY

Lead contact

All other information and request for resources and reagents should be directed to the lead contact, Peter D. Aplan (aplanp@mail.nih.gov).

Materials availability

All materials used in this study are available from commercial vendors or from the authors.

Data and code availability

WES data are available in the sequence read archive under accession number, SRA BioProject ID: PRJNA1252163 (http://www.ncbi.nlm.nih.gov/bioproject/1252163) and PRJNA1256132 (http://www.ncbi.nlm.nih.gov/bioproject/1256132).

ACKNOWLEDGMENTS

The authors thank current and former members of the Aplan lab. We thank the NCI Sequencing Minicore for Sanger sequencing, the NCI Transgenic Core for generation of transgenic mice, the NCI Genomics Core for Next Generation Sequencing, the NCI Flow cytometry core for cell sorting, the NCI Pathology/Histotechnology Lab (PHL) for immunohistochemistry, and Maria Jorge for excellent animal husbandry. This work was supported by the Intramural Research Program of the National Cancer Institute, National Institutes of Health (grant nos. ZIA SC 010378 and BC 010983).

AUTHOR CONTRIBUTIONS

Y.J.C. designed and performed research, analyzed the data, and wrote the first draft of the manuscript; R.B. performed the experiments and analyzed the data; D.C. participated in the design of the study and data analysis; R.L.W. performed data curation, formal analysis, and investigation; Y.J.Z. performed data curation, formal analysis, validation, and visualization; P.M. designed research and analyzed the data; and P.D.A. designed research, analyzed the data, and wrote the final draft of the manuscript.

DECLARATION OF INTERESTS

P.D.A. receives royalties from the NIH Technology Transfer program for the invention of *NHD13* mice.

SUPPLEMENTAL INFORMATION

Supplemental information can be found online at https://doi.org/10.1016/j.stemcr.2025.102571.

Received: March 28, 2025
Revised: June 5, 2025
Accepted: June 6, 2025
Published: July 3, 2025

REFERENCES

Balciunaite, G., Ceredig, R., Massa, S., and Rolink, A.G. (2005). A B220+ CD117+ CD19- hematopoietic progenitor with potent lymphoid and myeloid developmental potential. Eur. J. Immunol. *35*, 2019–2030. https://doi.org/10.1002/eji.200526318.

Balderman, S.R., Li, A.J., Hoffman, C.M., Frisch, B.J., Goodman, A. N., LaMere, M.W., Georger, M.A., Evans, A.G., Liesveld, J.L., Becker, M.W., and Calvi, L.M. (2016). Targeting of the bone marrow microenvironment improves outcome in a murine model of myelodysplastic syndrome. Blood *127*, 616–625. https://doi.org/10.1182/blood-2015-06-653113.

Benito, A.I., Bryant, E., Loken, M.R., Sale, G.E., Nash, R.A., John Gass, M., and Deeg, H.J. (2003). NOD/SCID mice transplanted with marrow from patients with myelodysplastic syndrome (MDS) show long-term propagation of normal but not clonal human precursors. Leuk. Res. *27*, 425–436. https://doi.org/10.1016/S0145-2126(02)00221-7.

Bernitz, J.M., Kim, H.S., MacArthur, B., Sieburg, H., and Moore, K. (2016). Hematopoietic Stem Cells Count and Remember Self-Renewal Divisions. Cell *167*, 1296–1309.e10. https://doi.org/10.1016/j.cell.2016.10.022.

Bonnet, D., and Dick, J.E. (1997). Human acute myeloid leukemia is organized as a hierarchy that originates from a primitive hematopoietic cell. Nat. Med. *3*, 730–737. https://doi.org/10.1038/nm0797-730.

Cheng, G., Liu, F., Asai, T., Lai, F., Man, N., Xu, H., Chen, S., Greenblatt, S., Hamard, P.J., Ando, K., et al. (2017). Loss of p300 accelerates MDS-associated leukemogenesis. Leukemia *31*, 1382–1390. https://doi.org/10.1038/leu.2016.347.

Chung, Y.J., Choi, C.W., Slape, C., Fry, T., and Aplan, P.D. (2008). Transplantation of a myelodysplastic syndrome by a long-term repopulating hematopoietic cell. Proc. Natl. Acad. Sci. USA. *105*, 14088–14093. https://doi.org/10.1073/pnas.0804507105.

Chung, Y.J., Khawaja, G., Wolcott, K.M., and Aplan, P.D. (2018). Use of Hematopoietic Stem Cell Transplantation to Assess the Origin of Myelodysplastic Syndrome. J. Vis. Exp. *140*, e58140. https://doi.org/10.3791/58140.

Cingolani, P., Platts, A., Wang, L.L., Coon, M., Nguyen, T., Wang, L., Land, S.J., Lu, X., and Ruden, D.M. (2012). A program for annotating and predicting the effects of single nucleotide polymorphisms, SnpEff: SNPs in the genome of Drosophila melanogaster strain w(1118); iso-2; iso-3. Fly *6*, 80–92. https://doi.org/10.4161/fly.19695.

Deshpande, A.J., Cusan, M., Rawat, V.P.S., Reuter, H., Krause, A., Pott, C., Quintanilla-Martinez, L., Kakadia, P., Kuchenbauer, F., Ahmed, F., et al. (2006). Acute myeloid leukemia is propagated by a leukemic stem cell with lymphoid characteristics in a mouse model of CALM/AF10-positive leukemia. Cancer Cell *10*, 363–374. https://doi.org/10.1016/j.ccr.2006.08.023.

Gekas, C., and Graf, T. (2013). CD41 expression marks myeloid-biased adult hematopoietic stem cells and increases with age. Blood *121*, 4463–4472. https://doi.org/10.1182/blood-2012-09-457929.

Goldberg, L., Gough, S.M., Lee, F., Dang, C., Walker, R.L., Zhu, Y. J., Bilke, S., Pineda, M., Onozawa, M., Jo Chung, Y., et al. (2017). Somatic mutations in murine models of leukemia and lymphoma: Disease specificity and clinical relevance. Genes Chromosomes Cancer *56*, 472–483. https://doi.org/10.1002/gcc.22451.

Gough, S.M., Chung, Y.J., and Aplan, P.D. (2012). Depletion of cytotoxic T-cells does not protect NUP98-HOXD13 mice from myelodysplastic syndrome but reveals a modest tumor immunosurveillance effect. PLoS One *7*, e36876. https://doi.org/10.1371/journal.pone.0036876.

Hope, K.J., Jin, L., and Dick, J.E. (2004). Acute myeloid leukemia originates from a hierarchy of leukemic stem cell classes that differ in self-renewal capacity. Nat. Immunol. *5*, 738–743. https://doi.org/10.1038/ni1080.

Ito, C.Y., Li, C.Y.J., Bernstein, A., Dick, J.E., and Stanford, W.L. (2003). Hematopoietic stem cell and progenitor defects in Sca-1/Ly-6A-null mice. Blood *101*, 517–523. https://doi.org/10.1182/blood-2002-06-1918.

Kawaguchi, S. (2005). B-cell reconstitution by transplantation of B220 CD117 B-lymphoid progenitors into irradiated mice. Immunology *114*, 461–467. https://doi.org/10.1111/j.1365-2567.2005.02108.x.

Kerbauy, D.M.B., Lesnikov, V., Torok-Storb, B., Bryant, E., and Deeg, H.J. (2004). To the editor: Engraftment of distinct clonal MDS-derived hematopoietic precursors in NOD/SCID-beta 2-microglobulin-deficient mice after intramedullary transplantation of hematopoietic and stromal cells. Blood *104*, 2202–2203. https://doi.org/10.1182/blood-2004-04-1518.

Lapidot, T., Sirard, C., Vormoor, J., Murdoch, B., Hoang, T., Caceres-cortes, J., Minden, M., Paterson, B., Caligiuri, M.A., and Dick, J.E. (1994). A Cell Initiating Human Acute Myeloid-Leukemia after Transplantation into Scid Mice. Nature *367*, 645–648. https://doi.org/10.1038/367645a0.

Li, H., and Durbin, R. (2009). Fast and accurate short read alignment with Burrows-Wheeler transform. Bioinformatics *25*, 1754–1760. https://doi.org/10.1093/bioinformatics/btp324.

Lin, Y.W., Slape, C., Zhang, Z., and Aplan, P.D. (2005). NUP98-HOXD13 transgenic mice develop a highly penetrant, severe myelodysplastic syndrome that progresses to acute leukemia. Blood *106*, 287–295. https://doi.org/10.1182/blood-2004-12-4794.

Makishima, H., Yoshizato, T., Yoshida, K., Sekeres, M.A., Radivoyevitch, T., Suzuki, H., Przychodzen, B., Nagata, Y., Meggendorfer, M., Sanada, M., et al. (2017). Dynamics of clonal evolution in myelodysplastic syndromes. Nat. Genet. *49*, 204–212. https://doi.org/10.1038/ng.3742.

Maynadie, M., Verret, C., Moskovtchenko, P., Mugneret, F., Petrella, T., Caillot, D., and Carli, P.M. (1996). Epidemiological characteristics of myelodysplastic syndrome in a well-defined French population. Br. J. Cancer *74*, 288–290. https://doi.org/10.1038/bjc.1996.354.

Nilsson, L., Astrand-Grundström, I., Anderson, K., Arvidsson, I., Hokland, P., Bryder, D., Kjeldsen, L., Johansson, B., Hellström-Lindberg, E., Hast, R., and Jacobsen, S.E.W. (2002). Involvement and functional impairment of the CD34(+)CD38(-)Thy-1(+) hematopoietic stem cell pool in myelodysplastic syndromes with trisomy 8. Blood *100*, 259–267. https://doi.org/10.1182/blood-2001-12-0188.

Nimer, S.D. (2008). Myelodysplastic syndromes. Blood *111*, 4841–4851. https://doi.org/10.1182/blood-2007-08-078139.

Nofrini, V., Berchicci, L., La Starza, R., Gorello, P., Di Giacomo, D., Arcioni, F., Pierini, V., Crescenzi, B., Romoli, S., Matteucci, C., and Mecucci, C. (2011). MN1-ETV6 fusion gene arising from MDS with 5q. Leuk. Res. *35*, e123–e126. https://doi.org/10.1016/j.leukres.2011.03.019.

Novak, R.L., Harper, D.P., Caudell, D., Slape, C., Beachy, S.H., and Aplan, P.D. (2012). Gene expression profiling and candidate gene resequencing identifies pathways and mutations important for malignant transformation caused by leukemogenic fusion genes. Exp. Hematol. *40*, 1016–1027. https://doi.org/10.1016/j.exphem.2012.08.001.

Ogawa, M., ten Boekel, E., and Melchers, F. (2000). Identification of CD19(-)B220(+)c-Kit(+)Flt3/Flk-2(+)cells as early B lymphoid precursors before pre-B-I cells in juvenile mouse bone marrow. Int. Immunol. *12*, 313–324. https://doi.org/10.1093/intimm/12.3.313.

Rao, S. (2023). The kids are alright: MDS clones mature. Blood *141*, 1243–1245. https://doi.org/10.1182/blood.2022019187.

Raza-Egilmez, S.Z., Jani-Sait, S.N., Grossi, M., Higgins, M.J., Shows, T.B., and Aplan, P.D. (1998). NUP98-HOXD13 gene fusion in therapy-related acute myelogenous leukemia. Cancer Res. *58*, 4269–4273.

Rojas-Sutterlin, S., Lecuyer, E., and Hoang, T. (2014). Kit and Scl regulation of hematopoietic stem cells. Curr. Opin. Hematol. *21*, 256–264. https://doi.org/10.1097/moh.0000000000000052.

Schnegg-Kaufmann, A.S., Thoms, J.A.I., Bhuyan, G.S., Hampton, H.R., Vaughan, L., Rutherford, K., Kakadia, P.M., Lee, H.M., Johansson, E.M.V., Failes, T.W., et al. (2023). Contribution of mutant HSC clones to immature and mature cells in MDS and CMML, and variations with AZA therapy. Blood *141*, 1316–1321. https://doi.org/10.1182/blood.2022018602.

Shlush, L.I., Zandi, S., Mitchell, A., Chen, W.C., Brandwein, J.M., Gupta, V., Kennedy, J.A., Schimmer, A.D., Schuh, A.C., Yee, K.W., et al. (2014). Identification of pre-leukaemic haematopoietic stem cells in acute leukaemia (vol 506, pg 328, 2014). Nature *508*, 420. https://doi.org/10.1038/nature13190.

Slape, C., Hartung, H., Lin, Y.W., Bies, J., Wolff, L., and Aplan, P.D. (2007). Retroviral insertional mutagenesis identifies genes that collaborate with NUP98-HOXD13 during leukemic transformation. Cancer Res. *67*, 5148–5155. https://doi.org/10.1158/0008-5472.Can-07-0075.

Slape, C., Lin, Y.W., Hartung, H., Zhang, Z., Wolff, L., and Aplan, P.D. (2008). NUP98-HOX translocations lead to myelodysplastic syndrome in mice and men. J. Natl. Cancer Inst. Monogr., 64–68. https://doi.org/10.1093/jncimonographs/lgn014.

Spangrude, G.J., Heimfeld, S., and Weissman, I.L. (1988). Purification and characterization of mouse hematopoietic stem cells. Science *241*, 58–62. https://doi.org/10.1126/science.2898810.

Sperling, A.S., Gibson, C.J., and Ebert, B.L. (2017). The genetics of myelodysplastic syndrome: from clonal haematopoiesis to secondary leukaemia. Nat. Rev. Cancer *17*, 5–19. https://doi.org/10.1038/nrc.2016.112.

Subramanian, A., Tamayo, P., Mootha, V.K., Mukherjee, S., Ebert, B. L., Gillette, M.A., Paulovich, A., Pomeroy, S.L., Golub, T.R., Lander, E.S., and Mesirov, J.P. (2005). Gene set enrichment analysis: a knowledge-based approach for interpreting genome-wide

expression profiles. Proc. Natl. Acad. Sci. USA *102*, 15545–15550. https://doi.org/10.1073/pnas.0506580102.

Thanopoulou, E., Cashman, J., Kakagianne, T., Eaves, A., Zoumbos, N., and Eaves, C. (2004). Engraftment of NOD/SCID-beta 2 microglobulin null mice with multilineage neoplastic cells from patients with myelodysplastic syndrome. Blood *103*, 4285–4293. https://doi.org/10.1182/blood-2003-09-3192.

Xu, H., Valerio, D.G., Eisold, M.E., Sinha, A., Koche, R.P., Hu, W., Chen, C.W., Chu, S.H., Brien, G.L., Park, C.Y., et al. (2016). NUP98 Fusion Proteins Interact with the NSL and MLL1 Complexes to Drive Leukemogenesis. Cancer Cell *30*, 863–878. https://doi.org/10.1016/j.ccell.2016.10.019.

Yamamoto, R., Morita, Y., Ooehara, J., Hamanaka, S., Onodera, M., Rudolph, K.L., Ema, H., and Nakauchi, H. (2013). Clonal analysis unveils self-renewing lineage-restricted progenitors generated directly from hematopoietic stem cells. Cell *154*, 1112–1126. https://doi.org/10.1016/j.cell.2013.08.007.

Stem Cell Reports, Volume *20*

Supplemental Information

Disruption of normal stem cell function and transmission of myelodysplastic syndrome by self-renewal of committed myeloid lineage cells

Yang Jo Chung, Ryan Bertoli, Dengchao Cao, Robert L. Walker, Yuelin Jack Zhu, Paul Meltzer, and Peter D. Aplan

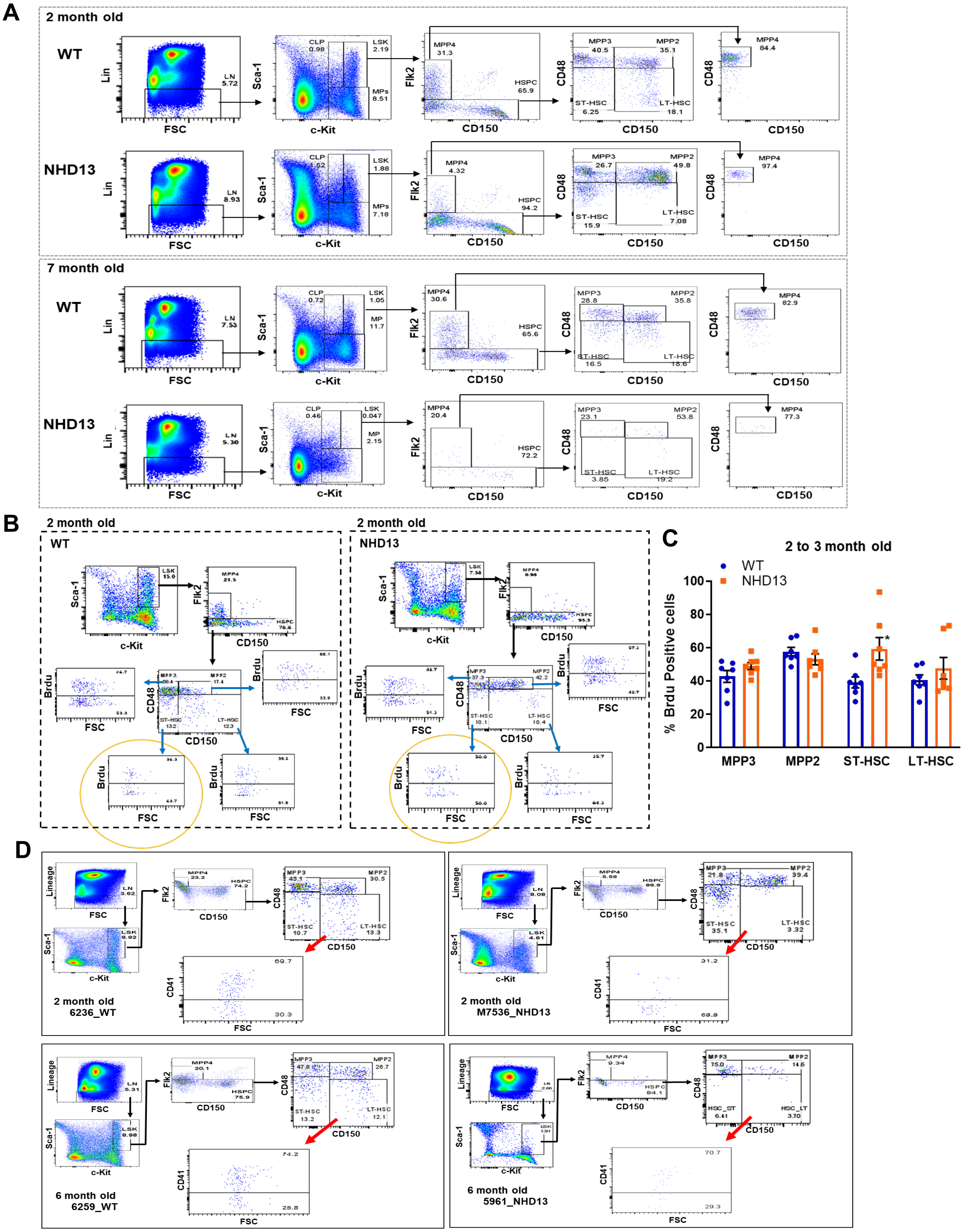

Supplementary figure S1. Hematopoietic stem and progenitor cell populations and proliferation in WT and NHD13 vary with age. (A) Representative flow cytometry profiles of BM HSPCs from age matched WT and NHD13 mice. **(B)** Representative flow cytometry profiles used to assess proliferation of HSPC in young (2-3 month) NHD13 bone marrow using Brdu incorporation. **(C)** Cell proliferation comparison of each LSK subset. Sample size for WT n=7, and for NHD13, n=7: * < p=0.05. **(D)** Representative flow cytometry profiles of HSCs on CD41 antigen expression from both genotype mice with differential age.

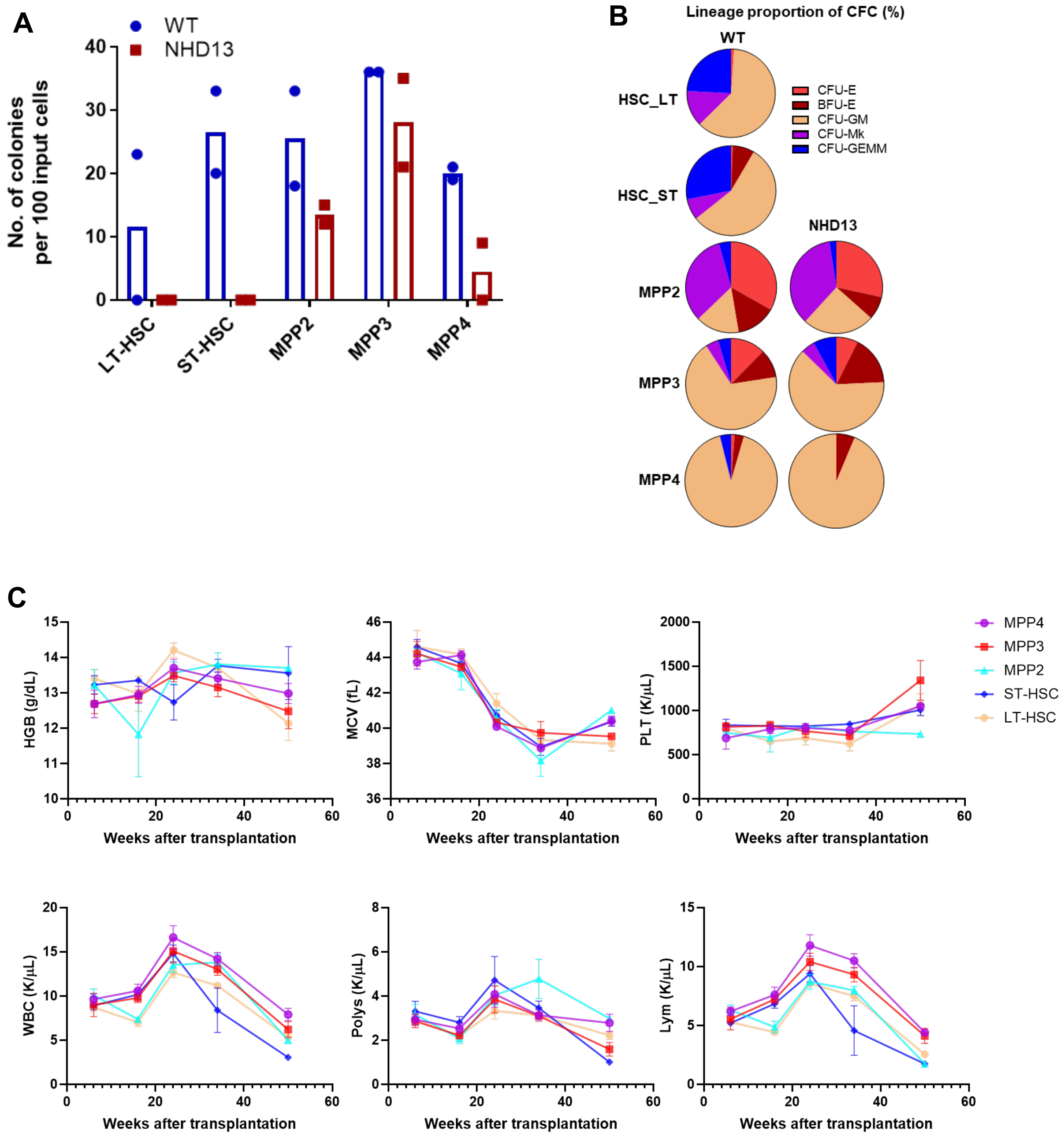

Supplementary figure S2. In vitro colony forming cell (CFC) assay and CBC from recipients of purified NHD13 HSPC. (A) Total number of colonies from each group assessed 10 days after plating. The data from the results of two independent experiments. **(B)** Lineage analysis of colonies from each HSPC population, based on colony morphology. The data from the results of two independent experiments. **(C)** HGB, hemoglobin; MCV, mean corpuscular volume; PLT, platelet; WBC, white blood cell; Polys, neutrophile cells; Lym, lymphoid cells. Recipient mice of each HSPC subset, n=5.

A

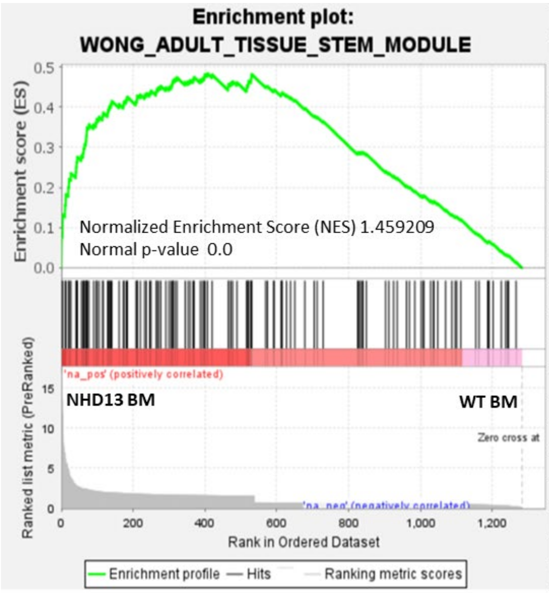

	SYMBOL	RANK IN GENE LIST	RANK METRIC SCORE	RUNNING ES	CORE ENRICHMENT
1	GRIA2	1	16.691	0.0782	Yes
2	HOXA9	4	11.682	0.1318	Yes
3	HOXA5	11	6.207	0.1560	Yes
4	PBX3	13	5.779	0.1825	Yes
5	SOCS2	20	4.800	0.2001	Yes
6	HOXC4	22	4.531	0.2207	Yes
7	PTGS2	27	3.799	0.2352	Yes
8	HOXA3	41	2.808	0.2372	Yes
9	LIMCH1	42	2.769	0.2504	Yes
10	P2RY14	43	2.762	0.2634	Yes
11	SERPINH1	45	2.717	0.2754	Yes
12	CTSL	56	2.475	0.2785	Yes
13	HOXA10	61	2.418	0.2865	Yes
14	GJA1	63	2.398	0.2970	Yes
15	DNTT	65	2.388	0.3074	Yes
16	ID4	69	2.350	0.3160	Yes
17	TNC	70	2.348	0.3271	Yes
18	MYCN	71	2.346	0.3382	Yes
19	NID1	72	2.335	0.3493	Yes
20	SELP	77	2.261	0.3565	Yes

B

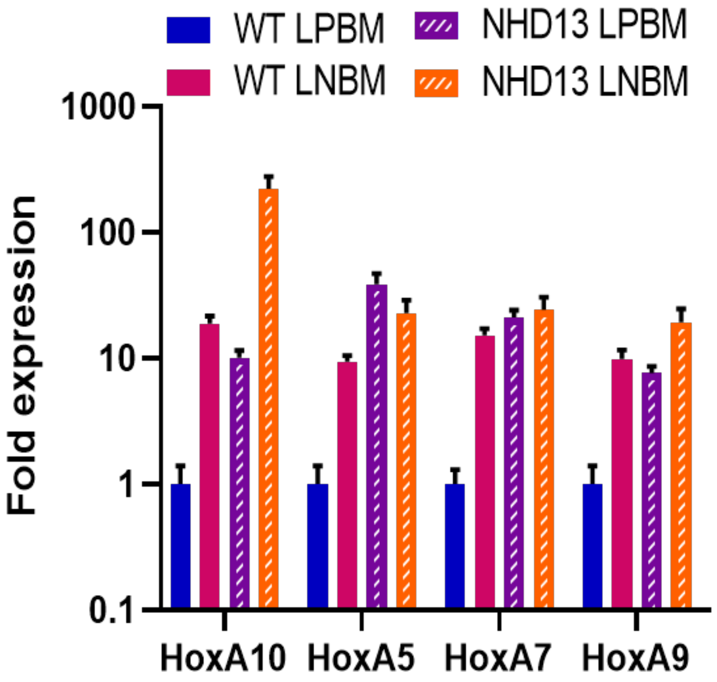

Supplementary figure S3. Gene expression analysis of NHD13 mice vs WT. (A) Gene Set Enrichment Assay with microarray DATA of NHD13 BM vs WT. **(B)** Expression analysis of genes related with HSC self-renewal using RQ-PCR with fractionated BM in terms of lineage positive (LP) antigens or negative (LN) from each genotype of mice. Sample size for WT n=3, and for NHD13 n=3.

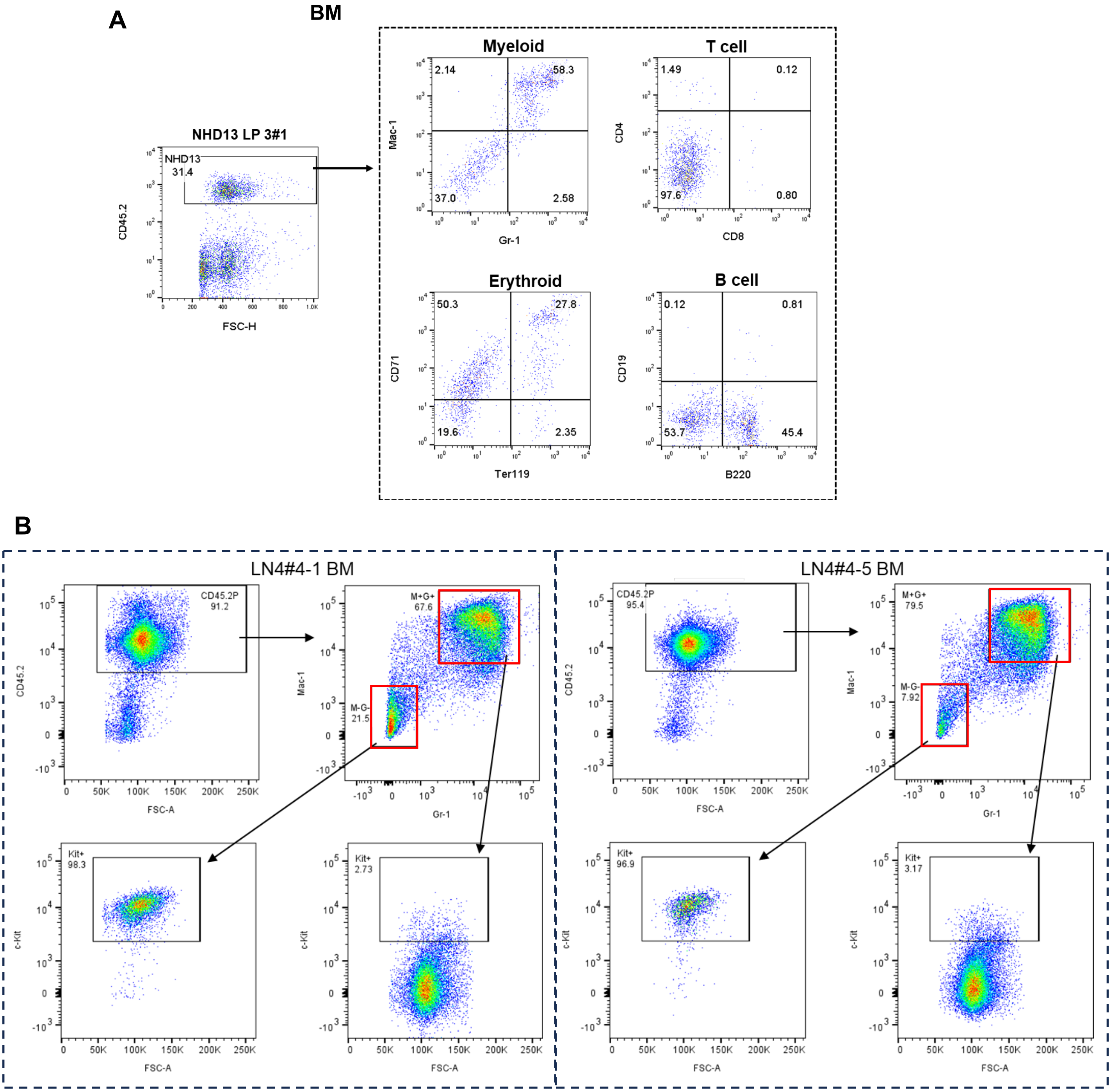

Supplementary figure S4. Flow cytometry profiles demonstrate multi-lineage potential of NHD13 LP BM cells and AML transformation. (A) Representative flow cytometry profiles demonstrate engraftment of Mac1$^+$Gr1$^+$ (myeloid), CD71$^+$Ter119$^+$ (erythroid), CD4$^+$, CD8$^+$ (T-lymphoid), CD19$^+$B220$^+$ (B-lymphoid) cells at 59 weeks post HSCT. **(B)** M+G+ indicates double positive of Mac1 and Gr1. M-G-, double negative of Mac1 and Gr1 antigens. Red line boxes indicate sorted population for the WES analysis.

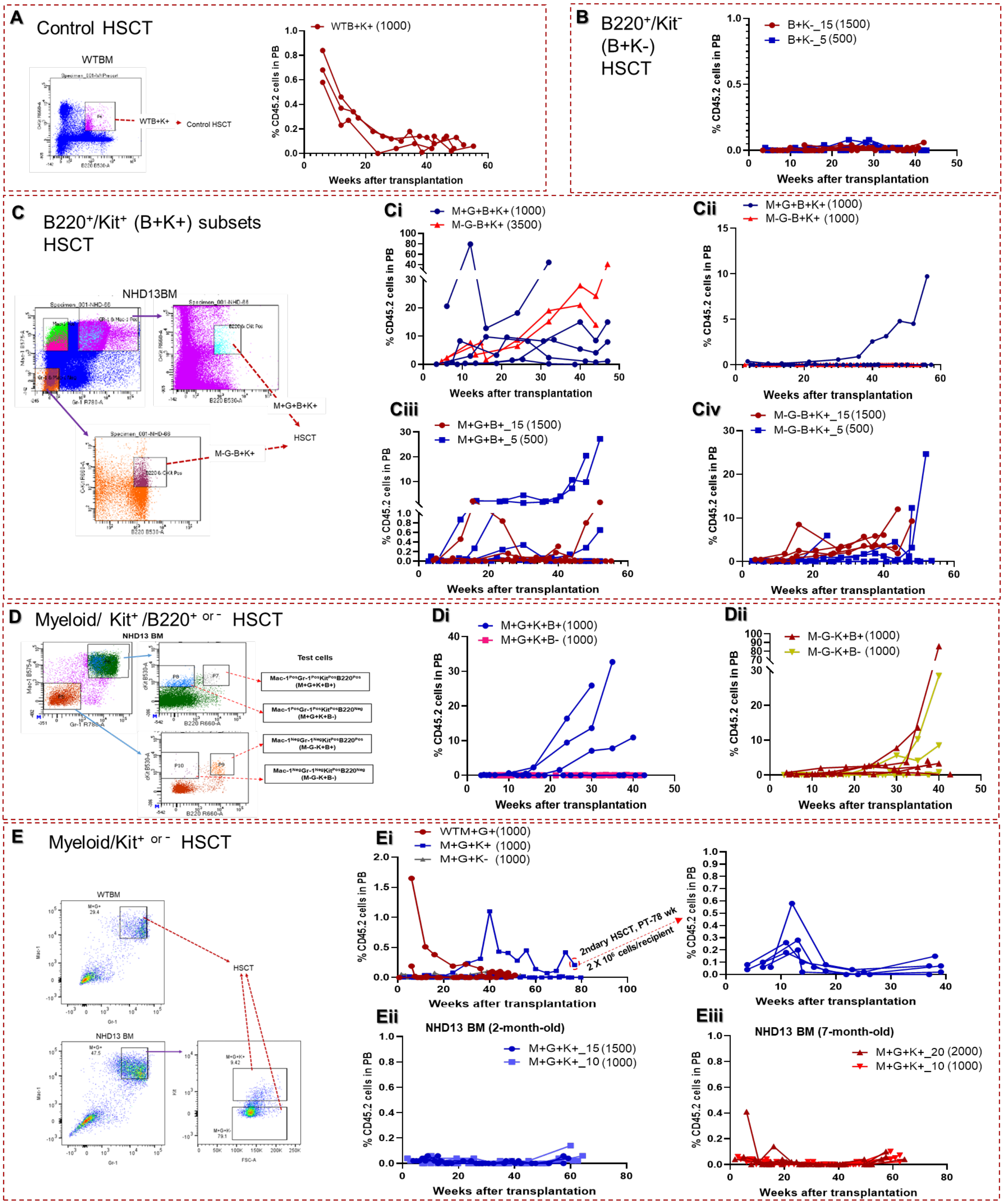

Supplementary figure S5. MDS initiating cell (MIC) may exist in B220 and Kit double positive (B+K+) cells of NHD13 LPBM cells. (A) Control HSCT with WT B+K+ cells. **(B)** NHD13 B220^{+}/Kit^{-} cells transplants. **(C)** HSCTs for NHD13 B+K+ cells with or without myeloid markers. Each graph indicates independent experiments. **(D)** HSCT for testing NHD13 Kit^{+}/B220$^{+\ or\ -}$ cells with (Di) or without (Dii) myeloid markers. **(E)** Evaluation of NHD13 Kit$^{+\ or\ -}$ cells with myeloid makers. Each roman numberings indicate independent experiments. In figure legends, upper case letters mean antigen positive and lower case, antigen negative. The numbers in the parenthesis next to the legend indicate cell numbers transplanted to given recipients in HSCT assay.

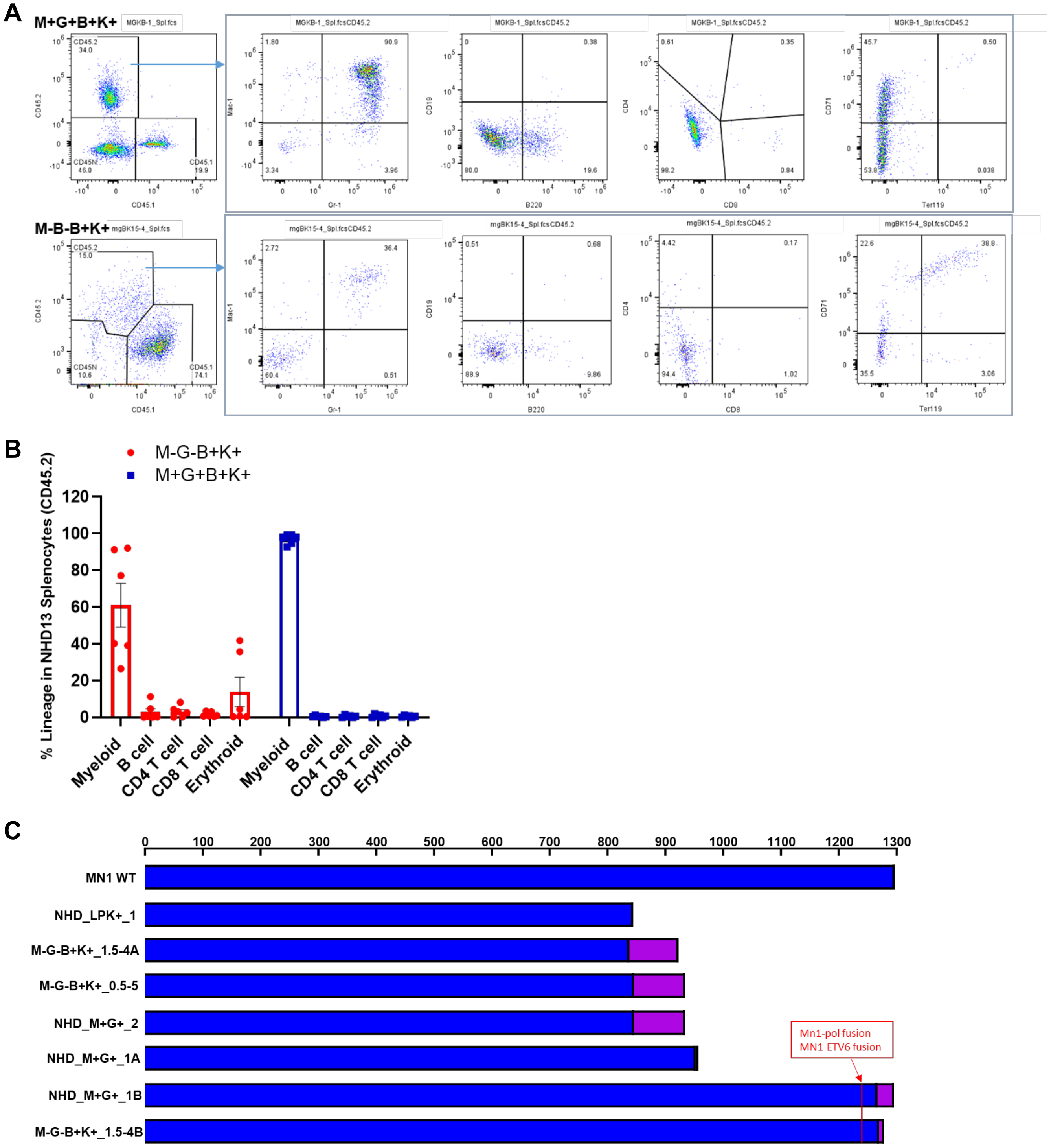

Supplementary figure S6. NHD13 B220 and Kit (B+K+) double positive cells have multilineage potential and frequently acquire Mn1 mutations. (A) Representative flow cytometry profiles demonstrate engraftment of Mac1^{+}Gr1^{+} (myeloid), CD71^{+}Ter119^{+} (erythroid), CD4^{+}, CD8^{+} (T-lymphoid), CD19^{+}B220^{+} (B-lymphoid) cells in splenocytes at 30- or 48-weeks post HSCT. **(B)** Summary of differentiated progeny of the NHD13 BK cells in the spleens of recipient mice. Sample size for M-G-B+k+, n=6, and for M+G+B+K+, n=6. Results from 4 independent experiments. **(C)** Mn1 mutant proteins were deduced from the mutation sequence. Amino acid position 1240 of MN1 protein have been reported as a fusion site of Mn1-pol fusion (Cancer Res 2007;67:(11)) and MN1-ETV6 fusion (Leuk Res 2011;35) .

Supplementary Table S1. Meta data of primary donors in this study.

Experiments (HSCT type)	Mouse ID	GT	Age (mo)	HGB (g/dL)	MCV (fL)	PLT (K/uL)	WBC (K/uL)	Polys (K/uL)	Lym (K/uL)	Mono (K/uL)
1st Exp. (LSK)	17	WT	9	na	na	na	na	na	na	na
	18	NHD13	9	na	na	na	na	na	na	na
2nd Exp. (LSK)	67	WT	7	17.0	43.6	1412	10.44	3.08	6.70	0.60
	76	NHD13	6	13.6	51.2	476	2.90	1.45	1.19	0.24
	77	NHD13	6	11.9	60.0	750	2.68	1.17	1.26	0.23
1st Exp. (LSK subset)	543	NHD13	3	13.4	49.5	760	3.80	1.44	2.01	0.34
2nd Exp. (LSK subset)	547	NHD13	4	13.3	48.4	673	3.28	1.73	1.25	0.29
2nd Exp. (Lin^{+} or Lin^{-})	5969	WT	6	12.8	39.0	1044	6.20	1.54	4.27	0.38
	5970	NHD13	6	11.9	45.8	472	2.82	1.65	0.88	0.25
3rd Exp. (Lin^{+} or Lin^{-})	7645	WT	3	15.3	40.8	1119	9.34	2.29	6.26	0.62
	7640	NHD13	4	12.5	42.9	595	1.78	0.75	0.85	0.17
4th Exp. (Lin^{+} or Lin^{-})	7687	WT	5	14.1	41.0	1214	19.2	5.82	11.72	1.31
	7688	NHD13	5	11.6	46.3	549	2.64	1.64	0.87	0.12
5th Exp. (Lin^{+} or Lin^{-})	7726	WT	5	na	na	na	na	na	na	na
	7735	NHD13	5	11.5	44.6	616	1.96	0.8	1.01	0.13
2nd Exp. (Lin^{+} fraction)	5141	NHD13	5	11.9	45.4	647	3.46	1.74	1.38	0.32
3rd Exp. (Lin^{+} fraction)	5165	WT	6	12.5	41.2	1635	6.84	0.63	5.74	0.47
	5166	NHD13	6	10.2	48.5	784	3.28	1.60	1.46	0.23
4th Exp. (Lin^{+} fraction)	5175	WT	5	13.4	42.0	1068	11.50	1.81	8.97	0.71
	5178	NHD13	5	11.9	45.9	627	2.80	1.29	1.35	0.15
5th Exp. (Lin^{+} fraction)	5197	WT	4	14.6	42.0	959	13.68	2.07	10.27	1.03
	5195	NHD13	5	11.9	47.3	616	3.08	1.37	1.43	0.27
6th Exp. (Lin^{+} fraction)	5205	NHD13	6	12.6	46.6	606	3.68	1.90	1.47	0.30
7th Exp. (Lin^{+} fraction)	5213	NHD13	4	12.3	46.6	598	2.98	1.60	1.17	0.20
8th Exp. (Lin^{+} fraction)	5223	NHD13	7	11.3	44.9	515	2.04	1.44	0.35	0.23
	5240	NHD13	2	14.4	42.1	365	4.44	1.11	2.84	0.49
9th Exp. (Lin^{+} fraction)	5282	NHD13	6	10.9	48.9	1535	3.40	1.66	1.36	0.37

na means not applicable.

Supplementary Table S2. Incidence of myeloid engraftment (>1%) in recipient mice (Engrafted mice/Transplanted mice).

Experiments	NHD Lin$^-$	NHD Lin$^+$	WT Lin$^-$	WT Lin$^+$
2nd Exp.	1/3	1/2	4/4	0/4
3rd Exp.	0/3	2/2	1/1	0/3
4th Exp.	2/4	1/5	4/4	0/4
5th Exp.	3/3	1/3	4/4	0/3
Total	6/13 (46%)	5/12 (42%)	13/13 (100%)	0/14 (0%)

Supplementary Table S3. Endpoint data from primary NHD13 recipients

Donor cells of HSCT	Recipient ID	Follow-up (*weeks)	CBC Acquisition (*week)	WBC (K/uL)	ANC (K/uL)	PLT (K/uL)	HGB (g/dL)	MCV (fL)	CD45.2P in BM (%)	Cause of Death	Cell dose per 2° recipient	Diagnosis
Lin+ (LP) BM	LP2#2	32	24	8.34	1.79	609	12.6	45.5	na	Infection (penile wound)	na	MDS
	**LP3#1**	**59**	59	4.99	0.83	1069	13.9	54.6	24.1	**Donor for secondary BMT**	**2.41E+05**	MDS
	**LP3#2**	**59**	59	2.36	0.53	530	13.0	54.7	14.5	**Donor for secondary BMT**	**1.45E+05**	MDS
	**LP4#3**	**47**	46	6.36	2.26	650	13.3	51.8	33.6	**Donor for secondary BMT**	**3.36E+05**	MDS
	LP5#1	78	78	12.26	4.15	811	11.5	48.6	na	Found Dead	na	MDS
Lin$^-$ (LN) BM	LN2#2	58	53	4.98	1.36	1096	11.5	44.9	50	Euthanasia at the endpoint	na	MDS
	**LN4#1**	**58**	58	13.98	6.36	721	12.8	46.5	44.7	**Donor for secondary BMT**	**4.47E+05**	MDS
	**LN4#4**	**58**	58	6.82	1.95	186	10.3	47.6	51.8	**Donor for secondary BMT**	**5.18E+05**	MDS
	LN5#1	59	59	1.78	1.05	259	11.1	53.6	58.9	Euthanasia at the endpoint	na	MDS
	**LN5#2**	**41**	40	6.04	3.07	445	11.0	51.4	43.9	**Donor for secondary BMT**	**4.39E+05**	MDS
	LN5#3	54	54	11.16	1.72	414	8.7	69.2	na	Found Dead	na	MDS

*Weeks post HSCT; na, not applicable; Bold letters mean data of primary recipients used as secondary donors.

Supplementary Table S4. Endpoint data for secondary recipients.

Recipient ID	Follow-up (*weeks)	CBC Acquisition (*week)	WBC (K/uL)	Polys (K/uL)	PLT (K/uL)	HGB (g/dL)	MCV (fL)	Blast count (% BMC)	BM Engraftment (% CD45.2)	PB Engraftment (% CD45.2)	Cause of Death	Comments
LP3#1-**1**	24	24	2.08	0.64	95	1.7	65.1	NA	NA	0.00	Moribund	NHD13 cells engraftment not detected; Blood collection near portal vein.
LP3#1-**2**	31	24	9.26	1.94	654	13.5	46.9	NA	NA	0.14	Unknown	
LP3#1-**3**	34	24	4.52	0.83	609	11.5	47.5	NA	NA	0.60	Unknown	
LP3#2-**2**	59	59	1.94	1.11	360	9.7	53.1	18.1	4.15	0.43	MDS	
LP3#2-**3**	50	50	29.92	10.12	298	8.2	59.6	NA	91.5	79.79	AML	
LP3#2-**4**	59	59	12.58	4.46	474	8.3	56.2	20.7	71.6	82.01	AML	
LP3#2-**5**	43	43	2.6	0.68	630	9.7	54.0	14.5	75.5	54.59	MDS	
LP4#3-**1**	53	53	10.56	3.73	371	7.7	69.3	42.8	94.3	55.35	AML	
LP4#3-**2**	70	70	9.00	5.92	770	12.2	45.1	11.5	54.7	20.16	MDS	
LP4#3-**3**	70	70	3.72	1.66	642	11.9	48.7	25.6	70.1	17.91	AML	
LP4#3-**4**	39	39	57.48	15.52	61	3.2	92.6	NA	NA	90.11	AML	
LN4#1-**1**	53	53	17.96	7.65	212	8.7	56.6	31.3	94.5	77.57	AML	
LN4#1-**2**	53	53	4.06	0.67	381	9.4	53.8	37.9	69.1	42.14	AML	
LN4#1-**3**	44	44	14.12	3.03	98	3.9	82.0	68.5	63.3	52.28	AML	
LN4#1-**4**	6	6	6.98	2.67	504	13.2	47.5	NA	NA	NA	Iatrogenic	Experimental procedure error
LN4#1-**5**	53	53	5.78	2.54	249	4.9	55.8	32.9	70.3	49.61	AML	
LN4#4-**1**	30	30	28.9	3.22	613	7.8	66.8	52.5	80.9	84.20	AML	
LN4#4-**2**	24	24	26.04	5.53	292	10.8	47.1	20.0	88.7	52.17	AML	
LN4#4-**3**	24	24	18.98	2.92	469	9.2	61.1	28.8	84.6	27.11	AML	
LN4#4-**4**	24	24	20.38	5.00	317	9.6	60.1	20.1	88.4	41.24	AML	
LN4#4-**5**	30	30	28.8	6.86	169	9.8	63.3	38.7	93.1	69.81	AML	
LN5#2-**1**	48	48	1.66	1.12	420	11.4	52.5	11.6	60.5	45.59	MDS	
LN5#2-**2**	48	48	3.04	1.39	611	14.7	45.2	12.6	NA	NA	Euthanized	NHD13 cells engraftment not detected at the time of euthasia
LN5#2-**3**	48	45	5.2	2.18	662	10.4	44.2	NA	NA	NA	Unknown	NHD13 cells engraftment not detected at post-transplanation 45 week in PB
LN5#2-**4**	38	38	3.9	2.1	154	3.0	48.2	36.9	23.0	0.4	AML	

*Weeks post HSCT

LP, lineage positive cells from primary donor; LN, lineage negative cells from primary donor

Supplementary Table S5. Acquired mutations in recipient mice transplanted with Lin$^-$ (LN) or Lin$^+$ (LP) cells from NHD13 BM.

ID	Experiment	Donor	Recipient Type	CD45.2 Purity (%)	Dagnosis	Gene	Nucleotide	Amino Acid	VAF
LN4#4	4th	NHD13 (7688) Lineage Negative BM cells	Primary	51.8	MDS	Nfic	1176_1181 dupGGCCAT	A393_I394 insMA	0.158
						Tlr5	124G>A	G42S	0.118
						Spag8	1028delG	R343fs	0.176
						Ptprv	2744T>C	V915A	0.571
						Stxbp6	527G>A	R176H	0.357
						Slc17a4	532G>T	G178C	0.364
						Dpp9	37G>A	A13T	0.250
						Vwa2	112G>A	A38T	0.750
						Fam110a	461delG	R154fs	0.400
						Abca14	1810G>C	N604H	0.412
LN4#4-1_MG	4th	LN4#4 recipient WBM cells	Secondary	98.4 (sorted BM, Mac1$^+$ Gr-1$^+$ Kit$^-$)	AML	Ptpn11	205G>A	E69K	0.375
						Kras	34G>A	G12S	0.148
						Nfic	1176_1181 dupGGCCAT	A393_I394 insMA	0.429
						Tlr5	124G>A	G42S	0.667
						Tigit	395T>A	V132E	0.667
						Spag8	1028delG	R343fs	0.409
						Pnpla8	860G>A	R287H	0.310
LN4#4-1_Kit	4th	LN4#4 recipient WBM cells	Secondary	99.8 (sorted BM, Mac1$^-$ Gr1$^-$ Kit$^+$)	AML	Ptpn11	205G>A	E69K	0.214
						Kras	34G>A	G12S	0.250
						Nfic	1176_1181 dupGGCCAT	A393_I394 insMA	0.533
						Tlr5	124G>A	G42S	0.448
						Tigit	395T>A	V132E	0.625
						Spag8	1028delG	R343fs	0.464
						Pnpla8	860G>A	R287H	0.256
						Lhb	401A>C	H134P	0.304
						Fank1	737C>T	T246M	0.273
LN4#4-2	4th	LN4#4 recipient WBM cells	Secondary	89.9	AML	Kit	2472T>G	N824K	0.913
						Nfic	1176_1181 dupGGCCAT	A393_I394 insMA	0.111
						Tlr5	124G>A	G42S	0.500
						Tigit	395T>A	V132E	0.462
						Spag8	1028delG	R343fs	0.448
LN4#4-3	4th	LN4#4 recipient WBM cells	Secondary	86.4	AML	Nfkb2	1880A>G	E627G	0.389
						Nfic	1176_1181 dupGGCCAT	A393_I394 insMA	0.346
						Tlr5	124G>A	G42S	0.371
						Tigit	395T>A	V132E	0.400
						Spag8	1028delG	R343fs	0.361
						Sft2d2	252G>A	M84I	0.235
LN4#4-4	4th	LN4#4 recipient WBM cells	Secondary	89.8	AML	Cbl	1090-2A>T		0.500
						Nfic	1176_1181 dupGGCCAT	A393_I394 insMA	0.643
						Tlr5	124G>A	G42S	0.656
						Tigit	395T>A	V132E	0.444
						Spag8	1028delG	R343fs	0.609
LN4#4-5_MG	4th	LN4#4 recipient WBM cells	Secondary	98.9 (sorted BM, Mac1$^+$ Gr-1$^+$ Kit$^-$)	AML	Nras	34G>T	G12C	0.500
						Nfic	1176_1181 dupGGCCAT	A393_I394insMA	0.364
						Tlr5	124G>A	G42S	0.313
						Tigit	395T>A	V132E	0.667
						Spag8	1028delG	R343fs	0.556
						Kif21a	2555G>A	R852Q	0.300
						Tgm6	1432G>A	V478M	0.500
						Slco1a4	862G>A	E288K	0.485
						Tex264	461C>A	A154D	0.667
LN4#4-5_Kit	4th	LN4#4 recipient WBM cells	Secondary	99.8 (sorted BM, Mac1$^-$ Gr1$^-$ Kit$^+$)	AML	Nras	34G>T	G12C	0.506
						Nfic	1176_1181 dupGGCCAT	A393_I394 insMA	0.462
						Tlr5	124G>A	G42S	0.648
						Tigit	395T>A	V132E	0.500
						Spag8	1028delG	R343fs	0.420
						Kif21a	2555G>A	R852Q	0.692
						Tgm6	1432G>A	V478M	0.429
						Slco1a4	862G>A	E288K	0.377
						Actb	850A>G	K284E	0.367
						Foxp1	1315A>C	T439P	0.225
LP3#2-3	3rd	LP3#2 recipient WBM cells (LP3#2 is a primary recipient transplanted with Lineage Positive BM cells of NHD13 (7640) mouse)	Secondary	93.2	AML	Etv6	592delG	E198fs	0.684
						Trp53	808C>T	R270C	0.389
						Kras	38G>A	G13D	0.276
						Mina	652G>A	G218S	0.389
						Mob3a	332C>T	T111M	0.655
						Ldah	562C>T	R188*	0.472
						Prl5a1	284T>C	V95A	0.455
						Thoc3	374G>A	R125H	0.286
						Ptprg	2914A>G	N972D	0.615
						Fam110a	34G>C	A12P	0.667
						Taf13	238C>T	R80*	0.421
						Hyal5	1288T>C	S430P	0.435
LP3#2-5	3rd	LP3#2 recipient WBM cells (LP3#2 is a primary recipient transplanted with Lineage Positive BM cells of NHD13 (7640) mouse)	Secondary	72.5	MDS	Jarid2	1160_1161 insGG	K388fs	0.455
						Mina	652G>A	G218S	0.500
						Ndrg2	718dupC	R240fs	0.435
						Pyroxd2	564G>T	Q188H	0.375
						Rbm20	2554G>A	G852R	0.300
						Hyal5	1288T>C	S430P	0.348
						Col5a3	2066C>T	P689L	0.257
						Sidt2	1429A>T	I477F	0.231

Red letters indicate mutations associated with hematologic malignancy. Blue letters highlight mutations present in donor BM.

Supplementary Table S6. Total time from primary transplant to AML in secondary recipients.

Primary recipients	Weeks (Post-BMT)	Secondary recipients	Weeks (Post-BMT)	Total Weeks	Total Months
LP3#2	59	LP3#2-2	59	118	29.5
		LP3#2-3	50	109	27.3
		LP3#2-4	59	118	29.5
		LP3#2-5	43	102	25.5
LP4#3	46	LP4#3-1	53	99	24.8
		LP4#3-2	70	116	29.0
		LP4#3-3	70	116	29.0
		LP4#3-4	39	85	21.3
Mean					**27.0**
SD					**3.0**

Supplementary Table S7. Engraftment of lineage positive NHD13 sub-populations.

	B220+ Kit+ cells		Kit+ B220- cells		Kit- B220- cells	
	Mice tested	Mice engrafted	Mice tested	Mice engrafted	Mice tested	Mice engrafted
	55	28	29	1	15	0
Ratio		0.51		0.03		0.00

	M+G+B+K+ cells	
	Mice tested	Mice engrafted
	34	13
Ratio		0.38

	M-G-B+K+ cells	
	Mice tested	Mice engrafted
	21	14
Ratio		0.67

M+, Mac1 positive; G+, Gr1 positive; M-, Mac1 negative; G-, Gr1 negative; B+, B220 positive; K+, Kit positive. B220+Kit+ cells were further separated using Mac1 and Gr1 antibodies into M+G+B+K+ and M-G-B+K+ populations.

Supplementary Table S8. Endpoint data for recipients of isolated NHD13 sub-populations.

Exp.	Recipient ID	Follow-up (*weeks)	WBC (K/uL)	Poly (K/uL)	PLT (K/uL)	HGB (g/dL)	MCV (fL)	Blast count (%BMC)	BM Engraftment (%CD45.2)	Cause of Death
2nd	NHD LPK+_1	48	1.80	0.57	413	11.2	49.0	18.7	75.0	MDS
	NHD LPK+_2	48	2.44	0.87	662	11.7	48.6	13.0	76.7	MDS
	NHD LPK+_3	48	4.22	0.75	509	6.9	56.9	36.1	38.5	AML
	NHD LPK+_4	40	4.96	1.49	457	9.8	46.7	33.5	89.3	AML
	NHD M+G+_1	48	7.32	3.63	1019	12.3	43.9	17.4	48.1	MDS
	NHD M+G+_2	48	2.54	0.73	813	8.2	49.7	15.3	53.1	MDS
3rd	M+G+B+K+_1	47	8.5	5.26	1072	10.1	45.8	18.8	79.6	MDS
	M+G+B+K+_2	47	10.16	4.11	1043	11.1	40.7	10.6	10.9	MDS
	M+G+B+K+_3	32	42.22	22.67	298	11.8	52.8	28.6	54.2	T ALL (recipient)
	M+G+B+K+_5	47	15.58	9.67	1162	10.3	37.2	16.8	9.03	MDS
	M-G-B+K+_1	46	5.5	2.47	573	13	47.6	11.4	88.8	MDS
	M-G-B+K+_2	46	na	na	na	na	na	na	na	MDS
4th	M+G+B+K+_4	56	4.78	2.74	531	12.1	42.6	na	na	MDS
5th	M+G+K+_3	78	9.42	4.03	796	12.2	41.2	8.9	1.23	MDS
6th	M+G+B+_5-5	74	9.06	3.58	972	9.1	48.5	29.9	74.6	MDS
	M+G+B+_5-3	48	9.54	3.91	1212	12.2	41.2	7.2	76.6	MDS
	M+G+B+_5-4	74	28.24	14.94	311	7.7	56.6	31.7	89.8	AML
	M+G+B+_5-5	52	17.02	10.17	1347	10.6	47.6	10.1	86.1	MDS
7th	M-G-B+K+_1.5-1	41	4.24	0.96	929	10.4	41.4	8.6	20.6	MDS
	M-G-B+K+_1.5-2	40	4.96	1.22	1363	7.3	43.8	32.3	68.9	AML
	M-G-B+K+_1.5-3	44	3.72	0.79	953	9.7	45.2	10.4	69.0	MDS
	M-G-B+K+_1.5-4	48	6.00	1.78	1949	12.8	47.6	11.5	52.9	MDS
	M-G-B+K+_0.5-2	48	3.94	2.03	1218	11.6	43.9	7.1	36.4	MDS
	M-G-B+K+_0.5-5	52	6.46	2.45	1216	10.5	44.2	13.1	36.3	MDS
9th	M+G+B+K+_1	30	12.24	3.4	945	8.5	41.2	8.3	67.3	MDS
	M+G+B+K+_2	52	2.68	2.06	904	8.0	43.7	16.6	50.5	MDS
	M+G+B+K+_4	35	5.64	1.18	304	7.3	50.4	26.4	59.8	AML
	M-G-B+K+_1	52	3.22	1.15	646	7.1	44.1	31.4	42.6	T ALL (immature)
	M-G-B+K+_2	40	258.2	55.33	818	9.4	61.1	89.4	82.8	T ALL (immature)
	M-G-B+K+_3	56	3.84	1.62	726	7.4	49.7	19.7	49.4	AML
	M-G-B+K+_4	60	6.38	2.84	168	4.5	52.3	34.0	54.5	AML
	M-G-B-K+_1	56	55.9	33.93	389	8.1	48.6	59.0	77.4	T ALL (immature)
	M-G-B-K+_2	72	10.16	3.84	1923	10.3	45.3	12.4	31.3	MDS
	M-G-B-K+_4	72	4.74	2.85	501	8.7	45.1	17.9	36.0	MDS
	M-G-B-K+_5	48	7.8	3.36	727	8.3	47.9	34.6	70.4	AML

*Weeks post HSCT. M+, Mac1 positive; G+, Gr1 positive; M-, Mac1 negative; G-, Gr1 negative; B+, B220 positive; K+, Kit positive.

Supplementary Table S9. Acquired mutations in recipient mice transplanted with purified NHD13 BM sub-populations.

ID	Experiment	Donor	Recipient Type	CD45.2 Purity (%)	Diagnosis	Gene	Nucleotide	Amino Acid	VAF
LPK+_1	2nd	5141 LPK	Primary	75	MDS	Mn1	2536G>T	E846*	0.222
						Prrt3	1328A>C	N443T	0.326
						Trbd1 -> (Trbj1-1, 2, 3, 4, 5), (Trbj2-1, 3, 4, 5)[a]			≥0.2
LPK+_2	2nd	5141 LPK	Primary	76.7	MDS	Mn1	3021_3022insAGGG	S1008Efs*12	0.148
LPK+_3	2nd	5141 LPK	Primary	99.0	AML	Kprp	806G>A	R269H	0.217
						Rars2	1268C>T	T423M	0.205
						Dync1li2	1004C>T	A335V	0.211
M+G+_1	2nd	5141 MG	Primary	99.4	MDS	Mn1	2851_2858dup	P954Gfs*6	0.127
						Mn1	3802_3803dup	D1268Efs*30	0.126
						Otulin	717_718del	R239Sfs*7	0.411
						Trbd1 -> Trbj1-4 ; Trbd1 -> Trbj2-5			≥0.2
M+G+_2	2nd	5141 MG	Primary	99.4	MDS	Hoxb2	266dup	A90Sfs*75	0.500
						Mn1	2540_2541dup	D848Pfs*89	0.422
						Pgls	582_583insCCCTC	A195Pfs*20	0.416
M+G+B+K+_1	3rd	5166 MGBK	Primary	79.6	MDS	Cyp4b1	734G>A	R245H	0.321
M+G+B+K+_5	3rd	5166 MGBK	Primary	71.9	MDS	Setd1a	3850_3851 insTC	D1284Vfs*30	0.232
M-G-B+K+_1	3rd	5166 mgBK	Primary	88.8	MDS	Etv6	771_772 ins AGATT	R258*	0.375
						Vmn2r6	1676A>C	H559P	0.376
						Ankzf1	2012T>G	F671C	0.212
						Arap3	4571A>C	Q1524P	0.204
M+G+B+_15-5	6th	5205 MGB	Primary	99.5	MDS	Irf2bp2	1458_1459dup	L487Pfs*77	0.500
						Btbd18	1674G>C	E558D	0.487
						Rapgef1	2215G>C	D739H	0.479
						Kcnf1	1098C>A	F366L	0.291
M+G+B+_5-3	6th	5205 MGB	Primary	99.6	MDS	Btbd18	1674G>C	E558D	0.448
						Rapgef1	2215G>C	D739H	0.469
						Cst13	135G>T	Q45H	0.518
M+G+B+_5-4	6th	5205 MGB	Primary	89.8	AML	Tnfrsf22	88_90dup	L30dup	0.417
						Btbd18	1674G>C	E558D	0.400
						Rapgef1	2215G>C	D739H	0.400
						Kcnk10	1123C>T	R375W	0.517
						Zfp618	1817C>T	S606L	0.491
						C1galt1	329C>T	T110M	0.466
						Sec14l3	953G>A	G318E	0.378
						Fam124a	529C>T	R177C	0.358
						Nol11	2099G>A	R700Q	0.343
M+G+B+_5-5	6th	5205 MGB	Primary	86.1	MDS	Pik3c2a	2441dup	S815Ifs*25	0.313
						Rapgef1	2215G>C	D739H	0.468
						Btbd18	1674G>C	E558D	0.415
						Serpinb5	527C>T	P176L	0.272
M-G-B+K+_1.5-1	7th	5213 mgBK	Primary	98.8	MDS	Tmem266	871C>T	R291C	0.412
						Ptk7	89C>T	P30L	0.375
M-G-B+K+_1.5-2	7th	5213 mgBK	Primary	99.8	AML	Nras	34G>A	G12S	0.436
						Nr0b2	392dup	S131Rfs*33	0.405
						Cfap157	358_359insT	A120Vfs*12	0.419
						Cmtm7	443C>A	A148E	0.322
M-G-B+K+_1.5-3	7th	5213 mgBK	Primary	98.7	MDS	Trp53	716G>A	C239Y	0.250
						Ptk7	89C>T	P30L	0.357
						Pde8b	1625G>A	S542N	0.295
M-G-B+K+_1.5-4	7th	5213 mgBK	Primary	99.7	MDS	Mn1	3812_3819del	A1271Vfs*11	0.247
						Mn1	2514_2542del	K839Qfs*7	0.123
						Tmem266	871C>T	R291C	0.417
						Ptk7	89C>T	P30L	0.533
						Thbs2	1384C>T	R462C	0.272
						Mrpl24	386A>C	K129T	0.210
						Mup10	10A>C	M4L	0.258
M-G-B+K+_0.5-2	7th	5213 mgBK	Primary	99.0	MDS	Ston1	1432C>G	L478V	0.229
						Spty2d1	541G>A	E181K	0.209
						Stac	127C>T	R43W	0.216
M-G-B+K+_0.5-5	7th	5213 mgBK	Primary	98.6	MDS	Mn1	2542_2543insTGGG	D848Gfs*9	0.426
						Tmem266	871C>T	R291C	0.291
						Ptk7	89C>T	P30L	0.344
						Zbtb24	484C>T	R162W	0.377
						Tiam2	2880C>G	N960K	0.330
						Zfp462	4507G>A	V1503I	0.380

Red letters indicate mutations associated with hematologic malignancy. Blue letters highlight mutations present in donor BM. M+, Mac1 positive; G+, Gr1 positive; M-, Mac1 negative; G-, Gr1 negative; B+, B220 positive; K+, Kit positive.
